# Investigation of safety and efficacy of the new more thermostable formulation of Flolan (epoprostenol) in Japanese patients with pulmonary arterial hypertension (PAH)—An open-label, single-arm study

**DOI:** 10.1371/journal.pone.0195195

**Published:** 2018-04-02

**Authors:** Kazuko Mihara, Aiko Ogawa, Hiromi Matsubara, Takumi Terao, Yoshitaka Ichikawa

**Affiliations:** 1 Medicines Development, GlaxoSmithKline K.K., Tokyo, Japan; 2 Department of Clinical Science, National Hospital Organization Okayama Medical Center, Okayama, Japan; 3 Biomedical Data Sciences Department, GlaxoSmithKline K.K., Tokyo, Japan; 4 Project Management Department, GlaxoSmithKline K.K., Tokyo, Japan; Kurume University School of Medicine, JAPAN

## Abstract

**Objective:**

This study was conducted to evaluate the safety and efficacy of a new more thermostable Flolan (epoprostenol) solution prepared with the reformulated pH 12.0 diluent in Japanese patients with pulmonary arterial hypertension (PAH) receiving higher doses of Flolan than those typically administered in Western countries.

**Methods:**

This open-label, single-arm study was conducted in 10 Japanese PAH patients. During the run-in period, patients were intravenously infused with Flolan (45 ng/kg/min or higher doses) solution prepared with the existing pH 10.5 diluent. The patients were then switched to a new more thermostable Flolan solution prepared with the reformulated pH 12.0 diluent and observed for a 4-week treatment period. As a primary endpoint, safety after switching to the new Flolan solution was evaluated. Secondary endpoints included hemodynamics and the necessity for dose adjustment of Flolan in these patients.

**Results:**

All 10 patients completed the study period. Observed adverse events were nausea and hepatic function abnormal in 1 patient each, and both events were mild. No patients required dose adjustment due to the change from baseline in mean pulmonary artery pressure (mPAP) measured 3 hrs after switching to Flolan solution prepared with the reformulated diluent. No major changes from baseline in mPAP, pulmonary vascular resistance, or right atrial pressure were observed at 24 hrs and at 4 weeks after switching to the Flolan solution prepared with pH 12.0 diluent. Although some patients showed increases in cardiac output (CO) from baseline at 24 hrs and 4 weeks, no patients required dose reduction as a result of an increase in CO.

**Conclusion:**

Neither safety/efficacy concerns nor any dose adjustments of Flolan after switching to a more thermostable Flolan solution prepared with the reformulated pH 12.0 diluent could be required in Japanese patients with PAH receiving higher doses of Flolan.

**Trial registration:**

ClinicalTrials.gov Identifier: NCT02705807

## Introduction

Pulmonary arterial hypertension (PAH) is a rare disease characterized by increased pulmonary artery pressure (PAP), leading to right heart failure and death [1]. Several studies have shown Flolan (epoprostenol sodium) to alleviate PAH symptoms and decrease PAH-related mortality [2, 3, 4, 5]. Flolan has been used as a therapeutic agent for PAH in many countries including the US and the EU and was approved in Japan in 1999 [6]. Flolan, when prepared with pH 12.0 diluent, is more thermostable. The pH 12.0 diluent was approved in Japan in April 2016. Flolan solution prepared with the existing pH 10.5 diluent should be administered within a 24 hr period after preparation and maintained within a temperature range of 2°C to 8°C during infusion, necessitating the use of a cold pack. In addition, the cold pack that is used to maintain the temperature of the Flolan solution requires frequent changes. Frequent preparation of Flolan solution and cold pack use may place a substantial burden on patients with PAH.

The stability of Flolan solution is improved at ambient temperatures by increasing the pH of the diluent used to prepare the solution from 10.5 (existing diluent in the range of pH 10.2 to 10.8) to 12.0 (reformulated diluent in the range of pH 11.7 to 12.3). The Flolan solution freshly prepared with the reformulated pH 12.0 diluent can be stored at 2°C to 8°C for up to 8 days, and can be administered up to 24 hrs at up to 35°C or up to 72 hrs at up to 25°C. Therefore, the use of a cold pack and frequent changes of the medication cassette are not required, hopefully leading to increased convenience for patients with PAH. The change in the new Flolan formulation is limited to the diluent: the amount and formulation of the active ingredient, epoprostenol, remains the same. Therefore, the new formulation is not expected to impact either the safety or the efficacy of Flolan, and its clinical profile is expected to be the same as that prepared with the existing diluent formulation. In fact, a recent study of Flolan conducted at centers in Western countries demonstrated that no dose adjustment was required for switching from solution prepared with the existing diluent to solution prepared with the reformulated diluent and that, furthermore, the safety and tolerability of Flolan solution prepared with the reformulated diluent were similar in 16 Caucasian PAH patients [7]. However, experience in Japanese PAH patients with switching to the new Flolan solution prepared with the reformulated diluent is limited. Moreover, the study focused only on non-invasive parameters and did not include measurement of hemodynamic parameters, which are important for evaluating condition and severity of PAH. Additionally, the mean dose of Flolan administered to patients included in the study was relatively low (36 ng/kg/min), compared to the dose used for Japanese patients with PAH [8, 9, 10]. It is unknown whether higher concentration of epoprostenol in the Flolan solution prepared with the reformulated diluent required dose adjustment.

The objective of this study was to evaluate the safety, efficacy on hemodynamic parameters, and necessity of dose adjustment after switching to Flolan solution prepared with the reformulated diluent in Japanese patients with PAH receiving higher doses of Flolan.

## Materials and methods

The protocol for this trial and supporting TREND checklist are available as supporting information; see S1 Protocol and S1 TREND Checklist.

### 1. Patients

This single-center, open-label, single-arm phase 4 study was conducted in 10 Japanese patients with PAH between May 2016 and July 2016 (ClinicalTrials.gov identifier: NCT02705807). Patients were eligible for inclusion in this study only if all of the following inclusion criteria were met and none of the exclusion criteria were present.

Main inclusion criteria: 1) male or female Japanese patients at least 18 to 75 years of age at the time of screening, 2) had to be on Flolan therapy for PAH, 3) receiving Flolan at 45 ng/kg/min or higher for PAH, 4) had to be on stable doses of their Flolan treatment with existing diluent for a minimum of one month prior to screening though it was acceptable to adjust within 10% of dose during the last one-month period, and 5) had to be on stable doses of any current PAH treatments other than Flolan in the 30 days prior to screening. The main exclusion criteria: 1) receiving Flolan for a condition or in a manner outside the approved indications, 2) congestive heart failure arising from severe left ventricular dysfunction, 3) a resting arterial oxygen saturation (SaO_2_) < 90% as measured by pulse oximetry at screening, regardless of whether or not supplemental oxygen was provided, 4) emergently hospitalized or visited the emergency room for a condition related to PAH or additional treatment for PAH in the prior 3 months, 5) suffering from a clinical condition not expected to remain stable for the duration of the study, and 6) having participated in a clinical study involving another investigational drug or device within 4 weeks before screening.

### 2. Ethics statement

This study was conducted in accordance with the Declaration of Helsinki [11], the International Conference on Harmonization of Good Clinical Practice Guidelines [12]. The investigators obtained written informed consent from all patients before their recruitment to the study. The study protocols were approved by the institutional review board at the National Hospital Organization Okayama Medical Center, Japan, before the initiation of this study.

### 3. Study design

**Fig 1:**
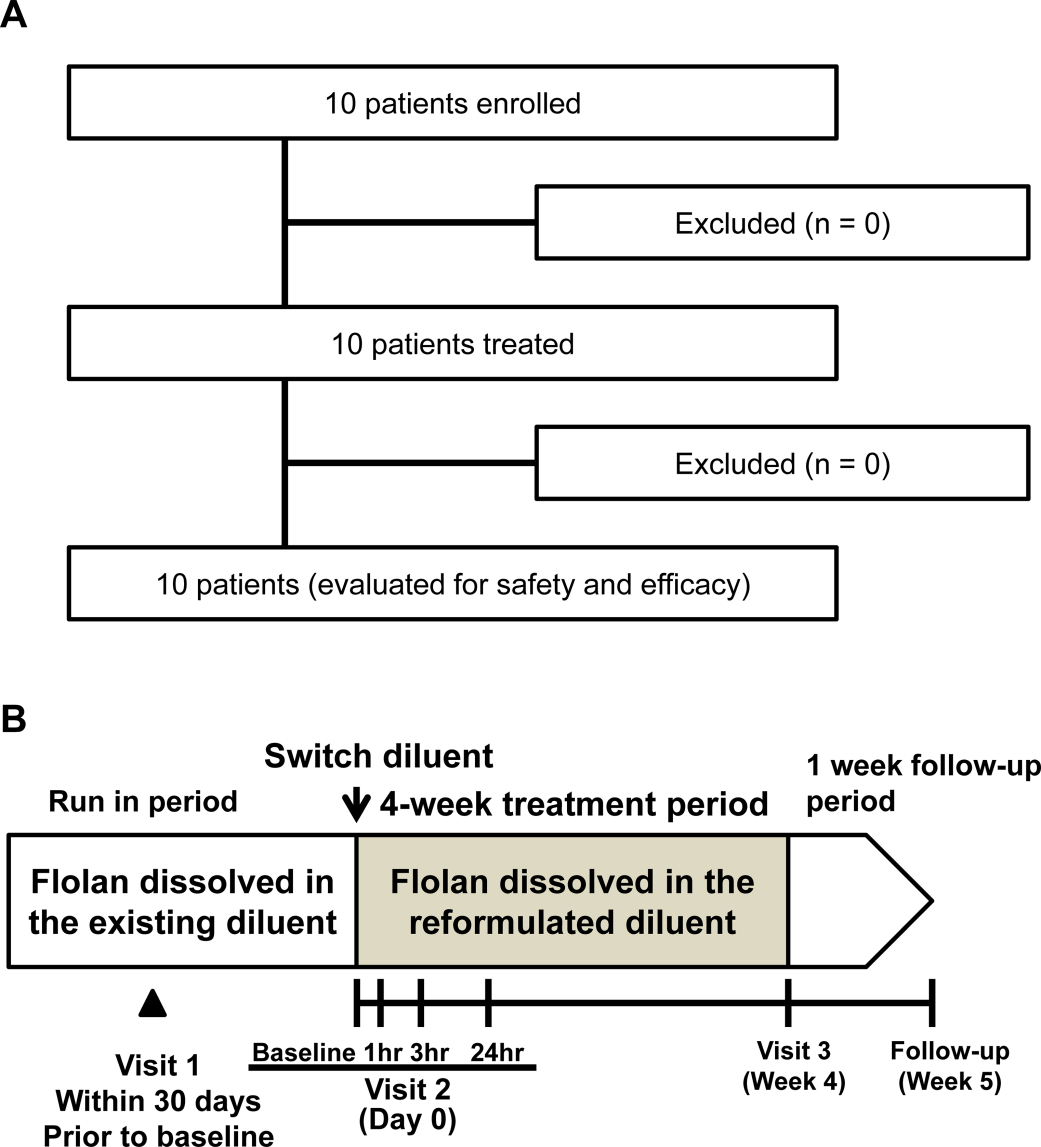
Patient disposition and study design. (A) Patient disposition: Ten patients were enrolled and entered the study during the run-in period. All patients completed the treatment and follow-up periods. (B) Study design: The study consisted of 3 periods: 1) a run-in period when patients were treated with the Flolan solution prepared with the existing diluent for up to 30 days, 2) a 4-week treatment period with the new Flolan solution prepared with the reformulated diluent, and 3) a 1-week follow-up period. The patients were continuously treated the new Flolan solution prepared with the reformulated diluent during the follow-up period.

The patient disposition and design of this study is shown in Fig 1A and 1B, respectively. A total 10 patients were enrolled, and they were fixed as safety and efficacy analysis populations. The study consisted of 3 periods: 1) a run-in period when patients were treated with Flolan solution prepared with an existing diluent for up to 30 days, 2) a 4-week treatment period with Flolan solution prepared with the reformulated diluent, and 3) a 1-week follow-up period. Ten patients who fulfilled all inclusion criteria and met none of the exclusion criteria at screening (Visit 1) were included in the run-in period. Patients continuously received Flolan at 45 ng/kg/min or higher doses via a central venous catheter using a portable infusion pump during the run-in, treatment, and follow-up periods. After the run-in period, patients underwent baseline assessments at the National Hospital Organization Okayama Medical Center and switched to Flolan solution prepared with the reformulated diluent. Patients stayed overnight for assessment of clinical symptoms and hemodynamic parameters up to 24 hrs after switching to Flolan solution prepared with the reformulated diluent at the study center (Visit 2).

All patients underwent right heart catheterization (RHC) up to 24 hrs and 4 weeks after switching to the Flolan solution prepared with the reformulated diluent. Safety endpoints were evaluated during the treatment and follow-up periods. Efficacy endpoints were also evaluated during the treatment period.

### 4. Investigational drug

GlaxoSmithKline K.K. (Tokyo, Japan) provided only the reformulated diluent for Flolan used during the treatment period. Vials of Flolan lyophile (epoprostenol 0.5 mg and 1.5 mg) used during all of the study periods and the existing diluent of Flolan used during the run-in period were products already on the market. Since the reformulated diluent became commercially available in June 2016, we used this newly marketed product during the follow-up period. To prepare the Flolan solution, patients diluted 1 or more vials of Flolan lyophile with 2 vials of the reformulated diluent, producing 100 mL of medication for each day of the treatment period. Medication cassettes were filled with this reconstituted formulation prior to either refrigerated storage or immediate continuous intravenous infusion of Flolan.

PAH standard treatments that could be used in combination were as follows, but not limited to them: vasodilators such as angiotensin-converting enzymes inhibitors and calcium channel blockers, cyclic guanosine monophosphate-specific phosphodiesterase type 5 inhibitors such as sildenafil citrate and tadalafil, and endothelin receptor antagonist such as ambrisentan and bosentan hydrate. During the study, no other prostanoids or their inducers were permitted.

### 5. Safety evaluation

The incidence of adverse events (AEs), clinical laboratory data, 12-lead electrocardiogram (ECG), vital signs, and oxygen saturation were evaluated for the primary endpoint. Multiple occurrences of the same events in 1 patient were counted only once. Individual AEs were coded by MedDRA version 19.0 and classified according to System Organ Class and Preferred Term. Clinical laboratory (hematological and biochemical) values were measured at the screening visit, baseline, and 4 weeks after switching to Flolan solution prepared with the reformulated diluent. Vital signs (systolic and diastolic blood pressures, and pulse rate) and oxygen saturation were measured at screening, baseline, 1, 3, 24 hrs, and 4 weeks after switching. The 12-lead ECG was obtained during the run-in period, and at baseline, 24 hrs, and 4 weeks after switching.

### 6. Efficacy evaluation

Secondary endpoints were evaluated: 1) the number of Flolan dose adjustments based on the change in mPAP from baseline at 3 hrs after switching to the new Flolan solution prepared with the reformulated diluent, 2) the reason for the Flolan dose adjustments after switching to the Flolan solution prepared with the reformulated diluent, 3) change in N-terminal pro-B-type natriuretic peptide (NT-proBNP) from baseline to 4 weeks after switching to Flolan solution prepared with the reformulated diluent, 4) World Health Organization (WHO) functional class by visits and change from previous visit, and 5) changes in hemodynamic parameters: mPAP, right atrial pressure (RAP), pulmonary vascular resistance (PVR), and cardiac output (CO) from baseline to 24 hrs and at 4 weeks after switching to Flolan solution prepared with the reformulated diluent.

Hemodynamic parameters such as mPAP, RAP, PVR, and CO were evaluated by RHC test at 1, 3, and 24 hrs, and at 4 weeks after switching to Flolan solution prepared with the reformulated diluent. The WHO functional classification was evaluated at screening, baseline, and 4 weeks after switching to Flolan solution prepared with the reformulated diluent. Blood samples were collected at baseline, 24 hrs, and 4 weeks after switching to Flolan solution prepared with the reformulated diluent in order to measure the plasma concentration of NT-proBNP.

### 7. Statistical analysis

The sample size (10 patients) for this study was determined based on the feasibility of performing an exploratory study, and not hypothesis testing. Therefore, there was no sample size rationale and the formal statistical analysis was not conducted. All the 10 patients completed the 4-week treatment and 1-week follow-up periods in this study. Data were summarized by using descriptive statistics. The intent-to-treat (ITT) population was selected as the group to be analyzed. The ITT population comprised patients who received at least 1 dose of Flolan solution prepared with the reformulated diluent. The ITT population was used for all safety and efficacy evaluations. As primary endpoints, frequency tabulation or summary statistics (AEs, clinical laboratory [hematology, clinical chemistry, and urinalysis] values, vital signs [systolic and diastolic blood pressures, pulse rate, and body weight], 12-lead ECG findings, and oxygen saturation) were calculated for safety. As secondary endpoints, the frequency of Flolan dose adjustments, the frequency of WHO functional class by visits, and the change from the previous visit were calculated. Summary statistics were calculated for the measured values and changes from baseline in NT-proBNP and the hemodynamic parameters (mPAP, RAP, PVR and CO).

## Results

### 1. Patient disposition

The patient disposition is shown in Fig 1A. Ten patients were enrolled and entered the study during the run-in period. All patients completed the treatment and follow-up periods. No patients were withdrawn from the study. Also, no significant deviations from the study protocol or deviations from the selection/exclusion criteria were reported.

### 2. Patient characteristics

Patient characteristics are shown in Table 1. There were 9 females and 1 male. The mean age was 35.8 years, with a range of 26 to 44 years. All patients were diagnosed with PAH by the RHC test. The mean duration since being diagnosed with PAH was 9.70 years, with a range of 1.0 to 18.4 years. The diagnoses of PAH were classified into idiopathic PAH (3 patients), familial PAH (6 patients), and PAH associated with congenital heart disease (1 patient). In addition, the WHO functional class of patients at screening and baseline were I (1 patient) and II (9 patients). All patients continued the same dose of PAH therapeutic agents except Flolan used before recruitment for this study, with no changes during the treatment period. The PAH therapeutic agents used in combination were bosentan hydrate (8 patients), tadalafil (7 patients), macitentan (1 patient), and sildenafil citrate (1 patient).

**Table 1:** Patient characteristics.

Parameters	New diluent (n = 10)
Sex, n (%)	
Female	9 (90%)
Male	1 (10%)
Age (years)	
Mean (SD)	35.8 (7.35)
Median (Min., Max.)	36.0 (26, 44)
Height (cm)	
Mean (SD)	155.50 (10.058)
Median (Min., Max.)	154.00 (146.0, 179.0)
Weight (kg)	
Mean (SD)	47.86 (11.172)
Median (Min., Max.)	46.25 (36.2, 71.0)
History of tobacco use, n (%)	
Current smoker	1 (10%)
Former smoker	3 (30%)
Never smoked	6 (60%)
CV family history, n (%)	
Yes	0
No	10 (100%)
Years since diagnosed with PAH (years)	
Mean (SD)	9.70 (5.706)
Median (Min. Max.)	7.84 (1.0, 18.4)
WHO functional class, n (%)	
Class I	1 (10%)
Class II	9 (90%)
Class III	0
Class IV	0
Classification of PAH, n (%)	
Idiopathic PAH	3 (30%)
Familial PAH	6 (60%)
PAH associated with congenital heart disease	1 (10%)
Hemodynamic parameters at baseline	
mPAP (mmHg) (SD)	32.7 (14.13)
RAP (mmHg) (SD)	3.7 (2.11)
PVR (mmHg/L/min) (SD)	4.489 (1.7968)
CO (L/min) (SD)	5.680 (1.4741)

n: number of patient, SD: standard deviation, PAH: pulmonary arterial hypertension, CV: cardiovascular, WHO: World Health Organization, mPAP: mean pulmonary artery pressure, RAP: right atrial pressure, PVR: pulmonary vascular resistance, CO: cardiac output.

### 3. Flolan exposure

The status of exposure to the investigational drug is shown in Table 2. The mean dose of Flolan for 1 hr from switching to the new Flolan solution prepared with the reformulated diluent was 83.28 ng/kg/min, with a range of 50.5 to 113.8 ng/kg/min. The mean doses used continued to be generally similar throughout the treatment period. The mean duration of Flolan treatment through the treatment period was 28.2 days, with a range of 26 to 32 days.

**Table 2:** Doses of Flolan after switching to Flolan solution prepared with the reformulated diluent (ITT population).

Time after switching	dose (ng/kg/min)
0–1 hr	
Mean (SD)	83.28 (21.297)
Median (Min., Max.)	79.85 (50.5, 113.8)
1–3 hr	
Mean (SD)	82.56 (21.907)
Median (Min., Max.)	77.85 (47.6, 113.8)
3–24 hr	
Mean (SD)	82.56 (21.907)
Median (Min., Max.)	77.85 (47.6, 113.8)
24 hr-4 weeks	
Mean (SD)	82.56 (21.907)
Median (Min., Max.)	77.85 (47.6, 113.8)
Duration of Flolan treatment during the treatment period (days)	
Mean (SD)	28.2 (1.87)
Median (Min., Max.)	28.5 (26, 32)

ITT: intent-to-treat, SD: standard deviation.

### 4. Safety

An overview of AEs is shown in Table 3. AEs that occurred during the treatment period were nausea and hepatic function abnormal in 1 patient each, and both were mild. Nausea was considered to be drug-related by the investigator. Nausea resolved during the treatment period, and hepatic functional abnormal was observed at 4 weeks during the treatment period.

**Table 3:** Overview of the incidence of AEs.

	n (%)
Total number of patients	10 (100%)
Any AE	2 (20%)
Any serious AE	0
Any AE leading to death	0
Discontinued treatment due to AE	0
AEs	
Nausea	1 (10%)
Hepatic function abnormal	1 (10%)

AE: adverse event.

No deaths or AEs leading to discontinuation of the investigational drug were reported. Also, no cardiovascular events were observed. As to the other clinical chemistry tests including thyroid function, hematology tests, and urinalysis, no obvious changes from the baseline values were detected at 4 weeks after switching to Flolan solution prepared with the reformulated diluent. As to vital signs, no effects on systolic and diastolic blood pressures or heart rate were observed. There were no abnormal findings on the 12-lead ECG at 4 weeks after switching to Flolan solution prepared with the reformulated diluent. There was no obvious change from baseline in oxygen saturation at any of the evaluation time points after the baseline measurement.

### 5. Efficacy

#### Dose adjustments after switching to Flolan solution prepared with the reformulated diluent

No dose adjustments were performed based on mPAP changes from baseline, as determined at 3 hrs after switching to Flolan solution prepared with the reformulated diluent. In this study, the dose of Flolan was slightly reduced in 2 patients at 1 hr after switching to Flolan solution prepared with the reformulated diluent by the investigator due to increased CO (1 patient: 80.3 to 76.0 ng/kg/min, 1 patient: 50.5 to 47.6 ng/kg/min).

#### Changes from baseline in hemodynamic parameters after switching to Flolan solution prepared with the reformulated diluent

**Fig 2:**
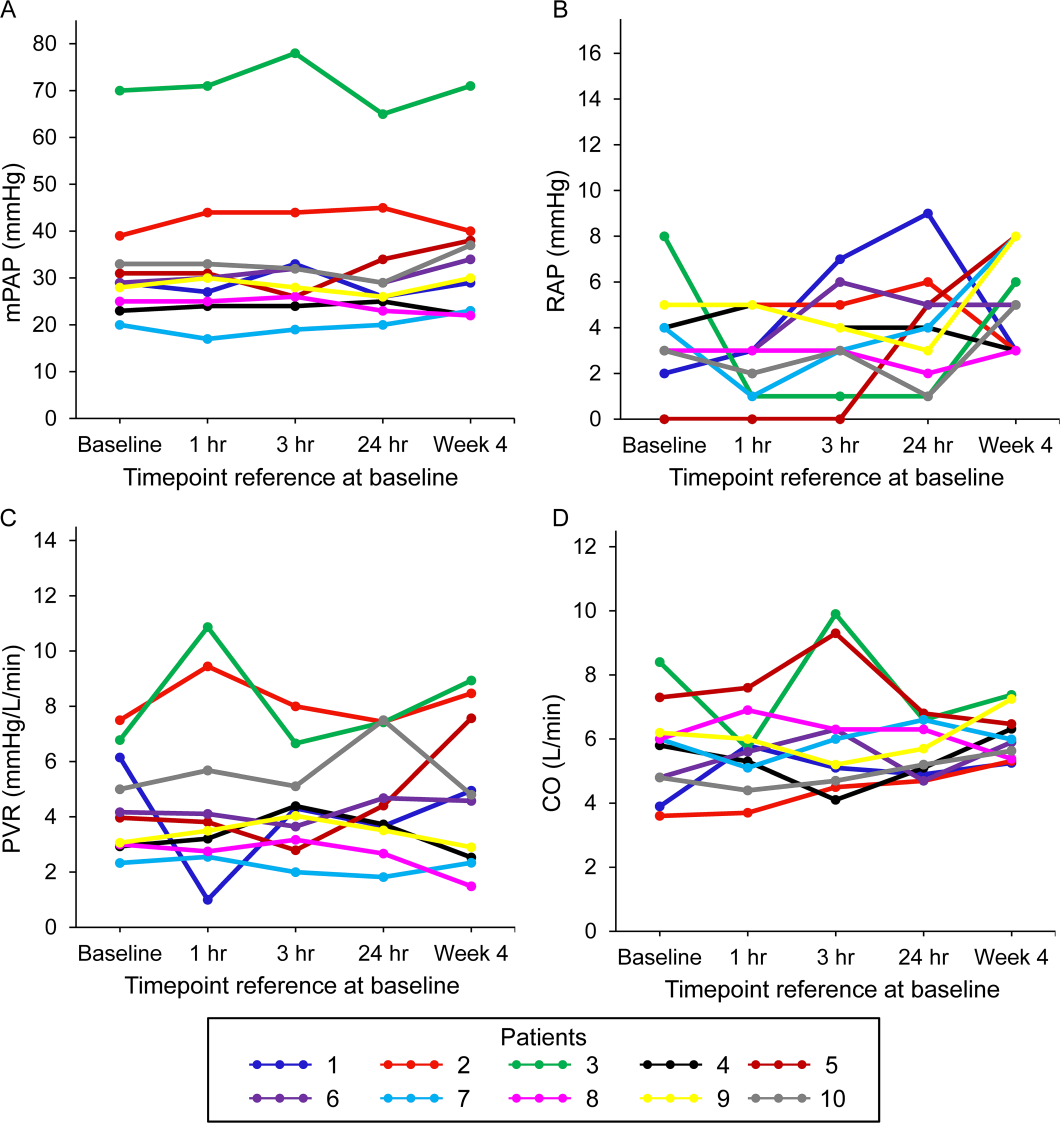
Changes from baseline in mPAP, RAP, PVR, and CO after switching in individual patients (n = 10). mPAP: mean pulmonary artery pressure, RAP: right atrial pressure, PVR: pulmonary vascular resistance, CO: cardiac output.

Changes from baseline in mPAP, RAP, PVR, and CO are shown in Table 4 and Fig 2. No significant changes from baseline in mPAP, RAP, PVR, and CO were observed at 1, 3, and 24 hrs or at 4 weeks after switching to Flolan solution prepared with the reformulated diluent.

**Table 4:** Changes from baseline in mPAP, RAP, PVR, and CO after switching.

Parameter	n	Visit	Mean (SD)	Median (Min., Max.)
Values of hemodynamic parameters at various measurement points				
mPAP (mmHg)	10	Baseline	32.7 (14.13)	29.0 (20, 70)
		1 hr	33.2 (14.99)	30.0 (17, 71)
		3 hr	34.2 (16.78)	30.0 (19, 78)
		24 hr	32.2 (13.44)	27.5 (20, 65)
		Week 4	34.6 (14.44)	32.0 (22, 71)
RAP (mmHg)	10	Baseline	3.7 (2.11)	3.5 (0, 8)
		1 hr	2.8 (1.81)	3.0 (0, 5)
		3 hr	3.6 (2.12)	3.5 (0, 7)
		24 hr	4.0 (2.45)	4.0 (1, 9)
		Week 4	5.2 (2.20)	5.0 (3, 8)
PVR (mmHg/L/min)	10	Baseline	4.489 (1.7968)	4.070 (2.33, 7.50)
		1 hr	4.693 (3.1359)	3.655 (1.00, 10.87)
		3 hr	4.412 (1.7992)	4.175 (2.00, 8.00)
		24 hr	4.685 (2.0726)	4.070 (1.82, 7.50)
		Week 4	4.857 (2.6685)	4.690 (1.49, 8.94)
CO (L/min)	10	Baseline	5.680 (1.4741)	5.900 (3.60, 8.40)
		1 hr	5.610 (1.1200)	5.650 (3.70, 7.60)
		3 hr	6.140 (1.9744)	5.600 (4.10, 9.90)
		24 hr	5.660 (0.8449)	5.450 (4.70, 6.80)
		Week 4	6.088 (0.7661)	5.945 (5.25, 7.38)
Change from baseline in hemodynamic parameters after switching				
mPAP (mmHg)	10	1 hr	0.5 (2.17)	0.5 (-3, 5)
		3 hr	1.5 (3.66)	1.0 (-5, 8)
		24 hr	-0.5 (3.41)	-1.0 (-5, 6)
		Week 4	1.9 (2.96)	1.5 (-3, 7)
RAP (mmHg)	10	1 hr	-0.9 (2.42)	0.0 (-7, 1)
		3 hr	-0.1 (3.07)	0.0 (-7, 5)
		24 hr	0.3 (3.89)	0.0 (-7, 7)
		Week 4	1.5 (3.06)	1.5 (-2, 8)
PVR (mmHg/L/min)	10	1 hr	0.204 (2.2976)	0.250 (-5.15, 4.09)
		3 hr	-0.077 (0.9716)	-0.005 (-1.84, 1.46)
		24 hr	0.196 (1.2514)	0.445 (-2.48, 2.50)
		Week 4	0.368 (1.5393)	-0.075 (-1.51, 3.60)
CO (L/min)	10	1 hr	-0.070 (1.2320)	-0.050 (-2.70, 1.90)
		3 hr	0.460 (1.1843)	0.600 (-1.70, 2.00)
		24 hr	-0.020 (0.8854)	0.100 (-1.80, 1.10)
		Week 4	0.408 (0.9700)	0.675 (-1.02, 1.71)

mPAP: mean pulmonary artery pressure, RAP: right atrial pressure, PVR: pulmonary vascular resistance, CO: cardiac output, SD: standard deviation

#### Change of WHO functional class after switching to Flolan solution prepared with the reformulated diluent

Nine of 10 patients were evaluated by the investigator at screening as WHO functional class II, and no change was observed at baseline. One patient was evaluated as class I by the investigator at screening and at baseline, but the evaluation was class II 4 weeks after switching to Flolan solution prepared with the reformulated diluent.

#### Change from baseline in NT-proBNP concentration measured at 24 hrs and 4 weeks after switching to Flolan solution prepared with the reformulated diluent

Changes from baseline in plasma NT-proBNP concentration at 24 hrs and 4 weeks after switching to Flolan solution prepared with the reformulated diluent are shown in Table 5. No change from baseline in the plasma NT-proBNP concentration was observed at 24 hrs after switching to Flolan solution prepared with the reformulated diluent. The mean change from the baseline in the plasma NT-proBNP concentration at 4 weeks after switching was 33.7 ng/L, a slight increase from the baseline, but no significant changes were seen. In 1 patient, an increase from baseline in the plasma NT-proBNP concentration was observed at 4 weeks after switching (baseline: 269 ng/L, at 4 weeks after switching: 581 ng/L). In another patient, an increase from baseline was observed 24 hrs after switching (baseline: 35 ng/L, 24 hrs after switching: 70 ng/L), and this change was judged by the investigator to be of minimal clinical significance. There were no notable changes in any of the other patients.

**Table 5:** Change from baseline in NT-ProBNP.

		NT-ProBNP (ng/L)	
Time after switching	n	Mean (SD)	Median (Min., Max.)
Baseline	10	92.8 (81.58)	76.5 (11, 269)
24 hr	10	91.0 (76.94)	69.0 (16, 262)
4 week	10	126.5 (163.07)	93.5 (25, 581)
Change from baseline			
24 hr	10	-1.8 (21.14)	-6.5 (-38, 35)
4 week	10	33.7 (103.66)	18.0 (-66, 312)

NT-ProBNP: N-terminal pro-B-type natriuretic peptide, SD: standard deviation.

## Discussion

This study was conducted to evaluate the safety, efficacy, and necessity of dose adjustment after switching to a more thermostable Flolan solution prepared with the reformulated diluent in Japanese patients with PAH receiving higher doses of Flolan than those used in Western countries.

The mean dose of Flolan for 1 hr from switching to Flolan solution prepared with the reformulated diluents was 83.28 ng/kg/min. The doses of Flolan were similar throughout the treatment period. These were higher as compared with those administered overseas, generally 20 to 40 ng/kg/min, or the western study, 36 ng/kg/min [7, 13, 14].

As to safety, the results obtained in the present study revealed no unknown safety concerns associated with switching to Flolan solution prepared with the reformulated diluent in Japanese PAH patients receiving higher-dose Flolan.

As to efficacy, given that mPAP measurements and changes are important as hemodynamic parameters reflecting the therapeutic efficacy of investigational drugs and PAH disease conditions, the frequency of and reasons for Flolan dose adjustments based on changes from baseline in mPAP were established as secondary endpoints. In this study, there were essentially no changes in mPAP and only small changes in other hemodynamic parameters including PVR and RAP, suggesting minimal impact of switching to Flolan solution prepared with the reformulated diluent. Inter-patient variations in changes from baseline in the NT-proBNP concentrations at 4 weeks after switching to Flolan solution prepared with the reformulated diluent were observed, but there seemed to be no noteworthy changes. One patient had WHO functional class of change, which was considered by the investigator to be an individual variability rather than being due to disease progression. These results suggested that there would be neither dose adjustments nor any impacts on efficacy of switching to Flolan solution prepared with the reformulated diluent in Japanese PAH patients receiving higher-dose Flolan.

The limitation of this study is that there were only 10 patients. This was an exploratory study, and secondary endpoints such as hemodynamic parameters, NT-proBNP, and WHO functional class were not statistically analyzed due to the small sample size. Therefore, its evidence level is not comparable to that of other clinical studies with more patients that have performed formal statistical analysis. We could not draw definitive conclusions about the safety and efficacy of a new Flolan prepared with the pH 12.0 diluent.

Use of the pH 10.5 diluent to prepare Flolan solution necessitates frequent preparation and the use of cold packs by patients due to the thermo-instability of epoprostenol, thus placing a substantial burden on patients with PAH, especially those with a low quality of life and/or complications. Flolan solution prepared with the pH 12.0 diluent allows drug administration without frequent preparation or the use of cold packs. This study suggests that Flolan solution prepared with the reformulated diluent could provide a convenient treatment option for patients while maintaining efficacy and safety.

## Conclusion

Neither safety/efficacy concerns nor dose adjustments of Flolan after switching to the new more thermostable Flolan solution prepared with the reformulated pH 12.0 diluent were required in Japanese patients with PAH receiving higher doses of Flolan than those generally used in Western countries.

## Supporting information

S1 TREND Checklist TREND checklist. (PDF)

S1 Protocol Trial protocol. (PDF)
