# Supplementary material for: Investigation of safety and efficacy of the new more thermostable formulation of Flolan (epoprostenol) in Japanese patients with pulmonary arterial hypertension (PAH)—An open-label, single-arm study
Source: PLoS One. 2018 Apr 2;13(4):e0195195. doi: 10.1371/journal.pone.0195195 (PMC5880383; doi:10.1371/journal.pone.0195195)
Supplement: S1 Protocol — (PDF) [file pone.0195195.s002.pdf]

TITLE PAGE

Division: Worldwide Development

Information Type: Clinical Protocol

Title: An Open Label, Single-arm Study Evaluating a New Thermostable Formulation of FLOLAN™ in Japanese Subjects with Pulmonary Arterial Hypertension (PAH)

Compound Number: AH21461

Development Phase: IV

Effective Date: 03-DEC-2015

Author(s): PPD

Copyright 2015 the GlaxoSmithKline group of companies. All rights reserved. Unauthorised copying or use of this information is prohibited.

SPONSOR SIGNATORY:

PPD

______________________________ 2015/12/03
Hiromu Nakajima PPD Date
Head, Medicines Dev PPD ent

MEDICAL MONITOR/SPONSOR INFORMATION PAGE

Medical Monitor/SAE Contact Information:

Role	Name	Day Time Phone Number and email address	After-hours Phone/Cell/ Pager Number	Fax Number	Site Address
Primary Medical Monitor and SAE contact information	PPD	PPD<br>PPD	Not applicable	PPD<br>PPD	6-15, Sendagaya 4-chome, Shibuya-ku, Tokyo 151-8566
Secondary Medical Monitor	PPD	PPD<br>PPD	Not applicable	PPD<br>PPD	6-15, Sendagaya 4-chome, Shibuya-ku, Tokyo 151-8566

Sponsor Legal Registered Address:

GlaxoSmithKline K.K. (GSK Japan)
6-15, Sendagaya 4-chome

Shibuya-ku, Tokyo 151-8566, Japan
Study Director: PPD Head, MPC TA Office, Medicines Development

Regulatory Agency Identifying Number(s): Not applicable

INVESTIGATOR PROTOCOL AGREEMENT PAGE

- I confirm agreement to conduct the study in compliance with the protocol.
- I acknowledge that I am responsible for overall study conduct. I agree to personally conduct or supervise the described study.
- I agree to ensure that all associates, colleagues and employees assisting in the conduct of the study are informed about their obligations. Mechanisms are in place to ensure that site staff receives the appropriate information throughout the study.

Investigator Name:

Study Centre Address:

Study Centre Phone Number:

Investigator Signature: Date:

TABLE OF CONTENTS

PAGE

1. PROTOCOL SYNOPSIS FOR STUDY FLR201614 8

2. INTRODUCTION 11
   2.1. Study Rationale 11
   2.2. Brief Background 11

3. OBJECTIVES AND ENDPOINTS 12

4. STUDY DESIGN 13
   4.1. Overall Design 13
   4.2. Treatment Arms and Duration 13
   4.3. Type and Number of Subjects 14
   4.4. Design Justification 14
   4.5. Dose Justification 15
   4.6. Benefit:Risk Assessment 15
      4.6.1. Risk Assessment 16
      4.6.2. Benefit Assessment 19
      4.6.3. Overall Benefit:Risk Conclusion 19

5. SELECTION OF STUDY POPULATION AND WITHDRAWAL CRITERIA 19
   5.1. Inclusion Criteria 19
   5.2. Exclusion Criteria 21
   5.3. Screening/Baseline/Run-in Failures 22
   5.4. Withdrawal/Stopping Criteria 22
      5.4.1. Liver Chemistry Stopping Criteria 23
   5.5. Subject and Study Completion 24

6. STUDY TREATMENT 24
   6.1. Investigational Product 24
   6.2. Treatment Assignment 24
   6.3. Subject Specific Dose Adjustment Criteria 25
   6.4. Blinding 25
   6.5. Packaging and Labeling 25
   6.6. Preparation/Handling/Storage/Accountability 25
   6.7. Compliance with Study Treatment Administration 26
   6.8. Treatment of Study Treatment Overdose 26
   6.9. Treatment after the End of the Study 27
   6.10. Concomitant Medications and Non-Drug Therapies 27
      6.10.1. Permitted Medications and Non-Drug Therapies 27
      6.10.2. Prohibited Medications and Non-Drug Therapies 27

7. STUDY ASSESSMENTS AND PROCEDURES 28
   7.1. Time and Events Table 29
   7.2. Screening and Critical Baseline Assessments 32
   7.3. Efficacy 32
      7.3.1. Haemodynamics 32
      7.3.2. WHO Functional Class 33
      7.3.3. N-terminal Pro B-type Natriuretic Peptide: NT-pro BNP 33
   7.4. Safety 33

7.4.1. Adverse Events (AE) and Serious Adverse Events (SAEs) 33
7.4.1.1. Time Period and Frequency for Collecting AE and SAE Information 33
7.4.1.2. Method of Detecting AEs and SAEs 34
7.4.1.3. Follow-up of AEs and SAEs 34
7.4.1.4. Cardiovascular and Death Events 35
7.4.1.5. Regulatory Reporting Requirements for SAEs 35
7.4.2. Pregnancy 35
7.4.3. Physical Exams 36
7.4.4. Vital Signs 36
7.4.5. Oxygen Saturation 36
7.4.6. Electrocardiogram (ECG) 36
7.4.7. Clinical Safety Laboratory Assessments 36
7.5. Other Endpoints 37
7.5.1. Reasons for Dose Adjustment after Switch 37

8. DATA MANAGEMENT 37

9. STATISTICAL CONSIDERATIONS AND DATA ANALYSES 37
9.1. Hypotheses 37
9.2. Sample Size Considerations 38
9.2.1. Sample Size Assumptions 38
9.2.2. Sample Size Sensitivity 38
9.2.3. Sample Size Re-estimation or Adjustment 38
9.3. Data Analysis Considerations 38
9.3.1. Analysis Populations 38
9.3.2. Analysis Data Sets 38
9.3.3. Treatment Comparisons 38
9.3.4. Interim Analysis 38
9.4. Key Elements of Analysis Plan 39
9.4.1. Primary Analyses 39
9.4.2. Secondary Analyses 39
9.4.3. Other Analyses 40

10. STUDY GOVERNANCE CONSIDERATIONS 40
10.1. Posting of Information on Publicly Available Clinical Trial Registers 40
10.2. Regulatory and Ethical Considerations, Including the Informed Consent Process 40
10.3. Quality Control (Study Monitoring) 41
10.4. Quality Assurance 41
10.5. Study and Site Closure 42
10.6. Records Retention 42
10.7. Provision of Study Results to Investigators, Posting of Information on Publically Available Clinical Trials Registers and Publication 43

11. REFERENCES 44

12. APPENDICES 45
12.1. Appendix 1 – Abbreviations and Trademarks 45
12.2. Appendix 2: Liver Safety Required Actions and Follow up Assessments 47

12.3. Appendix 3: Definition of and Procedures for Recording, Evaluating, Follow-Up and Reporting of Adverse Events....49
12.3.1. Definition of Adverse Events....49
12.3.2. Definition of Serious Adverse Events....50
12.3.3. Definition of Cardiovascular Events....52
12.3.4. Recording of AEs and SAEs....53
12.3.5. Evaluating AEs and SAEs....53
12.3.6. Reporting of SAEs to GSK....55
12.4. Appendix 4: Collection of Pregnancy Information....56
12.5. Appendix 5 - Japan Specific Requirements....58
12.5.1. Regulatory and Ethical Considerations....58
12.5.2. Informed Consent....58
12.5.3. Study Period....58
12.6. Appendix 6 - List of Annexes....59

1. PROTOCOL SYNOPSIS FOR STUDY FLR201614

Rationale

This study is a Phase IV, open-label, single-arm study to assess the safety and the necessity of dose adjustment after switching to FLOLAN™ prepared with the reformulated diluent in Japanese patients with pulmonary arterial hypertension (PAH) who are receiving higher doses of FLOLAN than in other countries.

Objectives/Endpoints

The objectives and endpoints of this study are as follows:

Objectives	Endpoints
**Primary**	
• To evaluate the safety and tolerability of the thermostable formulation of FLOLAN (i.e., FLOLAN prepared with the reformulated diluent) when switched from the existing FLOLAN treatment (i.e., FLOLAN prepared with the currently marketed diluent).	• Incidence and severity of Adverse Events (AEs)<br>• Laboratory parameters<br>• 12-lead ECGs<br>• Vital signs<br>• Oxygen saturation
**Secondary**	
• To evaluate the dose adjustment requirements in patients switching from the existing FLOLAN treatment to the thermostable formulation of FLOLAN.	• The number of events to adjust dose of FLOLAN based on the change from baseline to 3 hours in mean Pulmonary Artery Pressure (mPAP) in a subset of subjects*<br>• Reason for changing the dose of thermostable formulation of FLOLAN
• To evaluate the continued efficacy after switching to the thermostable formulation of FLOLAN.	• Change from baseline to Week4 in NT pro BNP and WHO Functional Class<br>• Changes from baseline up to 24 hours and at Week 4 in Haemodynamic parameters: mean Pulmonary Artery Pressure (mPAP), Pulmonary Vascular resistance (PVR), Cardiac Output (CO),

Objectives	Endpoints
	Right Atrial Pressure (RAP) in a subset of subjects*

* Subjects who consent to undergo right heart catheterisation (RHC) over 24-hour and at week 4.

Overall Design

- Phase IV, open-label, single-arm study
- Study Population: Patients who are receiving FLOLAN for the treatment of PAH

Figure 1 Study Schematic

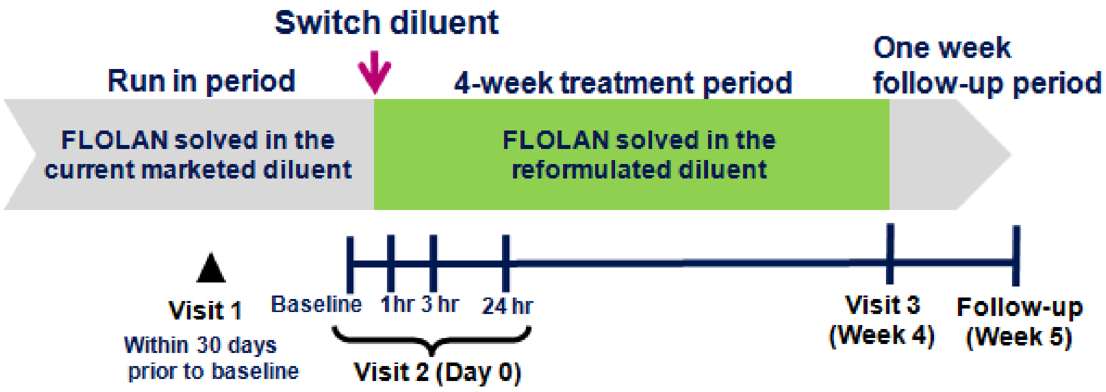

Treatment Arms and Duration

The study will include a screening visit, a run-in period of a maximum of 4 weeks with the existing FLOLAN treatment (i.e., FLOLAN prepared with the currently marketed diluent), a 4-week treatment period with the thermostable formulation of FLOLAN (i.e., FLOLAN prepared with the reformulated diluent) and a one-week follow-up visit.

After the run-in period, subjects undergo baseline assessments at the study centre and switch the study treatment to the thermostable formulation of FLOLAN. Subjects stay overnight in the centre for checking clinical symptoms and haemodynamic stability up to 24 hours after switching the treatment. Subject completion is defined as completion of Week 4.

Period	Description
Run-in	FLOLAN prepared with the currently marketed diluent (epoprostenol sodium+ pH 10.2 – 10.8 diluent) for Injection
Treatment	FLOLAN prepared with the reformulated diluent (epoprostenol sodium + pH 11.7 – 12.3 diluent) for injection

GSK is not providing specific post-study treatment. The post-study care will be decided at the discretion of the investigator (or subinvestigator) based on the patient's condition.

Type and Number of Subjects

Subjects with PAH who have been receiving FLOLAN at 45 ng/kg/min or higher doses will be eligible.

Adequate number of subjects will be enrolled in the study in order to have 10 subjects to complete assessments at 4 weeks, including at least 5 subjects as a subset of subjects who consent to undergo right heart catheterisation (RHC) over 24-hour and at Week 4. Given the nature of the disease the sample size for this study was chosen based on feasibility and no specific hypothesis testing is planned.

Analysis

Data will be summarized using descriptive statistics.

The analysis population will be the Intention-to-Treat (ITT) population consisting of all subjects who have received at least one dose of the thermostable formulation of FLOLAN. The ITT population will be used for all efficacy and safety summaries.

Primary analyses will be frequency tabulation or summary statistics calculation for safety (AEs, clinical laboratory tests [haematology, clinical chemistry and urinalysis], vital signs [systolic and diastolic blood pressure, CO], 12-lead ECG, oxygen saturation) and tolerability on the ITT population.

Secondary analyses will be tabulation of the number of FLOLAN dose adjustment events as well as calculation of summary statistics for the change from baseline for other secondary endpoints.

2. INTRODUCTION

2.1. Study Rationale

This study is a Phase IV, open-label, single-arm study to assess the safety and the necessity of dose adjustment after switching to FLOLAN prepared with the reformulated diluent in Japanese patients with pulmonary arterial hypertension (PAH) who are receiving higher doses of FLOLAN than in other countries.

2.2. Brief Background

FLOLAN is an effective treatment for PAH and reduces mortality; however, safe and effective administration is complex and requires a considerable level of commitment from patients. The currently marketed formulation of FLOLAN requires that the reconstituted drug with diluent specific to FLOLAN should be administered within a 24 hour period and maintained between a temperature of 2 and 8 °C during infusion, necessitating the use of a cold pack. In addition, the cold pack used to maintain the temperature of the reconstituted drug requires frequent changes.

A new formulation of FLOLAN diluent has been developed, which increases the stability of FLOLAN infusion solution at ambient temperatures as the pH of the diluent has been increased from 10.5 (currently marketed diluent, pH 10.2 – 10.8) to 12 (reformulated diluent, pH11.7 – 12.3). Freshly prepared solutions reconstituted and diluted with the reformulated diluent, or solutions that have been stored at 2°C to 8°C (for no longer than 8 days) can be administered up to 24 hours at up to 35°C or up to 72 hours at up to 25°C; it does not, therefore, require the use of a cold pack or frequent changes of the cassette. In this way, it is anticipated that the new formulation will provide an added level of convenience to patients through reduction in the frequency of reconstitution, and elimination of the need for a cold pack, even in countries with relatively high ambient temperatures. The change is limited to the diluent and the active ingredient remains the same as in the current formulation, therefore the new thermostable formulation is not expected to have any impact on the pharmacodynamic actions of FLOLAN and the clinical profile is expected to be the same as that of the current formulation. Actually, the previous overseas study of FLR115322 demonstrated that no dose adjustment was required for switching from the current diluent to the reformulated diluent and similar safety and tolerability of the reformulated diluent in Caucasian PAH patients [Provencher, 2015].

However, there is no experience in Japanese PAH patients for switching to the diluent to be used in this current study. Additionally, relatively higher dosage of FLOLAN is used for Japanese adult patients to manage and inhibit the progression of PAH as compared with those of overseas of 20 – 40 ng/kg/min [Ito, 2012; Galiè, 2009; McLaughlin, 2009]. The post-marketing surveillance in 1999 to 2009 in Japan shows that the longer time the patient has been treated with FLOLAN, the higher doses the patient has been administered in management of PAH. The evidence of the benefit for PAH patients with higher doses of FLOLAN have been reported. Ogawa et al reported that high dose

FLOLAN of mean 79.6 ng/kg/min showed significant long-term survival when compared with dose of mean 54.0 ng/kg/min [Ogawa, 2014]. Also, Akagi et al demonstrated that high dose FLOLAN therapy improved the level of haemodynamic parameters [Akagi, 2010].

This current study will evaluate the safety and tolerability after switching from the current diluent to the reformulated diluent in Japanese PAH patients who have been receiving higher doses of FLOLAN than in other countries.

3. OBJECTIVES AND ENDPOINTS

The objectives and endpoints of this study are as follows:

Objectives	Endpoints
**Primary**	
• To evaluate the safety and tolerability of the new thermostable formulation of FLOLAN (i.e., FLOLAN prepared with the reformulated diluent) when switched from the existing FLOLAN treatment (i.e., FLOLAN prepared with the currently marketed diluent).	• Incidence and severity of Adverse Events (AEs)<br>• Laboratory parameters<br>• 12-lead ECGs<br>• Vital signs<br>• Oxygen saturation
**Secondary**	
• To evaluate the dose adjustment requirements in patients switching from the existing FLOLAN treatment to the thermostable formulation of FLOLAN.	• The number of events to adjust dose of FLOLAN based on the change from baseline to 3 hours in mean Pulmonary Artery Pressure (mPAP) in a subset of subjects<br>• Reason for the change dose of the new thermostable formulation of FLOLAN
• To evaluate the continued efficacy after switching to the thermostable formulation of FLOLAN.	• Change from baseline to Week4 in NT pro BNP and WHO Functional Class<br>• Changes from baseline up to 24 hours and at Week 4 in Haemodynamic parameters: mean Pulmonary Artery Pressure (mPAP), Pulmonary Vascular resistance (PVR), Cardiac Output (CO),

Objectives	Endpoints
	Right Atrial Pressure (RAP) in a subset of subjects*

* Subjects who consent to undergo right heart catheterisation (RHC) over 24-hour and at week 4.

4. STUDY DESIGN

4.1. Overall Design

- Phase IV, open-label, single-arm study
- Study Population: Patients who are receiving FLOLAN for the treatment of PAH

Figure 2 Study Schematic

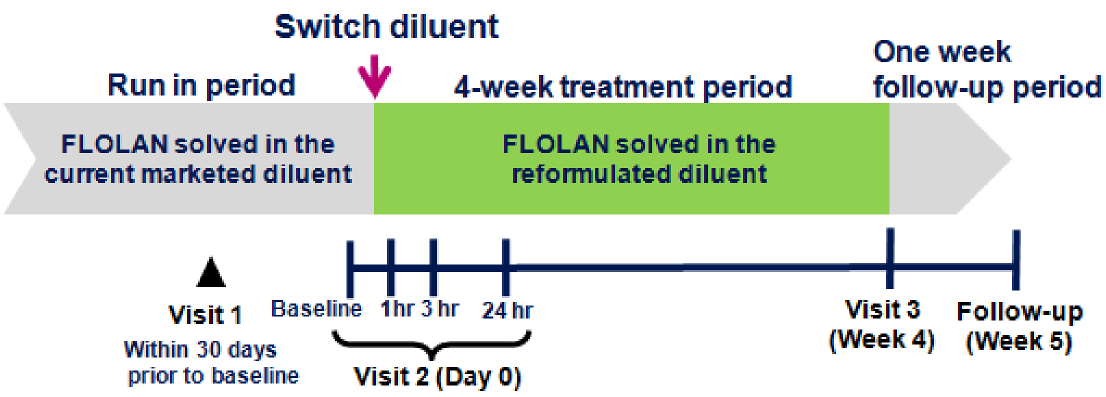

4.2. Treatment Arms and Duration

The study will include a screening visit, a run-in period of a maximum of 4 weeks with the existing FLOLAN treatment (i.e., FLOLAN prepared with the currently marketed diluent), a 4-week treatment period with the thermostable formulation of FLOLAN (i.e., FLOLAN prepared with the reformulated diluent) and a one-week follow-up visit.

After the run-in period, subjects undergo baseline assessments at the study centre and switch the study treatment to the thermostable formulation of FLOLAN. Subjects stay overnight in the centre for checking clinical symptoms and haemodynamic stability up to 24 hours after switching the treatment. Subject completion is defined as completion of Week 4.

Period	Description
Run-in	FLOLAN prepared with the currently marketed diluent (epoprostenol sodium+ pH 10.2 – 10.8 diluent) for Injection
Treatment	FLOLAN prepared with the reformulated diluent (epoprostenol sodium + pH 11.7 – 12.3 diluent) for Injection

GSK is not providing specific post-study treatment. The post-study care will be decided at the discretion of the investigator (or subinvestigator) based on the patient's condition.

4.3. Type and Number of Subjects

Subjects with PAH who are receiving FLOLAN at 45 ng/kg/min or higher doses will be eligible.

Adequate number of subjects will be enrolled in the study in order to have 10 subjects to complete assessments at 4 weeks, including at least 5 subjects as a subset of subjects who consent to undergo right heart catheterisation (RHC) over 24-hour and at Week 4. Given the nature of the disease the sample size for this study was chosen based on feasibility and no specific hypothesis testing is planned.

4.4. Design Justification

PAH is a life threatening disease and FLOLAN is an important treatment for many patients with PAH. The purpose of this study is to assess the safety and the necessity of dose adjustment after switching to FLOLAN prepared with the reformulated diluent in Japanese PAH patients who are receiving higher doses of FLOLAN than in other countries. The primary endpoints are the incidence and severity of AEs, 12-lead ECG, vital signs, and oxygen saturation. The secondary endpoints are NT-proBNP, WHO Functional Class, and haemodynamic parameters to monitor PAH control expected with FLOLAN, a standard PAH treatment in Japan.

As the study also seeks to support that the new formulation of FLOLAN diluent is functionally comparable to the existing formulation, it is important that subjects enrolled are sufficiently stable to allow meaningful comparison between baseline and after treatment. Based on feasibility, an open-label single-arm study has been selected.

The 4-week treatment period was chosen to allow patients sufficient length of time to learn to set up and administer the new thermostable formulation of FLOLAN and to appreciate any meaningful differences the new formulation may bring to their activities of daily living.

4.5. Dose Justification

This section is not applicable as the investigational product (IP) to be assessed by this study is the reformulated diluent for FLOLAN.

4.6. Benefit:Risk Assessment

The pivotal clinical trials evaluating the approved indications and populations for FLOLAN were carried out largely in the 1980's and 1990's. Summaries of findings from both clinical and non-clinical studies conducted with FLOLAN can be found in the Package Insert.

The safety profile of FLOLAN is supported by more than 30 years of post-marketing experience (international birthdate 18 March 1981). Post-marketing safety information is described within the current Package Insert. It is important to note that only the diluent for FLOLAN will change in this study, the vials containing the FLOLAN will not change either during this study or following approval of the reformulated diluent. FLOLAN has a number of risks associated with both the pharmacologic activity of FLOLAN, as well as the administration system. The safety issues described are known risks that are not likely to adversely affect the overall benefit:risk assessment for the use of FLOLAN in PAH (WHO Group 1). The following section outlines the risk assessment and mitigation strategy for this protocol:

4.6.1. Risk Assessment

Potential Risk of Clinical Significance	Summary of Data/Rationale for Risk	Mitigation Strategy
**FLOLAN**		
Medication errors*	FLOLAN preparation and administration is complex, highly individualized, and continuous, all of which increase the risk of medication errors. Reconstitution of FLOLAN with the current sterile diluent for FLOLAN followed by subsequent prolonged refrigeration or infusion over 24 hours without use of a cold pouch could result in reduced efficacy including possible rebound of PAH symptoms due to decreased room temperature stability of FLOLAN when mixed with the pH 10.5 diluent.	GSK will provide the pH 12 diluent. Patients recruited to this study must be on stable and relatively high doses of FLOLAN. Consequently, patients should have a central line in place, be aware of the routine care required for a central line and aware of the risks of FLOLAN therapy.
Local infusion site reactions during long-term infusion**	Because of the high pH of the final FLOLAN infusion solutions, care should be taken to avoid extravasation during administration and consequent risk of tissue damage. The infusion rate of FLOLAN is extremely low when compared with blood flow and the buffer capacity of the FLOLAN solution for infusion is not changed when reconstituted with the reformulated pH 12 diluent for FLOLAN, therefore it is anticipated that the solution will drop to physiologic pH upon mixing with the blood. In addition, neither the in vitro haemolysis study, the in vivo dog study or the small clinical trial in patients who received	Injection sites will be checked at each clinic visit for any reactions.

Potential Risk of Clinical Significance	Summary of Data/Rationale for Risk	Mitigation Strategy
	FLOLAN prepared with pH 12 diluent identified concerns with regards to local tolerance.	
Sepsis, septicaemia	Patients should aware that FLOLAN therapy may require prolonged periods and is complex (e.g., sterile drug reconstitution and permanent indwelling of a central venous catheter).	The inclusion criteria specify that only patients who have been on FLOLAN therapy for PAH are eligible for this study.
Pulmonary oedema	Some patients with PAH have developed pulmonary oedema during dose-ranging (dose initiation), which may be associated with pulmonary veno-occlusive disease. If pulmonary oedema occurs, the infusion of FLOLAN should be discontinued.	The inclusion criteria specify that only patients who have been on FLOLAN therapy for PAH are eligible for this study.<br>The withdrawal criteria specify that treatment should be discontinued if it is not in the best interest of the patient to participate in this study due to the onset of an AE.<br>Vital signs will be monitored continuously by measurement at each clinic visit.
Bleeding events at various sites	FLOLAN is a potent inhibitor of platelet aggregation, therefore, an increased risk for haemorrhagic complications should be considered, particularly for patients with other risk factors for bleeding.	The inclusion criteria specify that only patients who have been on FLOLAN therapy for PAH are eligible for this study.<br>Vital signs will be monitored continuously by measurement at each clinic visit.
Tachycardia	FLOLAN is a potent pulmonary and systemic	Vital signs will be monitored continuously by

Potential Risk of Clinical Significance	Summary of Data/Rationale for Risk	Mitigation Strategy
Bradycardia	vasodilator. The cardiovascular effects during infusion disappear within 30 min of the end of administration. FLOLAN may either decrease or increase heart rate. Blood pressure and heart rate should be monitored during administration of FLOLAN.	measurement at each clinic visit.

*This safety concern may be more likely to occur during the transition from pH 10.5 diluent to pH 12 diluent.
**This safety concern may be more likely to occur with use of FLOLAN solution prepared with pH12 diluent.

4.6.2. Benefit Assessment

Chronic continuous FLOLAN infusion is an effective treatment for PAH (group 1) patients in NYHA Functional Classes III and IV.

This effectiveness has been demonstrated by significant increases in exercise capacity (idiopathic, heritable, and PAH associated with connective tissue diseases) and in survival (idiopathic and heritable PAH) in patients treated with FLOLAN chronically.

Overall, the complexities involved with maintaining the infusion pump and catheter, and reconstituting FLOLAN do not negate the symptomatic benefits of chronic FLOLAN treatment. Use of the reformulated diluent will eliminate the requirement for use of a cold pack, and potentially decrease both the frequency of reconstitution and medication cassette exchanges, thereby potentially slightly simplifying FLOLAN administration.

4.6.3. Overall Benefit:Risk Conclusion

Subjects recruited to this trial are patients with PAH who are on stable and relatively high doses of FLOLAN, and therefore are those patients for whom FLOLAN is effective. In view of the patient population identified for enrollment, GSK assesses that the benefits and risks balance of FLOLAN therapy, including in the context of this study, for the treatment of PAH (Group 1) is favourable.

5. SELECTION OF STUDY POPULATION AND WITHDRAWAL CRITERIA

Specific information regarding warnings, precautions, contraindications, AEs, and other pertinent information on the GSK investigational product or other study treatment that may impact subject eligibility is provided in the product label.

Deviations from inclusion and exclusion criteria are not allowed because they can potentially jeopardize the scientific integrity of the study, regulatory acceptability or subject safety. Therefore, adherence to the criteria as specified in the protocol is essential.

5.1. Inclusion Criteria

A subject will be eligible for inclusion in this study only if all of the following criteria apply:

1. Male or female subjects at least 18 to 75 years at the time of screening;

2. Subjects must be on FLOLAN therapy for pulmonary arterial hypertension (PAH) as approved in the product label;

3. Subjects must receive FLOLAN at 45 ng/kg/min or higher;

4. Subjects must be on stable doses of their existing FLOLAN treatment for a minimum of one month prior to screening; it is acceptable to adjust within 10% of dose during the last one month period;

5. Subjects must be on stable doses of any current PAH treatments other than FLOLAN in the last 30 days prior to screening;

6. Subjects who meet any of the following:

   1) A female subject is eligible to participate if she is not pregnant (as confirmed by a negative [serum or urine] human chorionic gonadotrophin (hCG) test), not lactating, and at least one of the following conditions applies:

   a) Non-reproductive potential defined as:

      - Pre-menopausal females with any of the following: documented tubal ligation, documented hysteroscopic tubal occlusion procedure with follow-up confirmation of bilateral tubal occlusion, hysterectomy, or documented bilateral oophorectomy.

      - Postmenopausal defined as 12 months of spontaneous amenorrhea [in questionable cases a blood sample with simultaneous follicle stimulating hormone (FSH) and estradiol levels consistent with menopause (refer to laboratory reference ranges for confirmatory levels)]. Females on hormone replacement therapy (HRT) and whose menopausal status is in doubt will be required to use one of the highly effective contraception methods if they wish to continue their HRT during the study. Otherwise, they must discontinue HRT to allow confirmation of post-menopausal status prior to study enrolment.

   b) Reproductive potential and agrees to follow one of the options listed below in the GSK Modified List of Highly Effective Methods for Avoiding Pregnancy in Females of Reproductive Potential (FRP) from 30 days prior to the first dose of thermostable formulation of FLOLAN until completion of the follow-up visit.

GSK Modified List of Highly Effective Methods for Avoiding Pregnancy in Females of Reproductive Potential (FRP)

This list does not apply to FRP with same sex partners, when this is their preferred and usual lifestyle or for subjects who are and will continue to be abstinent from penile-vaginal intercourse on a long term and persistent basis.

- Contraceptive subdermal implant that meets the GSK effectiveness criteria including a <1% rate of failure per year, as stated in the product label

- Intrauterine device or intrauterine system that meets the GSK effectiveness criteria including a <1% rate of failure per year, as stated in the product label [Hatcher, 2007a]
- Oral contraceptive, either combined or progestogen alone [Hatcher, 2007a]
- Injectable progestogen [Hatcher, 2007a]
- Contraceptive vaginal ring [Hatcher, 2007a]
- Percutaneous contraceptive patches [Hatcher, 2007a]
- Male partner sterilization with documentation of azoospermia prior to the female subject's entry into the study, and this male is the sole partner for that subject [Hatcher, 2007a]. [Male infertility information will be obtained from a review of the subject's medical records by site staff, subject's medical examinations and/or semen analysis, or an interview with the subject about medical history.]
- Male condom combined with a vaginal spermicide (foam, gel, film, cream, or suppository) [Hatcher, 2007b]

These allowed methods of contraception are only effective when used consistently, correctly and in accordance with the product label. The investigator (or subinvestigator) is responsible for ensuring that subjects understand how to properly use these methods of contraception.

7. Subject must agree not to participate in a clinical study involving another investigational drug or device throughout this study;
8. Subjects must be competent to understand the information given in the Institutional Review Board (IRB) or Independent Ethics Committee (IEC) approved informed consent form and must sign the form prior to the initiation of any study procedures.

5.2. Exclusion Criteria

A subject will not be eligible for inclusion in this study if any of the following criteria apply:

1. Subjects who are given FLOLAN for a condition or in a manner that is outside the approved indication.
2. Subjects with congestive heart failure arising from severe left ventricular dysfunction.
3. Subjects, with or without supplemental oxygen, who have a resting arterial oxygen saturation (SaO2) <90% as measured by pulse oximetry at screening.

4. Subjects have been hospitalised as an emergency or visited the emergency room for a condition related to PAH or treatment for PAH in the last 3 months.
5. The subject's clinical condition is such that they are not expected to remain clinically stable for the duration of the study.
6. Female subjects who are pregnant or breastfeeding.
7. Subjects who have demonstrated noncompliance with previous medical regimens.
8. Subjects who have a history of abusing alcohol or illicit drugs within 1 year.
9. Subjects who have participated in a clinical study involving another investigational drug or device within four weeks before screening.
10. Any concurrent condition that would affect the safety of the subject or in the opinion of the investigator (or subinvestigator) it is not in the best interest of the patient to participate in the study.

5.3. Screening/Baseline/Run-in Failures

Screen failures are defined as subjects who consent to participate in the clinical trial but never received treatment with the thermostable formulation of FLOLAN.

In order to ensure transparent reporting of screen failure subjects, meet the Consolidated Standards of Reporting Trials (CONSORT) publishing requirements, and respond to queries from Regulatory authorities, a minimal set of screen failure information is required including Demography, Screen Failure details, Eligibility Criteria, and Serious Adverse Events.

5.4. Withdrawal/Stopping Criteria

Subjects who do not tolerate treatment will be withdrawn from the study. Treatment for subjects withdrawn from the study will be implemented at the investigator's (or subinvestigator's) discretion.

A subject may also be discontinued prior to completion of the study for the following reasons, but not limited to:

- Adverse event (AE) or serious adverse event (SAE) which in the opinion of the investigator (or subinvestigator) requires withdrawal
- Termination of study by Sponsor
- Lost to follow-up

- Investigator (or subinvestigator)'s discretion
- Consent withdrawn
- Completion of study at study centre

The investigator (or subinvestigator) must take the following actions in relation to a subject who fails to attend the clinic for a required study visit:

- The investigator (or subinvestigator) must attempt to contact the subject and re-schedule the missed visit as soon as possible.
- The investigator (or subinvestigator) must counsel the subject on the importance of maintaining the assigned visit schedule and ascertain whether or not the subject wishes to and/or should continue in the study.
- In cases where the subject is deemed 'lost to follow up', the investigator (or subinvestigator) or designee must make every effort to regain contact with the subject (where possible, 3 telephone calls and if necessary a certified letter to the subject's last known mailing address or local equivalent methods). These contact attempts should be documented in the subject's medical record.
- Should the subject continue to be unreachable, only then will he/she be considered to have withdrawn from the study with a primary reason of "Lost to Follow-up".

A subject may withdraw from study treatment at any time at his/her own request, or may be withdrawn at any time at the discretion of the investigator (or subinvestigator) for safety, behavioural or administrative reasons. If a subject withdraws from the study, he/she may request destruction of any samples taken, and the investigator (or subinvestigator) must document this in the site study records.

5.4.1. Liver Chemistry Stopping Criteria

Liver chemistry stopping criteria are not established to prevent aggravation of PAH due to the discontinuation of FLOLAN and to ensure the safety of subjects of this study.

Phase III-IV liver chemistry increased monitoring criteria have been designed to assure subject safety and evaluate liver event etiology (in alignment with the FDA premarketing clinical liver safety guidance).

Required actions and follow-up assessments after liver stopping or monitoring event are given in Appendix 2.

5.5. Subject and Study Completion

A completed subject is one who has completed all phases of the study including the follow-up visit. The end of the study is defined as the last subject's last visit.

6. STUDY TREATMENT

6.1. Investigational Product

The term 'investigational product (IP)' is used through the protocol to describe the reformulated diluent for FLOLAN.

Product name:	FLOLAN for injection 0.5 mg	FLOLAN for injection 1.5 mg	Reformulated diluent for FLOLAN:<br>(Investigational Product: IP)
Ingredient*/content (as epoprostenol)	0.531mg (0.5 mg)	1.593 mg (1.5 mg)	One vial contains glycine (94 mg), sodium chloride, and sodium hydroxide (pH adjusters).
Excipients (quantity per vial)	D-mannitol (50 mg), glycine (3.76 g), sodium chloride, and sodium hydroxide (pH adjusters)		Glycine (94 mg), sodium chloride, and sodium hydroxide (pH adjusters)
Physical description:	White lyophilised powder or lump powder for injection		
	The pH, osmotic and pressure ratio of the resultant solution when one vial of FLOLAN (0.5 mg, 1.5 mg) is dissolved in 50 mL of the diluent for FLOLAN and the description of the resultant solution when one vial of FLOLAN (0.5 mg, 1.5 mg) is dissolved in 10 mL of the diluent for FLOLAN are as follows:<br><br>pH, 11.7 to 12.3; osmotic pressure ratio, 0.3 to 0.5; description, colorless and clear		

* Epoprostenol sodium

GSK will provide the reformulated diluent for FLOLAN during the study. GSK will not provide FLOLAN, i.e. epoprostenol 0.5 and 1.5 mg lyophile.

6.2. Treatment Assignment

This will be a single-arm open-label study and there will be no treatment assignment.

6.3. Subject Specific Dose Adjustment Criteria

Subjects are monitored regularly to adjust the infusion rate according to symptoms. If required, the dose should be increased or decreased by 1 to 2 ng/kg/min at intervals of at least 15 minutes while monitoring the patient's condition (e.g., symptoms, blood pressure, heart rate, and haemodynamics).

Adverse drug reactions (ADRs) noticed during the administration of FLOLAN (see the product label) are important parameters to decide the optimal rate of infusion. The optimal rate of infusion should be decided through adequate monitoring for ADRs.

6.4. Blinding

This will be a single-arm open-label study and there will be no blinding.

6.5. Packaging and Labeling

A description of packaging and labeling of the investigational product is detailed in a document describing the handling and management of investigational product provided in the Study Reference Manual (SRM).

The contents of the label will be in accordance with all applicable regulatory requirements.

6.6. Preparation/Handling/Storage/Accountability

To prepare the new thermostable product, patients will reconstitute and dilute one or more vials of FLOLAN lyophile (according to therapeutic need) with two vials of the new sterile glycine diluent, giving 100mL of medication for each day of treatment. This reconstituted infusion is filled into medication cassettes prior to either refrigerated storage or immediate continuous intravenous infusion via a central venous catheter using an ambulatory infusion pump.

- Only subjects enrolled in the study may receive the investigational product (reformulated diluent for FLOLAN) or undergo treatment with FLOLAN prepared with the investigational product (study treatment) and only authorised site staff may supply the investigational product or administer the study treatment. All investigational product supplies must be stored in a secure environmentally controlled and monitored (manual or automated) area in accordance with the labelled storage conditions with access limited to the investigator (or subinvestigator) and authorised site staff.

- The investigator (or subinvestigator), institution, or the head of the medical institution (where applicable) is responsible for investigational product accountability, reconciliation, and record maintenance (i.e. receipt, reconciliation and final disposition records).

- Further guidance and information for final disposition of unused investigational product supplies are provided in the SRM.

- Under normal conditions of handling and administration, the investigational product is not expected to pose significant safety risks to site staff.

- A Material Safety Data Sheet (MSDS)/equivalent document describing occupational hazards and recommended handling precautions either will be provided to the investigator (or subinvestigator), where this is required by local laws, or is available upon request from the institution.

- Precaution will be taken to avoid direct contact with the investigational product. A Material Safety Data Sheet (MSDS)/equivalent document describing occupational hazards and recommended handling precautions will be provided to the investigator (or subinvestigator).

6.7. Compliance with Study Treatment Administration

The first dose of thermostable formulation of FLOLAN will be administered to subjects at the site. Administration will be documented in the source documents and reported in the CRF.

When subjects self-administer the thermostable formulation of FLOLAN at home, compliance with the study treatment will be assessed through querying the subject during the site visits and documented in the source documents and CRF. A record of the numbers of lyophile FLOLAN vials (0.5 mg or 1.5 mg) and new FLOLAN diluents dispensed to and used by each subject must be maintained and reconciled with study treatment and compliance records.

6.8. Treatment of Study Treatment Overdose

FLOLAN injection therapy should be maintained at an optimal rate of infusion while monitoring patients at regular intervals to adjust the infusion rate according to symptoms. In this study, therefore, the definition of overdose is not established.

The product label of FLOLAN for injection 0.5 mg and 1.5 mg describes signs/symptoms of overdosage and corrective treatments as follows:

Signs and Symptoms of overdosage: In general, events seen after overdose of FLOLAN represent exaggerated pharmacological effects of the drug (e.g. hypotension and loss of consciousness).

Corrective treatments: In addition to dose reduction or discontinuation, fluid therapy, administration of catecholamine or atropine sulfate hydrate, or other necessary corrective therapies should be provided. When FLOLAN is discontinued, the infusion rate should be decreased as gradually as possible to avoid PAH symptom aggravation or recurrence.

6.9. Treatment after the End of the Study

Subjects will not receive any additional treatment from GSK after completion of the study because the study is a post-marketing clinical study.

The investigator (or subinvestigator) is responsible for ensuring that consideration has been given to the post-study care of the subject's medical condition, whether or not GSK is providing specific post-study treatment.

6.10. Concomitant Medications and Non-Drug Therapies

All concomitant medications and non-drug therapies used by the subject are recorded in the CRF.

6.10.1. Permitted Medications and Non-Drug Therapies

Subjects taking concomitant PAH specific medications must have stable PAH therapy for at least 1 month prior to screening and the therapy will need to be maintained throughout the duration of treatment period. No changes to concomitant PAH-specific medication are allowed during the treatment period.

Examples of permitted PAH-specific medications, but not limited to:

- Vasodilators such as angiotensin-converting enzymes (ACE) inhibitors and calcium channel blockers
- Cyclic guanosine monophosphate (cGMP)-specific phosphodiesterase type 5 (PDE-5) inhibitors such as sildenafil and tadalafil
- Endothelin receptor antagonist (ERA) such as ambrisentan and bosentan

6.10.2. Prohibited Medications and Non-Drug Therapies

During the study, no other prostanoid therapy or its reformulation will be permitted.

7. STUDY ASSESSMENTS AND PROCEDURES

Protocol waivers or exemptions are not allowed with the exception of immediate safety concerns. Therefore, adherence to the study design requirements, including those specified in the Time and Events Table, are essential and required for study conduct.

This section lists the procedures and parameters of each planned study assessment. The exact timing of each assessment is listed in the Time and Events Table (Section 7.1)

7.1. Time and Events Table

	Run-in Period	Treatment Period								Follow-up visit [1]
	Visit 1	Visit 2				Phone call	Unscheduled visit	Early withdrawal	Visit 3	
Procedure	Screening (up to 30 days prior to Baseline)	Baseline (prior to RHC)	1 (+ 0.5hr)	3 ($\pm$ 0.5hr)	24 ($\pm$ 0.5hr)	Day 3, 7 & Week 2 ($\pm$3 days)	To conduct assessments if necessary		Week 4 ($\pm$ 3 days)	(one weeks + 3 days after Week 4)
Informed consent	X									
Inclusion and exclusion criteria	X									
Demography [2]	X									
Medical history/therapeutic history/family history	X									
Disease history	X									
Physical examination (height and weight)		X						X[3]	X[3]	
Concomitant medication review	X	X				X	X	X	X	X
Haemodynamics (RHC) [4]		X [5]	X	X	X			X	X	

	Run-in Period	Treatment Period								Follow-up visit [1]
	Visit 1	Visit 2				Phone call	Unscheduled visit	Early withdrawal	Visit 3	
WHO Functional Class	X	X						X	X	
NT-pro BNP		X			X			X	X	
AE review	X	X	X	X	X	X	X	X	X	X
SAE review	X[6]	X	X	X	X	X	X	X	X	X
Injection site reactions	X	X			X			X	X	
12-lead ECG	X	X			X			X	X	
Oxygen saturation	X	X	X	X	X			X	X	
Vital signs	X	X	X	X	X			X	X	
Pregnancy test [7]	X	X						X	X	
Laboratory assessments	X	X						X	X	
Reasons for new FLOLAN diluent dose adjustment			X	X	X		X	X	X	
Study Treatment (Assess compliance)						X	X	X	X	

RHC: right heart catheterization
1. Allow phone contact.

2. Birth year, gender, race, ethnicity
3. Weight only
4. Only subjects who have given consent to undergo RHC over 24-hour and at Week4
5. For Baseline, within 0.5 hr prior to switching to the new Flolan diluent
6. Any SAEs assessed as related to study participation or related to a GSK product will be collected from the time a subject consents to participate in the study.
7. Urinary or serum test for women of childbearing potential

7.2. Screening and Critical Baseline Assessments

Screening Visit (Visit 1)

Subjects on a stable dose of the existing FLOLAN therapy will be screened to determine if they meet the eligibility criteria. Subjects who are eligible for the trial will sign an informed consent prior to initiation of any study related procedures.

At the Screening Visit, cardiovascular history/risk factors (to be detailed in the CRF) will be assessed.

Information will be collected on the following demographic characteristics: birth year, gender, race and ethnicity.

Medical, disease, therapeutic and family history will be assessed against the inclusion/exclusion criteria listed in Section 5.

Subjects will enter a run-in period of a maximum of 4 weeks after the Screening visit. Subjects will come back to the hospital for the Baseline Visit within 4 weeks post Screening Visit (Visit 1).

Baseline Visit (Visit 2)

At Baseline Visit (Visit2), subjects will be switched from the existing FLOLAN therapy and will receive FLOLAN prepared with the reformulated diluent for a period of 4 weeks. All baseline procedures will be performed before the switch to the thermostable formulation of FLOLAN. Subjects will remain in the hospital for a minimum of 24 hours of treatment.

All subjects will undergo the following procedure: The switch to the thermostable formulation of FLOLAN will start with an equivalent dose of their existing FLOLAN therapy. If necessary, the investigator (or subinvestigator) will adjust the dose as appropriate for the subject until they are stable on the thermostable formulation of FLOLAN. After switching to the thermostable formulation of FLOLAN, subjects will be kept in the hospital for an observation period of at least 24 hours. During the observation period, subjects will be monitored closely for safety and haemodynamic stability up to 24 hours after the switching. If a subject cannot be stabilized, the dose of FLOLAN for a subject will be adjusted and the subject will stay at the hospital for an adequate period per the discretion of the investigator (or subinvestigator). After that treatment, the subject will be discharged and will stay in the trial. These subjects will follow the telephone call procedures as outlined below and in the Time and Events Table (Section 7.1).

7.3. Efficacy

7.3.1. Haemodynamics

Haemodynamic parameters, i.e., mean pulmonary artery blood pressure [mPAP], pulmonary vascular resistance [PVR], cardiac output [CO], and right atrial pressure

[RAP] will be assessed at baseline and at 1, 3 and 24 hours after the first dose of thermostable formulation of FLOLAN (or at early withdrawal visit if possible).

7.3.2. WHO Functional Class

WHO Functional Class will be assessed at the screening visit, baseline and Week 4 (or at early withdrawal visit). The investigator (or subinvestigator) conducting the assessment at baseline should also be responsible for the assessment at Week 4 wherever possible.

7.3.3. N-terminal Pro B-type Natriuretic Peptide: NT-pro BNP

For measurement of plasma NT-proBNP concentrations, a blood sample will be taken at baseline, 24 hours after the first dose of thermostable formulation of FLOLAN, and Week 4 (or at early withdrawal visit).

7.4. Safety

Planned time points for all safety assessments are listed in the Time and Events Table (Section 7.1). Additional time points for safety tests (such as vital signs, physical exams and laboratory safety tests) may be added during the course of the study based on newly available data to ensure appropriate safety monitoring.

7.4.1. Adverse Events (AE) and Serious Adverse Events (SAEs)

The definitions of an AE or SAE can be found in Appendix 3.

The investigator (or investigator) and their designees are responsible for detecting, documenting and reporting events that meet the definition of an AE or SAE.

7.4.1.1. Time Period and Frequency for Collecting AE and SAE Information

- AEs and SAEs will be collected from the start of treatment with the thermostable formulation of FLOLAN until the follow-up contact (see Section 7.4.1.3), at the timepoints specified in the Time and Events Table (Section 7.1).
- Medical occurrences that begin prior to the start of treatment with the thermostable formulation of FLOLAN but after obtaining informed consent may be recorded on the Medical History/Current Medical Conditions section of the CRF.

- Any SAEs assessed as related to study participation (e.g., protocol-mandated procedures, invasive tests, or change in existing therapy) or related to a GSK product will be recorded from the time a subject consents to participate in the study up to and including any follow-up contact.
- All SAEs will be recorded and reported to GSK within 24 hours, as indicated in Appendix 3.
- Investigators (or subinvestigators) are not obligated to actively seek AEs or SAEs in former study subjects. However, if the investigator (or subinvestigator) learns of any SAE, including a death, at any time after a subject has been discharged from the study, and he/she considers the event reasonably related to the investigational product (reformulated diluent for FLOLAN) or study participation, the investigator (or subinvestigator) must promptly notify GSK.

NOTE: The method of recording, evaluating and assessing causality of AEs and SAEs plus procedures for completing and transmitting SAE reports to GSK are provided in Appendix 3.

7.4.1.2. Method of Detecting AEs and SAEs

Care will be taken not to introduce bias when detecting AEs and/or SAEs. Open-ended and non-leading verbal questioning of the subject is the preferred method to inquire about AE occurrence. Appropriate questions include:

- “How are you feeling?”
- “Have you had any (other) medical problems since your last visit/contact?”
- “Have you taken any new medicines, other than those provided in this study, since your last visit/contact?”

7.4.1.3. Follow-up of AEs and SAEs

After the initial AE/SAE report, the investigator (or subinvestigator) is required to proactively follow each subject at subsequent visits/contacts. All SAEs, and non-serious AEs of special interest (as defined in Section 4.6.1) will be followed until resolution, until the condition stabilizes, until the event is otherwise explained, or until the subject is lost to follow-up (as defined in Section 5.4). Further information on follow-up procedures is given in Appendix 3.

7.4.1.4. Cardiovascular and Death Events

For any cardiovascular events detailed in Appendix 3 and all deaths, whether or not they are considered SAEs, specific Cardiovascular (CV) and Death sections of the CRF will be required to be completed. These sections include questions regarding cardiovascular (including sudden cardiac death) and non-cardiovascular death.

The CV CRFs are presented as queries in response to reporting of certain CV MedDRA terms. The CV information should be recorded in the specific cardiovascular section of the CRF within one week of receipt of a CV Event data query prompting its completion.

The Death CRF is provided immediately after the occurrence or outcome of death is reported. Initial and follow-up reports regarding death must be completed within one week of when the death is reported.

7.4.1.5. Regulatory Reporting Requirements for SAEs

Prompt notification by the investigator (or subinvestigator) to GSK of SAEs related to investigational product (even for non-interventional post-marketing studies) is essential so that legal obligations and ethical responsibilities towards the safety of subjects and the safety of a product under clinical investigation are met.

GSK has a legal responsibility to notify both the local regulatory authority and other regulatory agencies about the safety of a product under clinical investigation. GSK will comply with country specific regulatory requirements relating to safety reporting to the regulatory authority, Institutional Review Board (IRB)/Independent Ethics Committee (IEC) and investigators.

Investigator safety reports are prepared for suspected unexpected serious adverse reactions according to local regulatory requirements and GSK policy and are forwarded to investigators as necessary.

An investigator who receives an investigator safety report describing a SAE(s) or other specific safety information (e.g., summary or listing of SAEs) from GSK will file it with the IB and will notify the IRB/IEC, if appropriate according to local requirements.

7.4.2. Pregnancy

- Details of all pregnancies in female subjects and female partners of male subjects will be collected after the start of treatment with the thermostable formulation of FLOLAN and until delivery (including pregnancies resulting in early termination).

- If a pregnancy is reported then the investigator (or subinvestigator) should inform GSK within 2 weeks of learning of the pregnancy and should follow the procedures outlined in Appendix 4.

7.4.3. Physical Exams

Physical examinations will be conducted at baseline (height and weight) Week 4 (weight only) and, if applicable, early withdrawal visit (weight only).Investigators (or subinvestigators) should pay special attention to clinical signs related to previous serious illnesses.

7.4.4. Vital Signs

Vital signs will be measured in semi-supine position after 5 minutes rest and will include systolic and diastolic blood pressure and pulse rate.

7.4.5. Oxygen Saturation

Oxygen saturation will be measured by pulse oximetry.

7.4.6. Electrocardiogram (ECG)

A 12-lead ECG will be done in a supine position after having kept a subject at rest in this position for about 5 minutes at each assessment time.

7.4.7. Clinical Safety Laboratory Assessments

The following clinical laboratory test parameters will be measured at the institution's local laboratory. Results of each assessment are recorded in the CRF.

Laboratory Assessments	Parameters
Haematology	Haemoglobin, hematocrit, RBC count, WBC count (including fractions), platelet count
Clinical Chemistry [1]	ALT(GPT), AST(GOT), Al-P, γ-GTP, LDH, CPK, total bilirubin, direct bilirubin, creatinine, Na, Mg, K, Cl, inorganic phosphorus, calcium, BUN, uric acid, glucose, total protein, albumin, thyroid function tests (T3, T4, TSH)
Routine Urinalysis (dip stick)	Protein, sugar, occult blood
Pregnancy tests	hCG (only women of childbearing potential)

1. Details of Liver Chemistry Stopping Criteria and Required Actions and Follow-Up Assessments after liver stopping or monitoring event are given in Appendix 2.

If additional non-protocol specified laboratory assessments are performed at the institution's local laboratory and result in a change in subject management or are considered clinically significant by the investigator (or subinvestigator) (e.g., SAE or AE or dose modification) the results must be recorded in the CRF.

7.5. Other Endpoints

7.5.1. Reasons for Dose Adjustment after Switch

A dose adjustment, if required after switching to the reformulated diluent for FLOLAN, will be recorded in the CRF. A dose adjustment, if required in a subject undergoing RHC based on mPAP values measured during the 3 hours after switching, will be recorded in the CRF.

8. DATA MANAGEMENT

- For this study subject data will be collected using GSK defined case report forms and combined with data provided from other sources in a validated data system.
- Management of clinical data will be performed in accordance with applicable GSK standards and data cleaning procedures to ensure the integrity of the data, e.g., removing errors and inconsistencies in the data.
- Adverse events and concomitant medications terms will be coded using MedDRA (Medical Dictionary for Regulatory Activities) and an internal validated medication dictionary, GSKDrug.
- Original CRFs (including queries and audit trails) will be retained by GSK, while the investigator will retain a copy. Subject initials will not be collected or transmitted to GSK according to GSK policy.

9. STATISTICAL CONSIDERATIONS AND DATA ANALYSES

9.1. Hypotheses

Data will be summarized using descriptive statistics. No formal hypothesis tests are planned.

9.2. Sample Size Considerations

9.2.1. Sample Size Assumptions

The sample size for this study is based on the feasibility, not based on hypothesis testing.

9.2.2. Sample Size Sensitivity

The sample size for this study is based on the feasibility. No sample size sensitivity testing has been conducted.

9.2.3. Sample Size Re-estimation or Adjustment

No sample size re-estimation is planned.

9.3. Data Analysis Considerations

9.3.1. Analysis Populations

The Intention-to-Treat (ITT) population will consist of all subjects who received at least one dose of the thermostable formulation of FLOLAN. The ITT population will be used for all efficacy and safety summaries.

9.3.2. Analysis Data Sets

Analysis datasets will consist of all data collected in the study and evaluated according to the analysis populations.

9.3.3. Treatment Comparisons

No formal comparisons are planned for this study.

9.3.4. Interim Analysis

No interim analysis is planned.

9.4. Key Elements of Analysis Plan

The Analysis Plan will consist of summary and graphical displays. Individual patient listings will also be included. Full details will be described in a "Reporting and Analysis Plan". Data will be summarized using a tabular display wherever possible according to the GSK reporting standard.

9.4.1. Primary Analyses

Safety (AEs, clinical laboratory tests [haematology, clinical chemistry and urinalysis], vital signs [systolic and diastolic blood pressure, cardiac output], 12-lead ECG, oxygen saturation) and tolerability will be assessed on all subjects who received at least one dose of the thermostable formulation of FLOLAN. For AEs, the number and percentage of subjects with AE will be presented. For SAEs and AEs resulting in treatment discontinuation, AEs by severity and AEs by causal relationship, the number and percentage of subjects with AE will be presented. All AEs will be coded using the preferred terms (PT) and grouped into system organ classes (SOC) according to the Medical Dictionary for Regulatory Activities (MedDRA).

For clinical laboratory tests, vital signs, oxygen saturation and 12-lead ECG, summary statistics by visit will be calculated. For clinical laboratory tests, the number and percentage of subjects with values outside the normal range of clinical interest will be tabulated by visit. For 12-lead ECG findings, frequency tabulation by visit will be provided.

9.4.2. Secondary Analyses

In order to assess necessity of dose adjustment in subjects after switching to the thermostable formulation of FLOLAN, the frequency of dose adjustment requirements will be tabulated based on the changes from baseline in mPAP up to 3 hours after dosing. Reasons for dose adjustment after switching to the thermostable formulation of FLOLAN will be listed.

In order to assess continued efficacy after switching to the thermostable formulation of FLOLAN, summary statistics will be provided for the change from baseline to Week 4 in NT-proBNP, WHO Functional Class distribution at Week 4, and the changes from baseline up to 24 hours after dosing and at Week 4 for the following haemodynamic parameters: mPAP, PVR, CO and RAP.

9.4.3. Other Analyses

All other data (demography, medical history, disease history, therapeutic history, concomitant medications, exposure, compliance to study treatment, infusion site assessment) will be summarized descriptively.

10. STUDY GOVERNANCE CONSIDERATIONS

10.1. Posting of Information on Publicly Available Clinical Trial Registers

Study information from this protocol will be posted on publicly available clinical trial registers before enrollment of subjects begins.

10.2. Regulatory and Ethical Considerations, Including the Informed Consent Process

Prior to initiation of a site, GSK will obtain favourable opinion/approval from the appropriate regulatory agency to conduct the study in accordance with ICH Good Clinical Practice (GCP) and applicable country-specific regulatory requirements.

The study will be conducted in accordance with all applicable regulatory requirements, and with GSK policy.

The study will also be conducted in accordance with ICH Good Clinical Practice (GCP), all applicable subject privacy requirements, and the guiding principles of the current version of the Declaration of Helsinki. This includes, but is not limited to, the following:

- IRB/IEC review and favorable opinion/approval of the study protocol and amendments as applicable.
- Obtaining signed informed consent from each subject before participating in the study (for amendments as applicable).
- Investigator reporting requirements (e.g. reporting of AEs/SAEs/protocol deviations to IRB/IEC).
- GSK will provide full details of the above procedures, either verbally, in writing, or both.

The procedure for obtaining signed informed consent is as follows:

- Prior to participation in the study, the investigator (or subinvestigator) should fully inform the potential subject of the study (and/or his/her proxy consenter) including the written information. The investigator (or subinvestigator) should provide the subject (and/or his/her proxy consenter) ample time and opportunity to inquire about details of the study. The subject (and/or his/her proxy consenter) should sign and

personally date the consent form. The proxy consenter, if needed by the subject, should confirm the subject's will to participate in the study, and sign and personally date the consent form.

- If the subject wishes to consider the content of the written information at home, he/she may sign the consent form at home. The person who conducted the informed consent discussion and the study collaborator giving supplementary explanation, where applicable, should sign and personally date the consent form.
- If an impartial witness is required, the witness should sign and personally date the consent form.
- The investigator (or subinvestigator) should retain this signed and dated form (and other written information) together with the source medical records, such as clinical charts (in accordance with the rules for records retention, if any, at each medical institution) and give a copy to the subject (and/or his/her proxy consenter).

10.3. Quality Control (Study Monitoring)

- In accordance with applicable regulations including GCP, and GSK procedures, GSK monitors will contact the site prior to the start of the study to review with the site staff the protocol, study requirements, and their responsibilities to satisfy regulatory, ethical, and GSK requirements.
- When reviewing data collection procedures, the discussion will also include identification, agreement and documentation of data items for which the CRF will serve as the source document.

GSK will monitor the study and site activity to verify that the:

- Data are authentic, accurate, and complete.
- Safety and rights of subjects are being protected.
- Study is conducted in accordance with the currently approved protocol and any other study agreements, GCP, and all applicable regulatory requirements.

The investigator and the head of the medical institution (where applicable) agrees to allow the monitor direct access to all relevant documents.

10.4. Quality Assurance

- To ensure compliance with GCP and all applicable regulatory requirements, GSK may conduct a quality assurance assessment and/or audit of the site records, and the

regulatory agencies may conduct a regulatory inspection at any time during or after completion of the study.

- In the event of an assessment, audit or inspection, the investigator (and the head of the medical institution) must agree to grant the advisor(s), auditor(s) and inspector(s) direct access to all relevant documents and to allocate their time and the time of their staff to discuss the conduct of the study, any findings/relevant issues and to implement any corrective and/or preventative actions to address any findings/issues identified.

10.5. Study and Site Closure

- Upon completion or premature discontinuation of the study, the GSK monitor will conduct site closure activities with the investigator or site staff, as appropriate, in accordance with applicable regulations including GCP, and GSK Standard Operating Procedures.
- GSK reserves the right to temporarily suspend or prematurely discontinue this study at any time for reasons including, but not limited to, safety or ethical issues or severe non-compliance. For multicentre studies, this can occur at one or more or at all sites.
- If GSK determines such action is needed, GSK will discuss the reasons for taking such action with the investigator or the head of the medical institution (where applicable). When feasible, GSK will provide advance notification to the investigator or the head of the medical institution, where applicable, of the impending action.
- If the study is suspended or prematurely discontinued for safety reasons, GSK will promptly inform all investigators, heads of the medical institutions (where applicable) and/or institution(s) conducting the study. GSK will also promptly inform the relevant regulatory authorities of the suspension or premature discontinuation of the study and the reason(s) for the action.
- If required by applicable regulations, the investigator or the head of the medical institution (where applicable) must inform the IRB/IEC promptly and provide the reason for the suspension or premature discontinuation.

10.6. Records Retention

- Following closure of the study, the investigator or the head of the medical institution (where applicable) must maintain all site study records (except for those required by local regulations to be maintained elsewhere), in a safe and secure location.
- The records must be maintained to allow easy and timely retrieval, when needed (e.g., for a GSK audit or regulatory inspection) and must be available for review in

conjunction with assessment of the facility, supporting systems, and relevant site staff.

- Where permitted by local laws/regulations or institutional policy, some or all of these records can be maintained in a format other than hard copy (e.g., microfiche, scanned, electronic); however, caution needs to be exercised before such action is taken.

- The investigator must ensure that all reproductions are legible and are a true and accurate copy of the original and meet accessibility and retrieval standards, including re-generating a hard copy, if required. Furthermore, the investigator must ensure there is an acceptable back-up of these reproductions and that an acceptable quality control process exists for making these reproductions.

- GSK will inform the investigator of the time period for retaining these records to comply with all applicable regulatory requirements. The minimum retention time will meet the strictest standard applicable to that site for the study, as dictated by any institutional requirements or local laws or regulations, GSK standards/procedures, and/or institutional requirements.

- The investigator must notify GSK of any changes in the archival arrangements, including, but not limited to, archival at an off-site facility or transfer of ownership of the records in the event the investigator is no longer associated with the site.

10.7. Provision of Study Results to Investigators, Posting of Information on Publically Available Clinical Trials Registers and Publication

Where required by applicable regulatory requirements, an investigator signatory will be identified for the approval of the clinical study report. The investigator will be provided reasonable access to statistical tables, figures, and relevant reports and will have the opportunity to review the complete study results at a GSK site or other mutually-agreeable location.

GSK will also provide the investigator with the full summary of the study results. The investigator is encouraged to share the summary results with the study subjects, as appropriate.

The procedures and timing for public disclosure of the results summary and for development of a manuscript for publication will be in accordance with GSK Policy.

11. REFERENCES

Akagi S, Nakamura K, Miyaji K, et al. Marked haemodynamic improvements by high-dose epoprostenol therapy in patients with idiopathic pulmonary arterial hypertension. Circ J. 2010 Oct;74(10): 2200-5.

Galiè N, Hoeper MM, Humbert M, et al. Guidelines for the diagnosis and treatment of pulmonary hypertension: the Task Force for the Diagnosis and Treatment of Pulmonary Hypertension of the European Society of Cardiology (ESC) and the European Respiratory Society (ERS), endorsed by the International Society of Heart and Lung Transplantation (ISHLT). Eur Heart J. 2009 Oct;30(20): 2493-537.

Hatcher RA, Trussell J, Nelson AL, Cates W Jr, Stewart F, Kowal D, editors. Contraceptive Technology. 19th edition. New York: Ardent Media, 2007(a): 24. Table 3-2

Hatcher RA, Trussell J, Nelson AL, Cates W Jr, Stewart F, Kowal D, editors. Contraceptive Technology. 19th edition. New York: Ardent Media, 2007(b): 28

Ito H, Matsubara H. Pulmonary Arterial Hypertension Treatment Manual: Latest Treatment Guidance Aiming at Complete Cure. Nankodo Co.,Ltd. Mar. 2012

McLaughlin VV, Archer SL, Badesch DB, et al. ACCF/AHA 2009 expert consensus document on pulmonary hypertension a report of the American College of Cardiology Foundation Task Force on Expert Consensus Documents and the American Heart Association developed in collaboration with the American College of Chest Physicians; American Thoracic Society, Inc.; and the Pulmonary Hypertension Association. J Am Coll Cardiol. 2009 Apr 28;53(17): 1573-619.

Ogawa A, Ejiri K, Matsubara H. Long-term patient survival with idiopathic/heritable pulmonary arterial hypertension treated at a single centre in Japan. Life Sci. 2014 Nov 24;118(2): 414-9.

Provencher S, Paruchuru P, Spezzi A, et al. Quality of life, safety and efficacy profile of thermostable flolan in pulmonary arterial hypertension. PLoS One. 2015 Mar 20;10(3): e0120657.

12. APPENDICES

12.1. Appendix 1 – Abbreviations and Trademarks

Abbreviations

ACCP	American College of Chest Physician
AE	Adverse Event
AL-P	Alkaline Phosphatase
ALT	Alanine Aminotransferase
AST	Aspartate Aminotransferase
BUN	Blood Urea Nitrogen
CI	Cardiac Index
CPK	Creatine Phosphokinase
CRF	Case Report Form
ECG	Electrocardiogram
FDA	Food and Drug Administration
GCP	Good Clinical Practice
GSK	GlaxoSmithKline
γ-GTP	γ-Glutamyltranspeptidase
hCG	Human Chorionic Gonadotropin
ICF	Informed Consent Form
IEC	Independent Ethics Committee
IRB	Institutional Review Board
LDH	Lactate Dehydrogenase
mPAP	Mean Pulmonary Artery Pressure
NT-pro-BNP	N-Terminal pro-B-type Natriuretic Peptide
PAH	Pulmonary Arterial Hypertension
PAP	Pulmonary Artery Pressure
PDE	Phosphodiesterase
PPH	Primary Pulmonary Hypertension
PDE-5	Phosphodiesterase type 5
PGI_2	Prostacyclin
PVR	Pulmonary Vascular Resistance
RAP	Right Atrial Pressure
RHC	Right Heart Catheterization
SAE	Serious Adverse Event
SaO_2	Arterial Oxygen Saturation
T3	Triiodothyronine
T4	Thyroxine
TSH	Thyroid Stimulating Hormone
WHO	World Health Organization

Trademark Information

Trademarks of the GlaxoSmithKline group of companies
FLOLAN

Trademarks not owned by the GlaxoSmithKline group of companies
NONE

12.2. Appendix 2: Liver Safety Required Actions and Follow up Assessments

Phase III-IV liver chemistry stopping and increased monitoring criteria have been designed to assure subject safety and evaluate liver event etiology (in alignment with the FDA premarketing clinical liver safety guidance). http://www.fda.gov/downloads/Drugs/GuidanceComplianceRegulatoryInformation/Guidances/UCM174090.pdf

Phase III-IV liver chemistry increased monitoring criteria with continued therapy

Liver Chemistry Increased Monitoring Criteria – Liver Event	
**Criteria**	**Actions**
ALT ≥3xULN with or without bilirubin ≥2xULN[1,2] and with or without symptoms believed to be related to liver injury or hypersensitivity	• Subject can continue study treatment at the discretion of the investigator (subinvestigator)<br>• Report the event to GSK **within 24 hours**<br>• Complete the liver event CRF and complete an SAE data collection tool if the event also meets the criteria for an SAE[2]<br>• Perform liver event follow up assessments at discretion of the investigator (subinvestigator)<br>◦ Viral hepatitis serology[4]<br>◦ Only in those with underlying chronic hepatitis B at study entry (identified by positive hepatitis B surface antigen) quantitative hepatitis B DNA and hepatitis delta antibody[5].<br>◦ Serum creatine phosphokinase (CPK) and lactate dehydrogenase (LDH).<br>◦ Fractionate bilirubin, if total bilirubin≥2xULN<br>◦ Obtain complete blood count with differential to assess eosinophilia<br>◦ For bilirubin ≥2xULN: Anti-nuclear antibody, anti-smooth muscle antibody, Type 1 anti-liver kidney microsomal antibodies, and quantitative total immunoglobulin G (IgG or gamma globulins), serum acetaminophen adduct HPLC assay (quantifies potential acetaminophen contribution to liver injury

	in subjects with definite or likely acetaminophen use in the preceding week [James, 2009]) and liver imaging (ultrasound, magnetic resonance, or computerised tomography) and /or liver biopsy to evaluate liver disease; complete Liver Imaging and/or Liver Biopsy CRF forms.<br>• Repeat liver chemistries (include ALT, AST, alkaline phosphatase, bilirubin) and perform liver event follow up assessments within 24 hrs if bilirubin ≥2xULN and within 72 hrs if bilirubin <2xULN<br>• Subject must return at least weekly for repeat liver chemistries (ALT, AST, alkaline phosphatase, bilirubin) until they resolve, stabilise or return to within baseline

1. Serum bilirubin fractionation should be performed if testing is available. If serum bilirubin fractionation testing is unavailable, **record presence of detectable urinary bilirubin on dipstick**, indicating direct bilirubin elevations and suggesting liver injury.
2. All events of ALT ≥ 3xULN **and** bilirubin ≥ 2xULN (>35% direct bilirubin) or ALT ≥ 3xULN **and** INR>1.5, if INR measured which may indicate severe liver injury (possible 'Hy's Law'), **must be reported as an SAE (excluding studies of hepatic impairment or cirrhosis)**; INR measurement is not required and the threshold value stated will not apply to subjects receiving anticoagulants
3. New or worsening symptoms believed to be related to liver injury (such as fatigue, nausea, vomiting, right upper quadrant pain or tenderness, or jaundice) or believed to be related to hypersensitivity (such as fever, rash or eosinophilia)
4. Includes: Hepatitis A IgM antibody; Hepatitis B surface antigen and Hepatitis B Core Antibody (IgM); Hepatitis C RNA; Cytomegalovirus IgM antibody; Epstein-Barr viral capsid antigen IgM antibody (or if unavailable, obtain heterophile antibody or monospot testing); Hepatitis E IgM antibody
5. If hepatitis delta antibody assay cannot be performed,, it can be replaced with a PCR of hepatitis D RNA virus (where needed) [Le Gal, 2005].

References

James LP, Letzig L, Simpson PM, Capparelli E, Roberts DW, Hinson JA, Davern TJ, Lee WM. Pharmacokinetics of Acetaminophen-Adduct in Adults with Acetaminophen Overdose and Acute Liver Failure. Drug Metab Dispos 2009; 37:1779-1784.

Le Gal F, Gordien E, Affolabi D, Hanslik T, Alloui C, Dény P, Gault E. Quantification of Hepatitis Delta Virus RNA in Serum by Consensus Real-Time PCR Indicates Different Patterns of Virological Response to Interferon Therapy in Chronically Infected Patients. J Clin Microbiol. 2005;43(5):2363–2369.

12.3. Appendix 3: Definition of and Procedures for Recording, Evaluating, Follow-Up and Reporting of Adverse Events

12.3.1. Definition of Adverse Events

**Adverse Event Definition:**
• An AE is any untoward medical occurrence in a patient or clinical investigation subject, temporally associated with the use of a medicinal product, whether or not considered related to the medicinal product.<br>• NOTE: An AE can therefore be any unfavorable and unintended sign (including an abnormal laboratory finding), symptom, or disease (new or exacerbated) temporally associated with the use of a medicinal product.

**Events <u>meeting</u> AE definition include:**
• Any abnormal laboratory test results (hematology, clinical chemistry, or urinalysis) or other safety assessments (e.g., ECGs, radiological scans, vital signs measurements), including those that worsen from baseline, and felt to be clinically significant in the medical and scientific judgement of the investigator (subinvestigator).<br>• Exacerbation of a chronic or intermittent pre-existing condition including either an increase in frequency and/or intensity of the condition.<br>• New conditions detected or diagnosed after study treatment administration even though it may have been present prior to the start of the study.<br>• Signs, symptoms, or the clinical sequelae of a suspected interaction.<br>• Signs, symptoms, or the clinical sequelae of a suspected overdose of either study treatment or a concomitant medication (overdose per se will not be reported as an AE/SAE unless this is an intentional overdose taken with possible suicidal/self-harming intent. This should be reported regardless of sequelae).<br>• "Lack of efficacy" or "failure of expected pharmacological action" per se will not be reported as an AE or SAE. However, the signs and symptoms and/or clinical sequelae resulting from lack of efficacy will be reported if they fulfil the definition of an AE or SAE.

**Events NOT meeting definition of an AE include:**
• Any clinically significant abnormal laboratory findings or other abnormal safety assessments which are associated with the underlying disease, unless judged by the investigator (subinvestigator) to be more severe than expected for the subject's condition.<br><br>• The disease/disorder being studied or expected progression, signs, or symptoms of the disease/disorder being studied, unless more severe than expected for the subject's condition.<br><br>• Medical or surgical procedure (e.g., endoscopy, appendectomy): the condition that leads to the procedure is an AE.<br><br>• Situations where an untoward medical occurrence did not occur (social and/or convenience admission to a hospital).<br><br>• Anticipated day-to-day fluctuations of pre-existing disease(s) or condition(s) present or detected at the start of the study that do not worsen.

12.3.2. Definition of Serious Adverse Events

If an event is not an AE per definition above, then it cannot be an SAE even if serious conditions are met (e.g., hospitalization for signs/symptoms of the disease under study, death due to progression of disease, etc).

**Serious Adverse Event (SAE) is defined as any untoward medical occurrence that, at any dose:**
**a. Results in death**
**b. Is life-threatening**<br><br>NOTE:<br><br>The term 'life-threatening' in the definition of 'serious' refers to an event in which the subject was at risk of death at the time of the event. It does not refer to an event, which hypothetically might have caused death, if it were more severe.
**c. Requires hospitalization or prolongation of existing hospitalization**<br><br>NOTE:<br><br>• · In general, hospitalization signifies that the subject has been detained (usually involving at least an overnight stay) at the hospital or emergency ward for observation and/or treatment that would not have been appropriate in the physician's office or out patient setting. Complications that occur during hospitalization are

	AEs. If a complication prolongs hospitalization or fulfills any other serious criteria, the event is serious. When in doubt as to whether "hospitalization" occurred or was necessary, the AE should be considered serious.
•	· Hospitalization for elective treatment of a pre-existing condition that did not worsen from baseline is not considered an AE.
**d.**	**Results in disability/incapacity**
	NOTE:
•	The term disability means a substantial disruption of a person's ability to conduct normal life functions.
•	This definition is not intended to include experiences of relatively minor medical significance such as uncomplicated headache, nausea, vomiting, diarrhea, influenza, and accidental trauma (e.g. sprained ankle) which may interfere or prevent everyday life functions but do not constitute a substantial disruption.
**e.**	**Is a congenital anomaly/birth defect**
**f.**	**Other situations:**
•	Medical or scientific judgment should be exercised in deciding whether reporting is appropriate in other situations, such as important medical events that may not be immediately life-threatening or result in death or hospitalization but may jeopardize the subject or may require medical or surgical intervention to prevent one of the other outcomes listed in the above definition. These should also be considered serious.
•	Examples of such events are invasive or malignant cancers, intensive treatment in an emergency room or at home for allergic bronchospasm, blood dyscrasias or convulsions that do not result in hospitalization, or development of drug dependency or drug abuse.
**g.**	**Is associated with liver injury and impaired liver function defined as:**
•	ALT ≥ 3xULN and total bilirubin* ≥ 2xULN (>35% direct), **or**
•	ALT ≥ 3xULN and INR** > 1.5.
	* Serum bilirubin fractionation should be performed if testing is available; if unavailable, measure urinary bilirubin via dipstick. If fractionation is unavailable and ALT ≥ 3xULN and total bilirubin ≥ 2xULN, then the event is still to be reported as an SAE.
	** INR testing not required per protocol and the threshold value does not apply to subjects receiving anticoagulants. If INR measurement is obtained, the value is to be recorded on the SAE form.

12.3.3. Definition of Cardiovascular Events

**Cardiovascular Events (CV) Definition:**
Investigators will be required to fill out the specific CV event page of the CRF for the following AEs and SAEs:<br>• Myocardial infarction/unstable angina<br>• Congestive heart failure<br>• Arrhythmias<br>• Valvulopathy<br>• Pulmonary hypertension<br>• Cerebrovascular events/stroke and transient ischemic attack<br>• Peripheral arterial thromboembolism<br>• Deep venous thrombosis/pulmonary embolism<br>• Revascularization

12.3.4. Recording of AEs and SAEs

**AEs and SAE Recording:**
• When an AE/SAE occurs, it is the responsibility of the investigator (subinvestigator) to review all documentation (e.g., hospital progress notes, laboratory, and diagnostics reports) relative to the event.<br><br>• The investigator (subinvestigator) will then record all relevant information regarding an AE/SAE in the CRF.<br><br>• It is **not** acceptable for the investigator (subinvestigator) to send photocopies of the subject's medical records to GSK in lieu of completion of the GSK, AE/SAE CRF page.<br><br>• There may be instances when copies of medical records for certain cases are requested by GSK. In this instance, all subject identifiers, with the exception of the subject number, will be blinded on the copies of the medical records prior to submission of to GSK.<br><br>• The investigator (or subinvestigator) will attempt to establish a diagnosis of the event based on signs, symptoms, and/or other clinical information. In such cases, the diagnosis will be documented as the AE/SAE and not the individual signs/symptoms.

12.3.5. Evaluating AEs and SAEs

**Assessment of Intensity**
The investigator (subinvestigator) will make an assessment of intensity for each AE and SAE reported during the study and will assign it to one of the following categories:<br><br>• Mild: An event that is easily tolerated by the subject, causing minimal discomfort and not interfering with everyday activities.<br><br>• Moderate: An event that is sufficiently discomforting to interfere with normal everyday activities.<br><br>• Severe: An event that prevents normal everyday activities. - an AE that is assessed as severe will not be confused with an SAE. Severity is a category utilized for rating the intensity of an event; and both AEs and SAEs can be assessed as severe.<br><br>• An event is defined as 'serious' when it meets at least one of the pre-defined

outcomes as described in the definition of an SAE.

**Assessment of Causality**
• The investigator (subinvestigator) is obligated to assess the relationship between study treatment and the occurrence of each AE/SAE.<br>• A "reasonable possibility" is meant to convey that there are facts/evidence or arguments to suggest a causal relationship, rather than a relationship cannot be ruled out.<br>• The investigator (subinvestigator) will use clinical judgment to determine the relationship.<br>• Alternative causes, such as natural history of the underlying diseases, concomitant therapy, other risk factors, and the temporal relationship of the event to the study treatment will be considered and investigated.<br>• The investigator (subinvestigator) will also consult the Investigator Brochure (IB) and/or Product Information, for marketed products, in the determination of his/her assessment.<br>• For each AE/SAE the investigator (subinvestigator) **must** document in the medical notes that he/she has reviewed the AE/SAE and has provided an assessment of causality.<br>• There may be situations when an SAE has occurred and the investigator (subinvestigator) has minimal information to include in the initial report to GSK. However, **it is very important that the investigator (or subinvestigator) always make an assessment of causality for every event prior to the initial transmission of the SAE data to GSK.**<br>• The investigator (subinvestigator) may change his/her opinion of causality in light of follow-up information, amending the SAE data collection tool accordingly.<br>• The causality assessment is one of the criteria used when determining regulatory reporting requirements.

**Follow-up of AEs and SAEs**
• The investigator (subinvestigator) is obligated to perform or arrange for the conduct of supplemental measurements and/or evaluations as may be indicated or as requested by GSK to elucidate as fully as possible the nature and/or causality of the AE or SAE.

- The investigator (subinvestigator) is obligated to assist. This may include additional laboratory tests or investigations, histopathological examinations or consultation with other health care professionals.
- If a subject dies during participation in the study or during a recognized follow-up period, the investigator (subinvestigator) will provide GSK with a copy of any post-mortem findings, including histopathology.
- New or updated information will be recorded in the originally completed CRF.
- The investigator (subinvestigator) will submit any updated SAE data to GSK within the designated reporting time frames.

12.3.6. Reporting of SAEs to GSK

SAE reporting to GSK via electronic data collection tool

- Primary mechanism for reporting SAEs to GSK will be the electronic data collection tool.
- If the electronic system is unavailable for greater than 24 hours, the site will use the paper SAE data collection tool and fax it to the [Medical Monitor or the SAE coordinator].
- Site will enter the serious adverse event data into the electronic system as soon as it becomes available.
- After the study is completed at a given site, the electronic data collection tool (e.g., InForm system) will be taken off-line to prevent the entry of new data or changes to existing data.
- If a site receives a report of a new SAE from a study subject or receives updated data on a previously reported SAE after the electronic data collection tool has been taken off-line, the site can report this information on a paper SAE form or to the [Medical Monitor or the SAE coordinator] by telephone.
- Contacts for SAE receipt can be found at the beginning of this protocol on the Sponsor/Medical Monitor Contact Information page.

12.4. Appendix 4: Collection of Pregnancy Information

- Investigator (or subinvestigator) will collect pregnancy information on any female subject, who becomes pregnant while participating in this study.
- Information will be recorded on the appropriate form and submitted to GSK within 2 weeks of learning of a subject's pregnancy.
- Subject will be followed to determine the outcome of the pregnancy. The investigator (or subinvestigator) will collect follow up information on mother and infant, which will be forwarded to GSK. Generally, follow-up will not be required for longer than 6 to 8 weeks beyond the estimated delivery date.
- Any termination of pregnancy will be reported, regardless of fetal status (presence or absence of anomalies) or indication for procedure.
- While pregnancy itself is not considered to be an AE or SAE, any pregnancy complication or elective termination of a pregnancy will be reported as an AE or SAE.
- A spontaneous abortion is always considered to be an SAE and will be reported as such.
- Any SAE occurring as a result of a post-study pregnancy which is considered reasonably related to the study treatment by the investigator (or subinvestigator), will be reported to GSK as described in Appendix 4. While the investigator (or subinvestigator) is not obligated to actively seek this information in former study participants, he or she may learn of an SAE through spontaneous reporting.

Any female subject who becomes pregnant while participating

- will discontinue study medication or be withdrawn from the study.

For any female partner of a male study subject who becomes pregnant while participating:

- Investigator (or subinvestigator) will attempt to collect pregnancy information on any female partner of a male study subject who becomes pregnant while participating in this study. This applies only to subjects who are randomized to receive study medication.
- After obtaining the necessary signed informed consent from the female partner directly, the investigator (or subinvestigator) will record pregnancy information on the appropriate form and submit it to GSK within 2 weeks of learning of the partner’s pregnancy
- Partner will also be followed to determine the outcome of the pregnancy. Information on the status of the mother and child will be forwarded to GSK.

- Generally, follow-up will be no longer than 6 to 8 weeks following the estimated delivery date. Any termination of the pregnancy will be reported regardless of fetal status (presence or absence of anomalies) or indication for procedure.

12.5. Appendix 5 - Japan Specific Requirements

12.5.1. Regulatory and Ethical Considerations

The study will be conducted in accordance with the Ministerial Ordinance on the Standards for the Conduct of Clinical Trials of Medicinal Products (MHW Notification No.28 dated March 27, 1997) and the Pharmaceutical and Medical Device Act (PMD Act).

12.5.2. Informed Consent

Prior to participation in the study, the investigator (or subinvestigator) should fully inform the potential subject of the study including the written information. The investigator (or subinvestigator) should provide the subject and his/her proxy consenter ample time and opportunity to inquire about details of the study. The subject and/or his/her proxy consenter should sign and personally date the consent form. The subject may wish to consider the content of the written information at home. The person who conducted the informed consent discussion and the study collaborator giving supplementary explanation, where applicable, should sign and personally date the consent form. If an impartial witness is required, the witness should sign and personally date the consent form. The investigator (or subinvestigator) should retain this signed and dated form (and other written information) together with the source medical records, such as clinical charts (in accordance with the rules for records retention, if any, at each medical institution) and give a copy to the subject and/or his/her proxy consenter.

12.5.3. Study Period

April to August 2016 (planned)

12.6. Appendix 6 - List of Annexes

Annex 1: Study Administrative Structure

Annex 2: List of Medical Institutions and Investigators

Annex 3: Pregnancy Notification Form

Annex 4: Pregnancy Follow-up Form

TITLE PAGE

Division: Worldwide Development

Information Type: Protocol Amendment

**Title:** An Open Label, Single-arm Study Evaluating a New Thermostable Formulation of FLOLAN™ in Japanese Subjects with Pulmonary Arterial Hypertension (PAH)

Compound Number: AH21461

Development Phase: IV

Effective Date: 17-FEB-2016

Protocol Amendment Number: 01

Author(s): PPD

Revision Chronology

GlaxoSmithKline Document Number	Date	Version
2015N240404_00	2015-DEC-03	Original
2015N240404_01	2016-FEB-17	Amendment Number 01
The primary changes reflected in Amendment Number 01 were made to modify description of secondary endpoint (WHO Fnctional Class), to add non-serious AEs assessed as related to the investigational product to AE-related sections, and to make description adjustments.		

Copyright 2016 the GlaxoSmithKline group of companies. All rights reserved. Unauthorised copying or use of this information is prohibited.

SPONSOR SIGNATORY:

PPD

Feb 17, 2016

Hiromu Nakajima PPD
Head, Medicines Developme PPD

Date

PPD

MEDICAL MONITOR/SPONSOR INFORMATION PAGE

Medical Monitor/SAE and non-serious AE assessed as related to the investigational product Contact Information:

Role	Name	Day Time Phone Number and email address	After-hours Phone/Cell/ Pager Number	Fax Number	Site Address
Primary Medical Monitor	PPD	PPD<br>PPD	Not applicable	PPD<br>PPD	6-15, Sendagaya 4-chome, Shibuya-ku, Tokyo 151-8566
Secondary Medical Monitor	PPD	PPD<br>PPD	Not applicable	PPD<br>PPD	6-15, Sendagaya 4-chome, Shibuya-ku, Tokyo 151-8566
Contact information	Refer to Annex 1: Study Administrative Structure				

Regulatory Agency Identifying Number(s): Not applicable

INVESTIGATOR PROTOCOL AGREEMENT PAGE

For protocol number FLR201614

- I confirm agreement to conduct the study in compliance with the protocol.
- I acknowledge that I am responsible for overall study conduct. I agree to personally conduct or supervise the described study.
- I agree to ensure that all associates, colleagues and employees assisting in the conduct of the study are informed about their obligations. Mechanisms are in place to ensure that site staff receives the appropriate information throughout the study.

Investigator Name:

Study Centre Address:

Study Centre Phone Number:

Investigator Signature: Date:

TABLE OF CONTENTS

PAGE

1. PROTOCOL SYNOPSIS FOR STUDY FLR201614 ... 8

2. INTRODUCTION ... 11
 2.1. Study Rationale ... 11
 2.2. Brief Background ... 11

3. OBJECTIVES AND ENDPOINTS ... 12

4. STUDY DESIGN ... 13
 4.1. Overall Design ... 13
 4.2. Treatment Arms and Duration ... 13
 4.3. Type and Number of Subjects ... 14
 4.4. Design Justification ... 14
 4.5. Dose Justification ... 14
 4.6. Benefit:Risk Assessment ... 15
  4.6.1. Risk Assessment ... 16
  4.6.2. Benefit Assessment ... 19
  4.6.3. Overall Benefit:Risk Conclusion ... 19

5. SELECTION OF STUDY POPULATION AND WITHDRAWAL CRITERIA ... 19
 5.1. Inclusion Criteria ... 19
 5.2. Exclusion Criteria ... 21
 5.3. Screening Failures ... 22
 5.4. Withdrawal/Stopping Criteria ... 22
  5.4.1. Liver Chemistry Stopping Criteria ... 23
 5.5. Subject and Study Completion ... 23

6. STUDY TREATMENT ... 24
 6.1. Investigational Product ... 24
 6.2. Treatment Assignment ... 24
 6.3. Subject Specific Dose Adjustment Criteria ... 24
 6.4. Blinding ... 25
 6.5. Packaging and Labeling ... 25
 6.6. Preparation/Handling/Storage/Accountability ... 25
 6.7. Compliance with Study Treatment Administration ... 26
 6.8. Treatment of Study Treatment Overdose ... 26
 6.9. Treatment after the End of the Study ... 26
 6.10. Concomitant Medications and Non-Drug Therapies ... 26
  6.10.1. Permitted Medications and Non-Drug Therapies ... 27
  6.10.2. Prohibited Medications and Non-Drug Therapies ... 27

7. STUDY ASSESSMENTS AND PROCEDURES ... 27
 7.1. Time and Events Table ... 28
 7.2. Screening and Critical Baseline Assessments ... 31
 7.3. Efficacy ... 31
  7.3.1. Haemodynamics ... 31
  7.3.2. WHO Functional Class ... 32
  7.3.3. N-terminal Pro B-type Natriuretic Peptide: NT-pro BNP ... 32
 7.4. Safety ... 32

7.4.1. Adverse Events (AE) and Serious Adverse Events (SAEs) 32
7.4.1.1. Time Period and Frequency for Collecting AE and SAE Information 32
7.4.1.2. Method of Detecting AEs and SAEs 33
7.4.1.3. Follow-up of AEs and SAEs 33
7.4.1.4. Cardiovascular and Death Events 33
7.4.1.5. Regulatory Reporting Requirements for AEs and SAEs 34
7.4.2. Pregnancy 34
7.4.3. Physical Exams 34
7.4.4. Vital Signs 34
7.4.5. Oxygen Saturation 34
7.4.6. Electrocardiogram (ECG) 35
7.4.7. Clinical Safety Laboratory Assessments 35
7.5. Other Endpoints 35
7.5.1. Reasons for Dose Adjustment after Switch 35

8. DATA MANAGEMENT 35

9. STATISTICAL CONSIDERATIONS AND DATA ANALYSES 36
9.1. Hypotheses 36
9.2. Sample Size Considerations 36
9.2.1. Sample Size Assumptions 36
9.2.2. Sample Size Sensitivity 36
9.2.3. Sample Size Re-estimation or Adjustment 36
9.3. Data Analysis Considerations 36
9.3.1. Analysis Populations 36
9.3.2. Analysis Data Sets 36
9.3.3. Treatment Comparisons 36
9.3.4. Interim Analysis 37
9.4. Key Elements of Analysis Plan 37
9.4.1. Primary Analyses 37
9.4.2. Secondary Analyses 37
9.4.3. Other Analyses 37

10. STUDY GOVERNANCE CONSIDERATIONS 38
10.1. Posting of Information on Publicly Available Clinical Trial Registers 38
10.2. Regulatory and Ethical Considerations, Including the Informed Consent Process 38
10.3. Quality Control (Study Monitoring) 39
10.4. Quality Assurance 39
10.5. Study and Site Closure 40
10.6. Records Retention 40
10.7. Provision of Study Results to Investigators, Posting of Information on Publically Available Clinical Trials Registers and Publication 41

11. REFERENCES 42

12. APPENDICES 43
12.1. Appendix 1 – Abbreviations and Trademarks 43
12.2. Appendix 2: Liver Safety Required Actions and Follow up Assessments 45

12.3. Appendix 3: Definition of and Procedures for Recording, Evaluating, Follow-Up and Reporting of Adverse Events 47
12.3.1. Definition of Adverse Events 47
12.3.2. Definition of Serious Adverse Events 48
12.3.3. Definition of Cardiovascular Events 50
12.3.4. Recording of AEs and SAEs 51
12.3.5. Evaluating AEs and SAEs 51
12.3.6. Reporting of SAEs and non-serious AEs assessed as related to the investigational product (reformulated diluent for FLOLAN) to GSK 53
12.4. Appendix 4: Collection of Pregnancy Information 55
12.5. Appendix 5 - Japan Specific Requirements 56
12.5.1. Regulatory and Ethical Considerations 56
12.5.2. Informed Consent 56
12.5.3. Study Period 56
12.6. Appendix 6 - List of Annexes 57
12.7. Appendix 7 - Protocol Amendment Changes 58

1. PROTOCOL SYNOPSIS FOR STUDY FLR201614

Rationale

This study is a Phase IV, open-label, single-arm study to assess the safety and the necessity of dose adjustment after switching to FLOLAN™ prepared with the reformulated diluent in Japanese patients with pulmonary arterial hypertension (PAH) who are receiving higher doses of FLOLAN than in other countries.

Objectives/Endpoints

The objectives and endpoints of this study are as follows:

Objectives	Endpoints
**Primary**	
• To evaluate the safety and tolerability of the thermostable formulation of FLOLAN (i.e., FLOLAN prepared with the reformulated diluent) when switched from the existing FLOLAN treatment (i.e., FLOLAN prepared with the currently marketed diluent).	• Incidence and severity of Adverse Events (AEs)<br>• Laboratory parameters<br>• 12-lead ECGs<br>• Vital signs<br>• Oxygen saturation
**Secondary**	
• To evaluate the dose adjustment requirements in patients switching from the existing FLOLAN treatment to the thermostable formulation of FLOLAN.	• The number of events to adjust dose of FLOLAN based on the change from baseline to 3 hours in mean Pulmonary Artery Pressure (mPAP) in a subset of subjects*<br>• Reason for changing the dose of thermostable formulation of FLOLAN
• To evaluate the continued efficacy after switching to the thermostable formulation of FLOLAN.	• Change from baseline to Week4 in NT pro BNP<br>• World Health Organization (WHO) Functional Class frequency by visits and change from previous visit<br>• Changes from baseline up to 24 hours and at Week 4 in Haemodynamic parameters: mean Pulmonary Artery

Objectives	Endpoints
	Pressure (mPAP), Pulmonary Vascular resistance (PVR), Cardiac Output (CO), Right Atrial Pressure (RAP) in a subset of subjects*

* Subjects who consent to undergo right heart catheterisation (RHC) over 24-hour and at week 4.

Overall Design

- Phase IV, open-label, single-arm study
- Study Population: Patients who are receiving FLOLAN for the treatment of PAH

Figure 1 Study Schematic

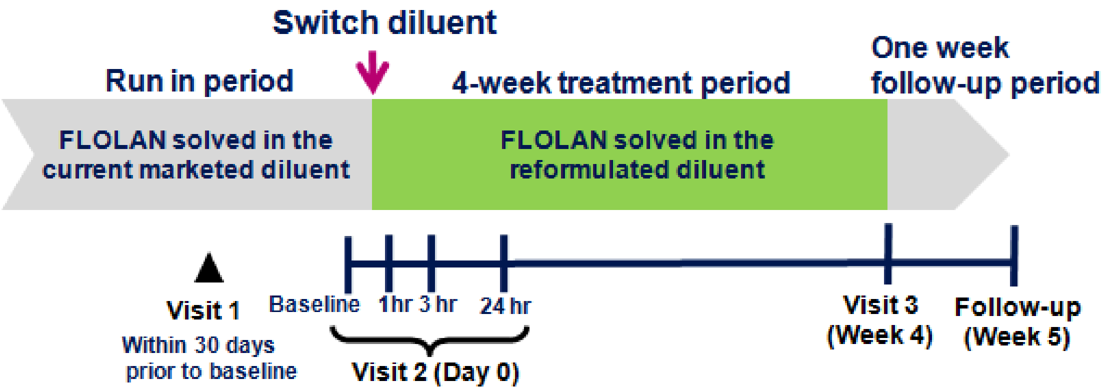

Treatment Arms and Duration

The study will include a screening visit, a run-in period of a maximum of 4 weeks with the existing FLOLAN treatment (i.e., FLOLAN prepared with the currently marketed diluent), a 4-week treatment period with the thermostable formulation of FLOLAN (i.e., FLOLAN prepared with the reformulated diluent) and a one-week follow-up visit.

After the run-in period, subjects undergo baseline assessments at the study centre and switch the study treatment to the thermostable formulation of FLOLAN. Subjects stay overnight in the centre for checking clinical symptoms and haemodynamic stability up to 24 hours after switching the treatment. Subject completion is defined as completion of Week 4.

Period	Description
Run-in	FLOLAN prepared with the currently marketed diluent (epoprostenol sodium+ pH 10.2 – 10.8 diluent) for Injection
Treatment	FLOLAN prepared with the reformulated diluent (epoprostenol sodium + pH 11.7 – 12.3 diluent) for injection

GSK is not providing specific post-study treatment. The post-study care will be decided at the discretion of the investigator (or subinvestigator) based on the patient's condition.

Type and Number of Subjects

Subjects with PAH who have been receiving FLOLAN at 45 ng/kg/min or higher doses will be eligible.

Adequate number of subjects will be enrolled in the study in order to have 10 subjects to complete assessments at 4 weeks, including at least 5 subjects as a subset of subjects who consent to undergo right heart catheterisation (RHC) over 24-hour and at Week 4. Given the nature of the disease the sample size for this study was chosen based on feasibility and no specific hypothesis testing is planned.

Analysis

Data will be summarized using descriptive statistics.

The analysis population will be the Intention-to-Treat (ITT) population consisting of all subjects who have received at least one dose of the thermostable formulation of FLOLAN. The ITT population will be used for all efficacy and safety summaries.

Primary analyses will be frequency tabulation or summary statistics calculation for safety (AEs, clinical laboratory tests [haematology, clinical chemistry and urinalysis], vital signs [systolic and diastolic blood pressure, heart rate], 12-lead ECG, oxygen saturation) and tolerability on the ITT population.

Secondary analyses will be tabulation of the number of FLOLAN dose adjustment events and WHO Functional Class frequency by visits and change from previous visit, as well as calculation of summary statistics for the change from baseline for other secondary endpoints.

2. INTRODUCTION

2.1. Study Rationale

This study is a Phase IV, open-label, single-arm study to assess the safety and the necessity of dose adjustment after switching to FLOLAN prepared with the reformulated diluent in Japanese patients with pulmonary arterial hypertension (PAH) who are receiving higher doses of FLOLAN than in other countries.

2.2. Brief Background

FLOLAN is an effective treatment for PAH and reduces mortality; however, safe and effective administration is complex and requires a considerable level of commitment from patients. The currently marketed formulation of FLOLAN requires that the reconstituted drug with diluent specific to FLOLAN should be administered within a 24 hour period and maintained between a temperature of 2 and 8 °C during infusion, necessitating the use of a cold pack. In addition, the cold pack used to maintain the temperature of the reconstituted drug requires frequent changes.

A new formulation of FLOLAN diluent has been developed, which increases the stability of FLOLAN infusion solution at ambient temperatures as the pH of the diluent has been increased from 10.5 (currently marketed diluent, pH 10.2 – 10.8) to 12 (reformulated diluent, pH11.7 – 12.3). Freshly prepared solutions reconstituted and diluted with the reformulated diluent, or solutions that have been stored at 2°C to 8°C (for no longer than 8 days) can be administered up to 24 hours at up to 35°C or up to 72 hours at up to 25°C; it does not, therefore, require the use of a cold pack or frequent changes of the cassette. In this way, it is anticipated that the new formulation will provide an added level of convenience to patients through reduction in the frequency of reconstitution, and elimination of the need for a cold pack, even in countries with relatively high ambient temperatures. The change is limited to the diluent and the active ingredient remains the same as in the current formulation, therefore the new thermostable formulation is not expected to have any impact on the pharmacodynamic actions of FLOLAN and the clinical profile is expected to be the same as that of the current formulation. Actually, the previous overseas study of FLR115322 demonstrated that no dose adjustment was required for switching from the current diluent to the reformulated diluent and similar safety and tolerability of the reformulated diluent in Caucasian PAH patients [Provencher, 2015].

However, there is no experience in Japanese PAH patients for switching to the diluent to be used in this current study. Additionally, relatively higher dosage of FLOLAN is used for Japanese adult patients to manage and inhibit the progression of PAH as compared with those of overseas of 20 – 40 ng/kg/min [Ito, 2012; Galiè, 2009; McLaughlin, 2009]. The post-marketing surveillance in 1999 to 2009 in Japan shows that the longer time the patient has been treated with FLOLAN, the higher doses the patient has been administered in management of PAH. The evidence of the benefit for PAH patients with higher doses of FLO LAN have been reported. Ogawa et al reported that high dose FLOLAN of mean 79.6 ng/kg/min showed significant long-term survival when compared

with dose of mean 54.0 ng/kg/min [Ogawa, 2014]. Also, Akagi et al demonstrated that high dose FLOLAN therapy improved the level of haemodynamic parameters [Akagi, 2010].

This current study will evaluate the safety and tolerability after switching from the current diluent to the reformulated diluent in Japanese PAH patients who have been receiving higher doses of FLOLAN than in other countries.

3. OBJECTIVES AND ENDPOINTS

The objectives and endpoints of this study are as follows:

Objectives	Endpoints
**Primary**	
• To evaluate the safety and tolerability of the new thermostable formulation of FLOLAN (i.e., FLOLAN prepared with the reformulated diluent) when switched from the existing FLOLAN treatment (i.e., FLOLAN prepared with the currently marketed diluent).	• Incidence and severity of Adverse Events (AEs)<br>• Laboratory parameters<br>• 12-lead ECGs<br>• Vital signs<br>• Oxygen saturation
**Secondary**	
• To evaluate the dose adjustment requirements in patients switching from the existing FLOLAN treatment to the thermostable formulation of FLOLAN.	• The number of events to adjust dose of FLOLAN based on the change from baseline to 3 hours in mean Pulmonary Artery Pressure (mPAP) in a subset of subjects<br>• Reason for the change dose of the new thermostable formulation of FLOLAN
• To evaluate the continued efficacy after switching to the thermostable formulation of FLOLAN.	• Change from baseline to Week4 in NT pro BNP<br>• WHO Functional Class distribution by visits and change from previous visit<br>• Changes from baseline up to 24 hours and at Week 4 in Haemodynamic parameters: mean Pulmonary Artery Pressure (mPAP), Pulmonary Vascular resistance (PVR),

Objectives	Endpoints
	Cardiac Output (CO), Right Atrial Pressure (RAP) in a subset of subjects*

* Subjects who consent to undergo right heart catheterisation (RHC) over 24-hour and at week 4.

4. STUDY DESIGN

4.1. Overall Design

- Phase IV, open-label, single-arm study
- Study Population: Patients who are receiving FLOLAN for the treatment of PAH

Figure 2 Study Schematic

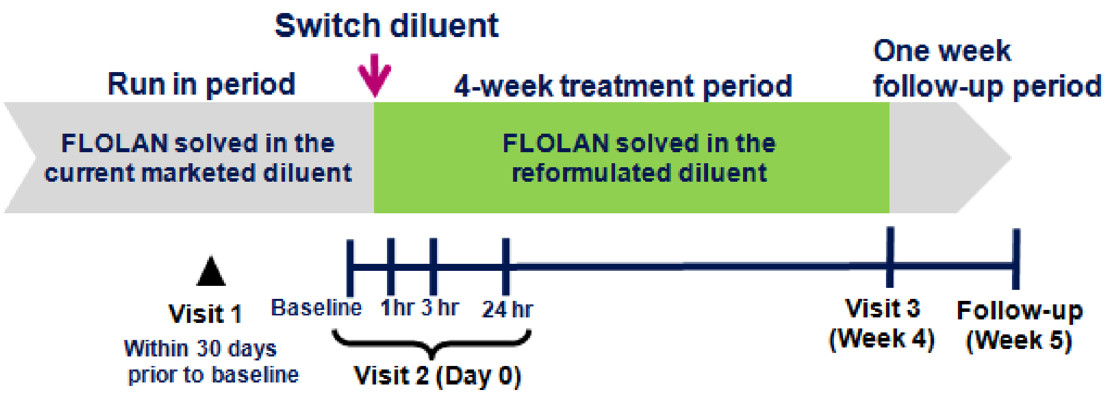

4.2. Treatment Arms and Duration

The study will include a screening visit, a run-in period of a maximum of 4 weeks with the existing FLOLAN treatment (i.e., FLOLAN prepared with the currently marketed diluent), a 4-week treatment period with the thermostable formulation of FLOLAN (i.e., FLOLAN prepared with the reformulated diluent) and a one-week follow-up visit.

After the run-in period, subjects undergo baseline assessments at the study centre and switch the study treatment to the thermostable formulation of FLOLAN. Subjects stay overnight in the centre for checking clinical symptoms and haemodynamic stability up to 24 hours after switching the treatment. Subject completion is defined as completion of Week 4.

Period	Description
Run-in	FLOLAN prepared with the currently marketed diluent (epoprostenol sodium+ pH 10.2 – 10.8 diluent) for Injection
Treatment	FLOLAN prepared with the reformulated diluent (epoprostenol sodium + pH 11.7 – 12.3 diluent) for Injection

GSK is not providing specific post-study treatment. The post-study care will be decided at the discretion of the investigator (or subinvestigator) based on the patient's condition.

4.3. Type and Number of Subjects

Subjects with PAH who are receiving FLOLAN at 45 ng/kg/min or higher doses will be eligible.

Adequate number of subjects will be enrolled in the study in order to have 10 subjects to complete assessments at 4 weeks, including at least 5 subjects as a subset of subjects who consent to undergo right heart catheterisation (RHC) over 24-hour and at Week 4. Given the nature of the disease the sample size for this study was chosen based on feasibility and no specific hypothesis testing is planned.

4.4. Design Justification

PAH is a life threatening disease and FLOLAN is an important treatment for many patients with PAH. The purpose of this study is to assess the safety and the necessity of dose adjustment after switching to FLOLAN prepared with the reformulated diluent in Japanese PAH patients who are receiving higher doses of FLOLAN than in other countries. The primary endpoints are the incidence and severity of AEs, 12-lead ECG, vital signs, and oxygen saturation. The secondary endpoints are NT-proBNP, WHO Functional Class, and haemodynamic parameters to monitor PAH control expected with FLOLAN, a standard PAH treatment in Japan.

As the study also seeks to support that the new formulation of FLOLAN diluent is functionally comparable to the existing formulation, it is important that subjects enrolled are sufficiently stable to allow meaningful comparison between baseline and after treatment. Based on feasibility, an open-label single-arm study has been selected.

The 4-week treatment period was chosen to allow patients sufficient length of time to learn to set up and administer the new thermostable formulation of FLOLAN and to appreciate any meaningful differences the new formulation may bring to their activities of daily living.

4.5. Dose Justification

This section is not applicable as the investigational product (IP) to be assessed by this study is the reformulated diluent for FLOLAN.

4.6. Benefit:Risk Assessment

The pivotal clinical trials evaluating the approved indications and populations for FLOLAN were carried out largely in the 1980’s and 1990’s. Summaries of findings from both clinical and non-clinical studies conducted with FLOLAN can be found in the Package Insert.

The safety profile of FLOLAN is supported by more than 30 years of post-marketing experience (international birthdate 18 March 1981). Post-marketing safety information is described within the current Package Insert. It is important to note that only the diluent for FLOLAN will change in this study, the vials containing the FLOLAN will not change either during this study or following approval of the reformulated diluent. FLOLAN has a number of risks associated with both the pharmacologic activity of FLOLAN, as well as the administration system. The safety issues described are known risks that are not likely to adversely affect the overall benefit:risk assessment for the use of FLOLAN in PAH (WHO Group 1). The following section outlines the risk assessment and mitigation strategy for this protocol:

4.6.1. Risk Assessment

Potential Risk of Clinical Significance	Summary of Data/Rationale for Risk	Mitigation Strategy
**FLOLAN**		
Medication errors*	FLOLAN preparation and administration is complex, highly individualized, and continuous, all of which increase the risk of medication errors. Reconstitution of FLOLAN with the current sterile diluent for FLOLAN followed by subsequent prolonged refrigeration or infusion over 24 hours without use of a cold pouch could result in reduced efficacy including possible rebound of PAH symptoms due to decreased room temperature stability of FLOLAN when mixed with the pH 10.5 diluent.	GSK will provide the pH 12 diluent. Patients recruited to this study must be on stable and relatively high doses of FLOLAN. Consequently, patients should have a central line in place, be aware of the routine care required for a central line and aware of the risks of FLOLAN therapy.
Local infusion site reactions during long-term infusion**	Because of the high pH of the final FLOLAN infusion solutions, care should be taken to avoid extravasation during administration and consequent risk of tissue damage. The infusion rate of FLOLAN is extremely low when compared with blood flow and the buffer capacity of the FLOLAN solution for infusion is not changed when reconstituted with the reformulated pH 12 diluent for FLOLAN, therefore it is anticipated that the solution will drop to physiologic pH upon mixing with the blood. In addition, neither the in vitro haemolysis study, the in vivo dog study or the small clinical trial in patients who received	Injection sites will be checked at each clinic visit for any reactions.

Potential Risk of Clinical Significance	Summary of Data/Rationale for Risk	Mitigation Strategy
	FLOLAN prepared with pH 12 diluent identified concerns with regards to local tolerance.	
Sepsis, septicaemia	Patients should aware that FLOLAN therapy may require prolonged periods and is complex (e.g., sterile drug reconstitution and permanent indwelling of a central venous catheter).	The inclusion criteria specify that only patients who have been on FLOLAN therapy for PAH are eligible for this study.
Pulmonary oedema	Some patients with PAH have developed pulmonary oedema during dose-ranging (dose initiation), which may be associated with pulmonary veno-occlusive disease. If pulmonary oedema occurs, the infusion of FLOLAN should be discontinued.	The inclusion criteria specify that only patients who have been on FLOLAN therapy for PAH are eligible for this study.<br>The withdrawal criteria specify that treatment should be discontinued if it is not in the best interest of the patient to participate in this study due to the onset of an AE.<br>Vital signs will be monitored continuously by measurement at each clinic visit.
Bleeding events at various sites	FLOLAN is a potent inhibitor of platelet aggregation, therefore, an increased risk for haemorrhagic complications should be considered, particularly for patients with other risk factors for bleeding.	The inclusion criteria specify that only patients who have been on FLOLAN therapy for PAH are eligible for this study.<br>Vital signs will be monitored continuously by measurement at each clinic visit.
Tachycardia<br>Bradycardia	FLOLAN is a potent pulmonary and systemic vasodilator. The cardiovascular effects during infusion disappear within 30 min of the end of administration. FLOLAN may either decrease or	Vital signs will be monitored continuously by measurement at each clinic visit.

Potential Risk of Clinical Significance	Summary of Data/Rationale for Risk	Mitigation Strategy
	increase heart rate. Blood pressure and heart rate should be monitored during administration of FLOLAN.	

*This safety concern may be more likely to occur during the transition from pH 10.5 diluent to pH 12 diluent.
**This safety concern may be more likely to occur with use of FLOLAN solution prepared with pH12 diluent.

4.6.2. Benefit Assessment

Chronic continuous FLOLAN infusion is an effective treatment for PAH (group 1) patients in NYHA Functional Classes III and IV.

This effectiveness has been demonstrated by significant increases in exercise capacity (idiopathic, heritable, and PAH associated with connective tissue diseases) and in survival (idiopathic and heritable PAH) in patients treated with FLOLAN chronically.

Overall, the complexities involved with maintaining the infusion pump and catheter, and reconstituting FLOLAN do not negate the symptomatic benefits of chronic FLOLAN treatment. Use of the reformulated diluent will eliminate the requirement for use of a cold pack, and potentially decrease both the frequency of reconstitution and medication cassette exchanges, thereby potentially slightly simplifying FLOLAN administration.

4.6.3. Overall Benefit:Risk Conclusion

Subjects recruited to this trial are patients with PAH who are on stable and relatively high doses of FLOLAN, and therefore are those patients for whom FLOLAN is effective. In view of the patient population identified for enrollment, GSK assesses that the benefits and risks balance of FLOLAN therapy, including in the context of this study, for the treatment of PAH (Group 1) is favourable.

5. SELECTION OF STUDY POPULATION AND WITHDRAWAL CRITERIA

Specific information regarding warnings, precautions, contraindications, AEs, and other pertinent information on the GSK investigational product or other study treatment that may impact subject eligibility is provided in the product label.

Deviations from inclusion and exclusion criteria are not allowed because they can potentially jeopardize the scientific integrity of the study, regulatory acceptability or subject safety. Therefore, adherence to the criteria as specified in the protocol is essential.

5.1. Inclusion Criteria

A subject will be eligible for inclusion in this study only if all of the following criteria apply:

1. Male or female subjects at least 18 to 75 years at the time of screening;
2. Subjects must be on FLOLAN therapy for pulmonary arterial hypertension (PAH) as approved in the product label;
3. Subjects must receive FLOLAN at 45 ng/kg/min or higher;
4. Subjects must be on stable doses of their existing FLOLAN treatment for a minimum of one month prior to screening; it is acceptable to adjust within 10% of dose during the last one month period;

5. Subjects must be on stable doses of any current PAH treatments other than FLOLAN in the last 30 days prior to screening;

6. Subjects who meet any of the following:

   1) A female subject is eligible to participate if she is not pregnant (as confirmed by a negative [serum or urine] human chorionic gonadotrophin (hCG) test), not lactating, and at least one of the following conditions applies:

   a) Non-reproductive potential defined as:

      - Pre-menopausal females with any of the following: documented tubal ligation, documented hysteroscopic tubal occlusion procedure with follow-up confirmation of bilateral tubal occlusion, hysterectomy, or documented bilateral oophorectomy.

      - Postmenopausal defined as 12 months of spontaneous amenorrhea [in questionable cases a blood sample with simultaneous follicle stimulating hormone (FSH) and estradiol levels consistent with menopause (refer to laboratory reference ranges for confirmatory levels)]. Females on hormone replacement therapy (HRT) and whose menopausal status is in doubt will be required to use one of the highly effective contraception methods if they wish to continue their HRT during the study. Otherwise, they must discontinue HRT to allow confirmation of post-menopausal status prior to study enrolment.

   b) Reproductive potential and agrees to follow one of the options listed below in the GSK Modified List of Highly Effective Methods for Avoiding Pregnancy in Females of Reproductive Potential (FRP) from 30 days prior to the first dose of thermostable formulation of FLOLAN until completion of the follow-up visit.

GSK Modified List of Highly Effective Methods for Avoiding Pregnancy in Females of Reproductive Potential (FRP)

This list does not apply to FRP with same sex partners, when this is their preferred and usual lifestyle or for subjects who are and will continue to be abstinent from penile-vaginal intercourse on a long term and persistent basis.

- Contraceptive subdermal implant that meets the GSK effectiveness criteria including a <1% rate of failure per year, as stated in the product label

- Intrauterine device or intrauterine system that meets the GSK effectiveness criteria including a <1% rate of failure per year, as stated in the product label [Hatcher, 2007a]

- Oral contraceptive, either combined or progestogen alone [Hatcher, 2007a]

- Injectable progestogen [Hatcher, 2007a]

- Contraceptive vaginal ring [Hatcher, 2007a]
- Percutaneous contraceptive patches [Hatcher, 2007a]
- Male partner sterilization with documentation of azoospermia prior to the female subject's entry into the study, and this male is the sole partner for that subject [Hatcher, 2007a]. [Male infertility information will be obtained from a review of the subject's medical records by site staff, subject's medical examinations and/or semen analysis, or an interview with the subject about medical history.]
- Male condom combined with a vaginal spermicide (foam, gel, film, cream, or suppository) [Hatcher, 2007b]

These allowed methods of contraception are only effective when used consistently, correctly and in accordance with the product label. The investigator (or subinvestigator) is responsible for ensuring that subjects understand how to properly use these methods of contraception.

7. Subject must agree not to participate in a clinical study involving another investigational drug or device throughout this study;
8. Subjects must be competent to understand the information given in the Institutional Review Board (IRB) or Independent Ethics Committee (IEC) approved informed consent form and must sign the form prior to the initiation of any study procedures.

5.2. Exclusion Criteria

A subject will not be eligible for inclusion in this study if any of the following criteria apply:

1. Subjects who are given FLOLAN for a condition or in a manner that is outside the approved indication.
2. Subjects with congestive heart failure arising from severe left ventricular dysfunction.
3. Subjects, with or without supplemental oxygen, who have a resting arterial oxygen saturation (SaO2) <90% as measured by pulse oximetry at screening.
4. Subjects have been hospitalised as an emergency or visited the emergency room for a condition related to PAH or treatment for PAH in the last 3 months.
5. The subject’s clinical condition is such that they are not expected to remain clinically stable for the duration of the study.
6. Female subjects who are pregnant or breastfeeding.
7. Subjects who have demonstrated noncompliance with previous medical regimens.
8. Subjects who have a history of abusing alcohol or illicit drugs within 1 year.

9. Subjects who have participated in a clinical study involving another investigational drug or device within four weeks before screening.

10. Any concurrent condition that would affect the safety of the subject or in the opinion of the investigator (or subinvestigator) it is not in the best interest of the patient to participate in the study.

5.3. Screening Failures

Screen failures are defined as subjects who consent to participate in the clinical trial but never received treatment with the thermostable formulation of FLOLAN.

In order to ensure transparent reporting of screen failure subjects, meet the Consolidated Standards of Reporting Trials (CONSORT) publishing requirements, and respond to queries from Regulatory authorities, a minimal set of screen failure information is required including Demography, Screen Failure details, Eligibility Criteria, and Serious Adverse Events.

5.4. Withdrawal/Stopping Criteria

Subjects who do not tolerate treatment will be withdrawn from the study. Treatment for subjects withdrawn from the study will be implemented at the investigator's (or subinvestigator’s) discretion.

A subject may also be discontinued prior to completion of the study for the following reasons, but not limited to:

- Adverse event (AE) or serious adverse event (SAE) which in the opinion of the investigator (or subinvestigator) requires withdrawal
- Termination of study by Sponsor
- Lost to follow-up
- Investigator (or subinvestigator)’s discretion
- Consent withdrawn
- Completion of study at study centre

The investigator (or subinvestigator) must take the following actions in relation to a subject who fails to attend the clinic for a required study visit:

- The investigator (or subinvestigator) must attempt to contact the subject and re-schedule the missed visit as soon as possible.
- The investigator (or subinvestigator) must counsel the subject on the importance of maintaining the assigned visit schedule and ascertain whether or not the subject wishes to and/or should continue in the study.

- In cases where the subject is deemed 'lost to follow up', the investigator (or subinvestigator) or designee must make every effort to regain contact with the subject (where possible, 3 telephone calls and if necessary a certified letter to the subject's last known mailing address or local equivalent methods). These contact attempts should be documented in the subject's medical record.
- Should the subject continue to be unreachable, only then will he/she be considered to have withdrawn from the study with a primary reason of "Lost to Follow-up".

A subject may withdraw from study treatment at any time at his/her own request, or may be withdrawn at any time at the discretion of the investigator (or subinvestigator) for safety, behavioural or administrative reasons. If a subject withdraws from the study, he/she may request destruction of any samples taken, and the investigator (or subinvestigator) must document this in the site study records.

5.4.1. Liver Chemistry Stopping Criteria

Liver chemistry stopping criteria are not established to prevent aggravation of PAH due to the discontinuation of FLOLAN and to ensure the safety of subjects of this study.

Phase III-IV liver chemistry increased monitoring criteria have been designed to assure subject safety and evaluate liver event etiology (in alignment with the FDA premarketing clinical liver safety guidance).

Required actions and follow-up assessments after liver stopping or monitoring event are given in Appendix 2.

5.5. Subject and Study Completion

A completed subject is one who has completed all phases of the study including the follow-up visit. The end of the study is defined as the last subject's last visit.

6. STUDY TREATMENT

6.1. Investigational Product

Product name:	FLOLAN for injection 0.5 mg	FLOLAN for injection 1.5 mg	Reformulated diluent for FLOLAN
Ingredient*/content (as epoprostenol)	0.531mg (0.5 mg)	1.593 mg (1.5 mg)	One vial contains glycine (94 mg), sodium chloride, and sodium hydroxide (pH adjusters).
Excipients (quantity per vial)	D-mannitol (50 mg), glycine (3.76 g), sodium chloride, and sodium hydroxide (pH adjusters)		Glycine (94 mg), sodium chloride, and sodium hydroxide (pH adjusters)
Physical description:	White lyophylised powder or lump powder for injection		
	The pH, osmotic and pressure ratio of the resultant solution when one vial of FLOLAN (0.5 mg, 1.5 mg) is dissolved in 50 mL of the diluent for FLOLAN and the description of the resultant solution when one vial of FLOLAN (0.5 mg, 1.5 mg) is dissolved in 10 mL of the diluent for FLOLAN are as follows:<br><br>pH, 11.7 to 12.3; osmotic pressure ratio, 0.3 to 0.5; description, colorless and clear		

* Epoprostenol sodium

GSK will provide the reformulated diluent for FLOLAN during the study. GSK will not provide FLOLAN, i.e. epoprostenol 0.5 and 1.5 mg lyophile.

6.2. Treatment Assignment

This will be a single-arm open-label study and there will be no treatment assignment.

6.3. Subject Specific Dose Adjustment Criteria

Subjects are monitored regularly to adjust the infusion rate according to symptoms. If required, the dose should be increased or decreased by 1 to 2 ng/kg/min at intervals of at least 15 minutes while monitoring the patient's condition (e.g., symptoms, blood pressure, heart rate, and haemodynamics).

Adverse drug reactions (ADRs) noticed during the administration of FLOLAN (see the product label) are important parameters to decide the optimal rate of infusion. The optimal rate of infusion should be decided through adequate monitoring for ADRs.

6.4. Blinding

This will be a single-arm open-label study and there will be no blinding.

6.5. Packaging and Labeling

A description of packaging and labeling of the investigational product is detailed in a document describing the handling and management of investigational product provided in the Study Reference Manual (SRM).

The contents of the label will be in accordance with all applicable regulatory requirements.

6.6. Preparation/Handling/Storage/Accountability

To prepare the new thermostable product, patients will reconstitute and dilute one or more vials of FLOLAN lyophile (according to therapeutic need) with two vials of the new sterile glycine diluent, giving 100mL of medication for each day of treatment. This reconstituted infusion is filled into medication cassettes prior to either refrigerated storage or immediate continuous intravenous infusion via a central venous catheter using an ambulatory infusion pump.

- Only subjects enrolled in the study may receive the investigational product (reformulated diluent for FLOLAN) or undergo treatment with FLOLAN prepared with the investigational product (study treatment) and only authorised site staff may supply the investigational product or administer the study treatment. All investigational product supplies must be stored in a secure environmentally controlled and monitored (manual or automated) area in accordance with the labelled storage conditions with access limited to the investigator (or subinvestigator) and authorised site staff.

- The investigator (or subinvestigator), institution, or the head of the medical institution (where applicable) is responsible for investigational product accountability, reconciliation, and record maintenance (i.e. receipt, reconciliation and final disposition records).

- Further guidance and information for final disposition of unused investigational product supplies are provided in the SRM.

- Under normal conditions of handling and administration, the investigational product is not expected to pose significant safety risks to site staff.

- A Material Safety Data Sheet (MSDS)/equivalent document describing occupational hazards and recommended handling precautions either will be provided to the investigator (or subinvestigator), where this is required by local laws, or is available upon request from the institution.

- Precaution will be taken to avoid direct contact with the investigational product. A Material Safety Data Sheet (MSDS)/equivalent document describing occupational

hazards and recommended handling precautions will be provided to the investigator (or subinvestigator).

6.7. Compliance with Study Treatment Administration

The first dose of thermostable formulation of FLOLAN will be administered to subjects at the site. Administration will be documented in the source documents and reported in the CRF.

When subjects self-administer the thermostable formulation of FLOLAN at home, compliance with the study treatment will be assessed through querying the subject during the site visits and documented in the source documents and CRF. A record of the numbers of lyophile FLOLAN vials (0.5 mg or 1.5 mg) and new FLOLAN diluents dispensed to and used by each subject must be maintained and reconciled with study treatment and compliance records.

6.8. Treatment of Study Treatment Overdose

FLOLAN injection therapy should be maintained at an optimal rate of infusion while monitoring patients at regular intervals to adjust the infusion rate according to symptoms. In this study, therefore, the definition of overdose is not established.

The product label of FLOLAN for injection 0.5 mg and 1.5 mg describes signs/symptoms of overdosage and corrective treatments as follows:

Signs and Symptoms of overdosage: In general, events seen after overdose of FLOLAN represent exaggerated pharmacological effects of the drug (e.g. hypotension and loss of consciousness).

Corrective treatments: In addition to dose reduction or discontinuation, fluid therapy, administration of catecholamine or atropine sulfate hydrate, or other necessary corrective therapies should be provided. When FLOLAN is discontinued, the infusion rate should be decreased as gradually as possible to avoid PAH symptom aggravation or recurrence.

6.9. Treatment after the End of the Study

Subjects will not receive any additional treatment from GSK after completion of the study because the study is a post-marketing clinical study.

The investigator (or subinvestigator) is responsible for ensuring that consideration has been given to the post-study care of the subject’s medical condition, whether or not GSK is providing specific post-study treatment.

6.10. Concomitant Medications and Non-Drug Therapies

All concomitant medications and non-drug therapies used by the subject are recorded in the CRF.

6.10.1. Permitted Medications and Non-Drug Therapies

Subjects taking concomitant PAH specific medications must have stable PAH therapy for at least 1 month prior to screening and the therapy will need to be maintained throughout the duration of treatment period. No changes to concomitant PAH-specific medication are allowed during the treatment period.

Examples of permitted PAH-specific medications, but not limited to:

- Vasodilators such as angiotensin-converting enzymes (ACE) inhibitors and calcium channel blockers
- Cyclic guanosine monophosphate (cGMP)-specific phosphodiesterase type 5 (PDE-5) inhibitors such as sildenafil and tadalafil
- Endothelin receptor antagonist (ERA) such as ambrisentan and bosentan

6.10.2. Prohibited Medications and Non-Drug Therapies

During the study, no other prostanoid therapy or its reformulation will be permitted.

7. STUDY ASSESSMENTS AND PROCEDURES

Protocol waivers or exemptions are not allowed with the exception of immediate safety concerns. Therefore, adherence to the study design requirements, including those specified in the Time and Events Table, are essential and required for study conduct.

This section lists the procedures and parameters of each planned study assessment. The exact timing of each assessment is listed in the Time and Events Table (Section 7.1)

7.1. Time and Events Table

	Run-in Period	Treatment Period								Follow-up visit [1]
	Visit 1	Visit 2				Phone call	Unscheduled visit	Early withdrawal	Visit 3	
Procedure	Screening (up to 30 days prior to Baseline)	Baseline (prior to RHC)	1 (+ 0.5hr)	3 (± 0.5hr)	24 (± 0.5hr)	Day 3, 7 & Week 2 (±3 days)	To conduct assessments if necessary		Week 4 (± 3 days)	(one weeks + 3 days after Week 4)
Informed consent	X									
Inclusion and exclusion criteria	X									
Demography [2]	X									
Medical history/therapeutic history/family history	X									
Disease history	X									
Physical examination (height and weight)		X						X[3]	X[3]	
Concomitant medication review	X	X				X	X	X	X	X

	Run-in Period	Treatment Period								Follow-up visit [1]
	Visit 1	Visit 2				Phone call	Unscheduled visit	Early withdrawal	Visit 3	
Procedure	Screening (up to 30 days prior to Baseline)	Baseline (prior to RHC)	1 (+ 0.5hr)	3 (± 0.5hr)	24 (± 0.5hr)	Day 3, 7 & Week 2 (±3 days)	To conduct assessments if necessary		Week 4 (± 3 days)	(one weeks + 3 days after Week 4)
Haemodynamics (RHC) [4]		X [5]	X	X	X			X[6]	X	
WHO Functional Class	X	X						X	X	
NT-pro BNP		X			X			X	X	
AE review	X	X	X	X	X	X	X	X	X	X
SAE review	X[7]	X	X	X	X	X	X	X	X	X
Injection site reactions	X	X			X			X	X	
12-lead ECG	X	X			X			X	X	
Oxygen saturation	X	X	X	X	X			X	X	
Vital signs	X	X	X	X	X			X	X	
Pregnancy test [8]	X	X						X	X	

	Run-in Period	Treatment Period								Follow-up visit [1]
	Visit 1	Visit 2				Phone call	Unscheduled visit	Early withdrawal	Visit 3	
Procedure	Screening (up to 30 days prior to Baseline)	Baseline (prior to RHC)	1 (+ 0.5hr)	3 (± 0.5hr)	24 (± 0.5hr)	Day 3, 7 & Week 2 (±3 days)	To conduct assessments if necessary		Week 4 (± 3 days)	(one weeks + 3 days after Week 4)
Laboratory assessments	X	X						X	X	
Reasons for new FLOLAN diluent dose adjustment			X	X	X		X	X	X	
Study Treatment (Assess compliance)						X	X	X	X	

RHC: right heart catheterization

1. Allow phone contact.
2. Birth year, gender, race, ethnicity
3. Weight only
4. Only subjects who have given consent to undergo RHC over 24-hour and at Week4
5. For Baseline, within 0.5 hr prior to switching to the new Flolan diluents
6. Undergo if possible
7. Any SAEs assessed as related to study participation or related to a GSK product will be collected from the time a subject consents to participate in the study.
8. Urinary or serum test for women of childbearing potential

7.2. Screening and Critical Baseline Assessments

Screening Visit (Visit 1)

Subjects on a stable dose of the existing FLOLAN therapy will be screened to determine if they meet the eligibility criteria. Subjects who are eligible for the trial will sign an informed consent prior to initiation of any study related procedures.

At the Screening Visit, cardiovascular history/risk factors (to be detailed in the CRF) will be assessed.

Information will be collected on the following demographic characteristics: birth year, gender, race and ethnicity.

Medical, disease, therapeutic and family history will be assessed against the inclusion/exclusion criteria listed in Section 5.

Subjects will enter a run-in period of a maximum of 4 weeks after the Screening visit. Subjects will come back to the hospital for the Baseline Visit within 4 weeks post Screening Visit (Visit 1).

Baseline Visit (Visit 2)

At Baseline Visit (Visit2), subjects will be switched from the existing FLOLAN therapy and will receive FLOLAN prepared with the reformulated diluent for a period of 4 weeks. All baseline procedures will be performed before the switch to the thermostable formulation of FLOLAN. Subjects will remain in the hospital for a minimum of 24 hours of treatment.

All subjects will undergo the following procedure: The switch to the thermostable formulation of FLOLAN will start with an equivalent dose of their existing FLOLAN therapy. If necessary, the investigator (or subinvestigator) will adjust the dose as appropriate for the subject until they are stable on the thermostable formulation of FLOLAN. After switching to the thermostable formulation of FLOLAN, subjects will be kept in the hospital for an observation period of at least 24 hours. During the observation period, subjects will be monitored closely for safety and haemodynamic stability up to 24 hours after the switching. If a subject cannot be stabilized, the dose of FLOLAN for a subject will be adjusted and the subject will stay at the hospital for an adequate period per the discretion of the investigator (or subinvestigator). After that treatment, the subject will be discharged and will stay in the trial. These subjects will follow the telephone call procedures as outlined below and in the Time and Events Table (Section 7.1).

7.3. Efficacy

7.3.1. Haemodynamics

Haemodynamic parameters, i.e., mean pulmonary artery blood pressure [mPAP], pulmonary vascular resistance [PVR], cardiac output [CO], and right atrial pressure

[RAP] will be assessed at baseline and at 1, 3 and 24 hours after the first dose of thermostable formulation of FLOLAN (or at early withdrawal visit if possible).

7.3.2. WHO Functional Class

WHO Functional Class will be assessed at the screening visit, baseline and Week 4 (or at early withdrawal visit). The investigator (or subinvestigator) conducting the assessment at baseline should also be responsible for the assessment at Week 4 wherever possible.

7.3.3. N-terminal Pro B-type Natriuretic Peptide: NT-pro BNP

For measurement of plasma NT-proBNP concentrations, a blood sample will be taken at baseline, 24 hours after the first dose of thermostable formulation of FLOLAN, and Week 4 (or at early withdrawal visit).

7.4. Safety

Planned time points for all safety assessments are listed in the Time and Events Table (Section 7.1). Additional time points for safety tests (such as vital signs, physical exams and laboratory safety tests) may be added during the course of the study based on newly available data to ensure appropriate safety monitoring.

7.4.1. Adverse Events (AE) and Serious Adverse Events (SAEs)

The definitions of an AE or SAE can be found in Appendix 3.

The investigator (or investigator) and their designees are responsible for detecting, documenting and reporting events that meet the definition of an AE or SAE.

7.4.1.1. Time Period and Frequency for Collecting AE and SAE Information

- AEs and SAEs will be collected from the start of treatment with the thermostable formulation of FLOLAN until the follow-up contact (see Section 7.4.1.3), at the timepoints specified in the Time and Events Table (Section 7.1).
- Medical occurrences that begin prior to the start of treatment with the thermostable formulation of FLOLAN but after obtaining informed consent may be recorded on the Medical History/Current Medical Conditions section of the CRF.
- Any SAEs assessed as related to study participation (e.g., protocol-mandated procedures, invasive tests, or change in existing therapy) or related to a GSK product will be recorded from the time a subject consents to participate in the study up to and including any follow-up contact.
- All SAEs, and all non-serious AE assessed as related to the investigational product (reformulated diluent for FLOLAN) will be recorded and reported to GSK within 24 hours, as indicated in Appendix 3.

- Investigators (or subinvestigators) are not obligated to actively seek AEs or SAEs in former study subjects. However, if the investigator (or subinvestigator) learns of any SAE, including a death, at any time after a subject has been discharged from the study, and he/she considers the event reasonably related to the investigational product (reformulated diluent for FLOLAN) or study participation, the investigator (or subinvestigator) must promptly notify GSK.

NOTE: The method of recording, evaluating and assessing causality of AEs and SAEs plus procedures for completing and transmitting SAE reports to GSK are provided in Appendix 3.

7.4.1.2. Method of Detecting AEs and SAEs

Care will be taken not to introduce bias when detecting AEs and/or SAEs. Open-ended and non-leading verbal questioning of the subject is the preferred method to inquire about AE occurrence. Appropriate questions include:

- "How are you feeling?"
- "Have you had any (other) medical problems since your last visit/contact?"
- "Have you taken any new medicines, other than those provided in this study, since your last visit/contact?"

7.4.1.3. Follow-up of AEs and SAEs

After the initial AE/SAE report, the investigator (or subinvestigator) is required to proactively follow each subject at subsequent visits/contacts. All SAEs, all non-serious AEs of special interest (as defined in Section 4.6.1), and all non-serious AE assessed as related to the investigational product (reformulated diluent for FLOLAN) will be followed until resolution, until the condition stabilizes, until the event is otherwise explained, or until the subject is lost to follow-up (as defined in Section 5.4). Further information on follow-up procedures is given in Appendix 3.

7.4.1.4. Cardiovascular and Death Events

For any cardiovascular events detailed in Appendix 3 and all deaths, whether or not they are considered SAEs, specific Cardiovascular (CV) and Death sections of the CRF will be required to be completed. These sections include questions regarding cardiovascular (including sudden cardiac death) and non-cardiovascular death.

The CV CRFs are presented as queries in response to reporting of certain CV MedDRA terms. The CV information should be recorded in the specific cardiovascular section of the CRF within one week of receipt of a CV Event data query prompting its completion.

The Death CRF is provided immediately after the occurrence or outcome of death is reported. Initial and follow-up reports regarding death must be completed within one week of when the death is reported.

7.4.1.5. Regulatory Reporting Requirements for AEs and SAEs

Prompt notification by the investigator (or subinvestigator) to GSK of SAEs, and non-serious AEs assessed as related to the investigational product (reformulated diluent for FLOLAN) is essential so that legal obligations and ethical responsibilities towards the safety of subjects and the safety of a product under clinical investigation are met.

GSK has a legal responsibility to notify both the local regulatory authority and other regulatory agencies about the safety of a product under clinical investigation. GSK will comply with country specific regulatory requirements relating to safety reporting to the regulatory authority, Institutional Review Board (IRB)/Independent Ethics Committee (IEC) and investigators.

Investigator safety reports are prepared for suspected unexpected serious adverse reactions according to local regulatory requirements and GSK policy and are forwarded to investigators as necessary.

An investigator who receives an investigator safety report describing a SAE(s) or other specific safety information (e.g., summary or listing of SAEs) from GSK will file it with the IB and will notify the IRB/IEC, if appropriate according to local requirements.

7.4.2. Pregnancy

- Details of all pregnancies in female subjects and female partners of male subjects will be collected after the start of treatment with the thermostable formulation of FLOLAN and until delivery (including pregnancies resulting in early termination).

- If a pregnancy is reported then the investigator (or subinvestigator) should inform GSK within 2 weeks of learning of the pregnancy and should follow the procedures outlined in Appendix 4.

7.4.3. Physical Exams

Physical examinations will be conducted at baseline (height and weight) Week 4 (weight only) and, if applicable, early withdrawal visit (weight only).Investigators (or subinvestigators) should pay special attention to clinical signs related to previous serious illnesses.

7.4.4. Vital Signs

Vital signs will be measured in semi-supine position after 5 minutes rest and will include systolic and diastolic blood pressure and heart rate.

7.4.5. Oxygen Saturation

Oxygen saturation will be measured by pulse oximetry.

7.4.6. Electrocardiogram (ECG)

A 12-lead ECG will be done in a supine position after having kept a subject at rest in this position for about 5 minutes at each assessment time.

7.4.7. Clinical Safety Laboratory Assessments

The following clinical laboratory test parameters will be measured at the institution's local laboratory. Results of each assessment are recorded in the CRF.

Laboratory Assessments	Parameters
Haematology	Haemoglobin, hematocrit, RBC count, WBC count (including fractions), platelet count
Clinical Chemistry [1]	ALT(GPT), AST(GOT), Al-P, γ-GTP, LDH, CPK, total bilirubin, direct bilirubin, creatinine, Na, Mg, K, Cl, inorganic phosphorus, calcium, BUN, uric acid, glucose, total protein, albumin, thyroid function tests (T3, T4, TSH)
Routine Urinalysis (dip stick)	Protein, sugar, occult blood
Pregnancy tests	hCG (only women of childbearing potential)

1. Details of Liver Chemistry Stopping Criteria and Required Actions and Follow-Up Assessments after liver stopping or monitoring event are given in Appendix 2.

If additional non-protocol specified laboratory assessments are performed at the institution’s local laboratory and result in a change in subject management or are considered clinically significant by the investigator (or subinvestigator) (e.g., SAE or AE or dose modification) the results must be recorded in the CRF.

7.5. Other Endpoints

7.5.1. Reasons for Dose Adjustment after Switch

A dose adjustment, if required after switching to the reformulated diluent for FLOLAN, will be recorded in the CRF. A dose adjustment, if required in a subject undergoing RHC based on mPAP values measured during the 3 hours after switching, will be recorded in the CRF.

8. DATA MANAGEMENT

- For this study subject data will be collected using GSK defined case report forms and combined with data provided from other sources in a validated data system.
- Management of clinical data will be performed in accordance with applicable GSK standards and data cleaning procedures to ensure the integrity of the data, e.g., removing errors and inconsistencies in the data.

- Adverse events and concomitant medications terms will be coded using MedDRA (Medical Dictionary for Regulatory Activities) and an internal validated medication dictionary, GSKDrug.
- Original CRFs (including queries and audit trails) will be retained by GSK, while the investigator will retain a copy. Subject initials will not be collected or transmitted to GSK according to GSK policy.

9. STATISTICAL CONSIDERATIONS AND DATA ANALYSES

9.1. Hypotheses

Data will be summarized using descriptive statistics. No formal hypothesis tests are planned.

9.2. Sample Size Considerations

9.2.1. Sample Size Assumptions

The sample size for this study is based on the feasibility, not based on hypothesis testing.

9.2.2. Sample Size Sensitivity

The sample size for this study is based on the feasibility. No sample size sensitivity testing has been conducted.

9.2.3. Sample Size Re-estimation or Adjustment

No sample size re-estimation is planned.

9.3. Data Analysis Considerations

9.3.1. Analysis Populations

The Intention-to-Treat (ITT) population will consist of all subjects who received at least one dose of the thermostable formulation of FLOLAN. The ITT population will be used for all efficacy and safety summaries.

9.3.2. Analysis Data Sets

Analysis datasets will consist of all data collected in the study and evaluated according to the analysis populations.

9.3.3. Treatment Comparisons

No formal comparisons are planned for this study.

9.3.4. Interim Analysis

No interim analysis is planned.

9.4. Key Elements of Analysis Plan

The Analysis Plan will consist of summary and graphical displays. Individual patient listings will also be included. Full details will be described in a “Reporting and Analysis Plan”. Data will be summarized using a tabular display wherever possible according to the GSK reporting standard.

9.4.1. Primary Analyses

Safety (AEs, clinical laboratory tests [haematology, clinical chemistry and urinalysis], vital signs [systolic and diastolic blood pressure, cardiac output], 12-lead ECG, oxygen saturation) and tolerability will be assessed on all subjects who received at least one dose of the thermostable formulation of FLOLAN. For AEs, the number and percentage of subjects with AE will be presented. For SAEs and AEs resulting in treatment discontinuation, AEs by severity and AEs by causal relationship, the number and percentage of subjects with AE will be presented. All AEs will be coded using the preferred terms (PT) and grouped into system organ classes (SOC) according to the Medical Dictionary for Regulatory Activities (MedDRA).

For clinical laboratory tests, vital signs, oxygen saturation and 12-lead ECG, summary statistics by visit will be calculated. For clinical laboratory tests, the number and percentage of subjects with values outside the normal range of clinical interest will be tabulated by visit. For 12-lead ECG findings, frequency tabulation by visit will be provided.

9.4.2. Secondary Analyses

In order to assess necessity of dose adjustment in subjects after switching to the thermostable formulation of FLOLAN, the frequency of dose adjustment requirements will be tabulated based on the changes from baseline in mPAP up to 3 hours after dosing. Reasons for dose adjustment after switching to the thermostable formulation of FLOLAN will be listed.

In order to assess continued efficacy after switching to the thermostable formulation of FLOLAN, summary statistics will be provided for the change from baseline to Week 4 in NT-proBNP and the changes from baseline up to 24 hours after dosing and at Week 4 for the following haemodynamic parameters: mPAP, PVR, CO and RAP. Also frequency of WHO Functional Class by visits and change from previous visit will be provided.

9.4.3. Other Analyses

All other data (demography, medical history, disease history, therapeutic history, concomitant medications, exposure, compliance to study treatment, infusion site assessment) will be summarized descriptively.

10. STUDY GOVERNANCE CONSIDERATIONS

10.1. Posting of Information on Publicly Available Clinical Trial Registers

Study information from this protocol will be posted on publicly available clinical trial registers before enrollment of subjects begins.

10.2. Regulatory and Ethical Considerations, Including the Informed Consent Process

Prior to initiation of a site, GSK will obtain favourable opinion/approval from the appropriate regulatory agency to conduct the study in accordance with ICH Good Clinical Practice (GCP) and applicable country-specific regulatory requirements.

The study will be conducted in accordance with all applicable regulatory requirements, and with GSK policy.

The study will also be conducted in accordance with ICH Good Clinical Practice (GCP), all applicable subject privacy requirements, and the guiding principles of the current version of the Declaration of Helsinki. This includes, but is not limited to, the following:

- IRB/IEC review and favorable opinion/approval of the study protocol and amendments as applicable.
- Obtaining signed informed consent from each subject before participating in the study (for amendments as applicable).
- Investigator reporting requirements (e.g. reporting of AEs/SAEs/protocol deviations to IRB/IEC).
- GSK will provide full details of the above procedures, either verbally, in writing, or both.

The procedure for obtaining signed informed consent is as follows:

- Prior to participation in the study, the investigator (or subinvestigator) should fully inform the potential subject of the study (and/or his/her proxy consenter) including the written information. The investigator (or subinvestigator) should provide the subject (and/or his/her proxy consenter) ample time and opportunity to inquire about details of the study. The subject (and/or his/her proxy consenter) should sign and personally date the consent form. The proxy consenter, if needed by the subject, should confirm the subject's will to participate in the study, and sign and personally date the consent form.

- The subject may consider the content of the written information at home. The person who conducted the informed consent discussion and the study collaborator giving supplementary explanation, where applicable, should sign and personally date the consent form.

- If an impartial witness is required, the witness should sign and personally date the consent form.

- The investigator (or subinvestigator) should retain this signed and dated form (and other written information) together with the source medical records, such as clinical charts (in accordance with the rules for records retention, if any, at each medical institution) and give a copy to the subject (and/or his/her proxy consenter).

10.3. Quality Control (Study Monitoring)

- In accordance with applicable regulations including GCP, and GSK procedures, GSK monitors will contact the site prior to the start of the study to review with the site staff the protocol, study requirements, and their responsibilities to satisfy regulatory, ethical, and GSK requirements.

- When reviewing data collection procedures, the discussion will also include identification, agreement and documentation of data items for which the CRF will serve as the source document.

GSK will monitor the study and site activity to verify that the:

- Data are authentic, accurate, and complete.

- Safety and rights of subjects are being protected.

- Study is conducted in accordance with the currently approved protocol and any other study agreements, GCP, and all applicable regulatory requirements.

The investigator and the head of the medical institution (where applicable) agrees to allow the monitor direct access to all relevant documents.

10.4. Quality Assurance

- To ensure compliance with GCP and all applicable regulatory requirements, GSK may conduct a quality assurance assessment and/or audit of the site records, and the regulatory agencies may conduct a regulatory inspection at any time during or after completion of the study.

- In the event of an assessment, audit or inspection, the investigator (and the head of the medical institution) must agree to grant the advisor(s), auditor(s) and inspector(s) direct access to all relevant documents and to allocate their time and the time of their staff to discuss the conduct of the study, any findings/relevant issues and to implement any corrective and/or preventative actions to address any findings/issues identified.

10.5. Study and Site Closure

- Upon completion or premature discontinuation of the study, the GSK monitor will conduct site closure activities with the investigator or site staff, as appropriate, in accordance with applicable regulations including GCP, and GSK Standard Operating Procedures.

- GSK reserves the right to temporarily suspend or prematurely discontinue this study at any time for reasons including, but not limited to, safety or ethical issues or severe non-compliance. For multicentre studies, this can occur at one or more or at all sites.

- If GSK determines such action is needed, GSK will discuss the reasons for taking such action with the investigator or the head of the medical institution (where applicable). When feasible, GSK will provide advance notification to the investigator or the head of the medical institution, where applicable, of the impending action.

- If the study is suspended or prematurely discontinued for safety reasons, GSK will promptly inform all investigators, heads of the medical institutions (where applicable) and/or institution(s) conducting the study. GSK will also promptly inform the relevant regulatory authorities of the suspension or premature discontinuation of the study and the reason(s) for the action.

- If required by applicable regulations, the investigator or the head of the medical institution (where applicable) must inform the IRB/IEC promptly and provide the reason for the suspension or premature discontinuation.

10.6. Records Retention

- Following closure of the study, the investigator or the head of the medical institution (where applicable) must maintain all site study records (except for those required by local regulations to be maintained elsewhere), in a safe and secure location.

- The records must be maintained to allow easy and timely retrieval, when needed (e.g., for a GSK audit or regulatory inspection) and must be available for review in conjunction with assessment of the facility, supporting systems, and relevant site staff.

- Where permitted by local laws/regulations or institutional policy, some or all of these records can be maintained in a format other than hard copy (e.g., microfiche, scanned, electronic); however, caution needs to be exercised before such action is taken.

- The investigator must ensure that all reproductions are legible and are a true and accurate copy of the original and meet accessibility and retrieval standards, including re-generating a hard copy, if required. Furthermore, the investigator must ensure there is an acceptable back-up of these reproductions and that an acceptable quality control process exists for making these reproductions.

- GSK will inform the investigator of the time period for retaining these records to comply with all applicable regulatory requirements. The minimum retention time will meet the strictest standard applicable to that site for the study, as dictated by any institutional requirements or local laws or regulations, GSK standards/procedures, and/or institutional requirements.

- The investigator must notify GSK of any changes in the archival arrangements, including, but not limited to, archival at an off-site facility or transfer of ownership of the records in the event the investigator is no longer associated with the site.

10.7. Provision of Study Results to Investigators, Posting of Information on Publically Available Clinical Trials Registers and Publication

Where required by applicable regulatory requirements, an investigator signatory will be identified for the approval of the clinical study report. The investigator will be provided reasonable access to statistical tables, figures, and relevant reports and will have the opportunity to review the complete study results at a GSK site or other mutually-agreeable location.

GSK will also provide the investigator with the full summary of the study results. The investigator is encouraged to share the summary results with the study subjects, as appropriate.

The procedures and timing for public disclosure of the results summary and for development of a manuscript for publication will be in accordance with GSK Policy.

11. REFERENCES

Akagi S, Nakamura K, Miyaji K, et al. Marked haemodynamic improvements by high-dose epoprostenol therapy in patients with idiopathic pulmonary arterial hypertension. Circ J. 2010 Oct;74(10): 2200-5.

Galiè N, Hoeper MM, Humbert M, et al. Guidelines for the diagnosis and treatment of pulmonary hypertension: the Task Force for the Diagnosis and Treatment of Pulmonary Hypertension of the European Society of Cardiology (ESC) and the European Respiratory Society (ERS), endorsed by the International Society of Heart and Lung Transplantation (ISHLT). Eur Heart J. 2009 Oct;30(20): 2493-537.

Hatcher RA, Trussell J, Nelson AL, Cates W Jr, Stewart F, Kowal D, editors. Contraceptive Technology. 19th edition. New York: Ardent Media, 2007(a): 24. Table 3-2

Hatcher RA, Trussell J, Nelson AL, Cates W Jr, Stewart F, Kowal D, editors. Contraceptive Technology. 19th edition. New York: Ardent Media, 2007(b): 28

Ito H, Matsubara H. Pulmonary Arterial Hypertension Treatment Manual: Latest Treatment Guidance Aiming at Complete Cure. Nankodo Co.,Ltd. Mar. 2012

McLaughlin VV, Archer SL, Badesch DB, et al. ACCF/AHA 2009 expert consensus document on pulmonary hypertension a report of the American College of Cardiology Foundation Task Force on Expert Consensus Documents and the American Heart Association developed in collaboration with the American College of Chest Physicians; American Thoracic Society, Inc.; and the Pulmonary Hypertension Association. J Am Coll Cardiol. 2009 Apr 28;53(17): 1573-619.

Ogawa A, Ejiri K, Matsubara H. Long-term patient survival with idiopathic/heritable pulmonary arterial hypertension treated at a single centre in Japan. Life Sci. 2014 Nov 24;118(2): 414-9.

Provencher S, Paruchuru P, Spezzi A, et al. Quality of life, safety and efficacy profile of thermostable flolan in pulmonary arterial hypertension. PLoS One. 2015 Mar 20;10(3): e0120657.

12. APPENDICES

12.1. Appendix 1 – Abbreviations and Trademarks

Abbreviations

AE	Adverse Event
AL-P	Alkaline Phosphatase
ALT	Alanine Aminotransferase
AST	Aspartate Aminotransferase
BUN	Blood Urea Nitrogen
CO	Cardiac Output
CPK	Creatine Phosphokinase
CRF	Case Report Form
ECG	Electrocardiogram
FDA	Food and Drug Administration
GCP	Good Clinical Practice
GSK	GlaxoSmithKline
γ-GTP	γ-Glutamyltranspeptidase
hCG	Human Chorionic Gonadotropin
ICF	Informed Consent Form
IEC	Independent Ethics Committee
IRB	Institutional Review Board
LDH	Lactate Dehydrogenase
mPAP	Mean Pulmonary Artery Pressure
NT-pro-BNP	N-Terminal pro-B-type Natriuretic Peptide
PAH	Pulmonary Arterial Hypertension
PAP	Pulmonary Artery Pressure
PDE	Phosphodiesterase
PPH	Primary Pulmonary Hypertension
PDE-5	Phosphodiesterase type 5
PGI_2	Prostacyclin
PVR	Pulmonary Vascular Resistance
RAP	Right Atrial Pressure
RHC	Right Heart Catheterization
SAE	Serious Adverse Event
SaO2	Arterial Oxygen Saturation
T3	Triiodothyronine
T4	Thyroxine
TSH	Thyroid Stimulating Hormone
WHO	World Health Organization

Trademark Information

Trademarks of the GlaxoSmithKline group of companies
FLOLAN

Trademarks not owned by the GlaxoSmithKline group of companies
None

12.2. Appendix 2: Liver Safety Required Actions and Follow up Assessments

Phase III-IV liver chemistry stopping and increased monitoring criteria have been designed to assure subject safety and evaluate liver event etiology (in alignment with the FDA premarketing clinical liver safety guidance). http://www.fda.gov/downloads/Drugs/GuidanceComplianceRegulatoryInformation/Guidances/UCM174090.pdf

Phase III-IV liver chemistry increased monitoring criteria with continued therapy

Liver Chemistry Increased Monitoring Criteria – Liver Event	
**Criteria**	**Actions**
ALT ≥3xULN with or without bilirubin ≥2xULN[1,2] and with or without symptoms believed to be related to liver injury or hypersensitivity	• Subject can continue study treatment at the discretion of the investigator (subinvestigator)<br>• Report the event to GSK **within 24 hours**<br>• Complete the liver event CRF and complete an SAE data collection tool if the event also meets the criteria for an SAE[2]<br>• Perform liver event follow up assessments at discretion of the investigator (subinvestigator)<br>◦ Viral hepatitis serology[4]<br>◦ Only in those with underlying chronic hepatitis B at study entry (identified by positive hepatitis B surface antigen) quantitative hepatitis B DNA and hepatitis delta antibody[5].<br>◦ Serum creatine phosphokinase (CPK) and lactate dehydrogenase (LDH).<br>◦ Fractionate bilirubin, if total bilirubin≥2xULN<br>◦ Obtain complete blood count with differential to assess eosinophilia<br>◦ For bilirubin ≥2xULN: Anti-nuclear antibody, anti-smooth muscle antibody, Type 1 anti-liver kidney microsomal antibodies, and quantitative total immunoglobulin G (IgG or gamma globulins), serum acetaminophen adduct HPLC assay (quantifies potential acetaminophen contribution to liver injury

	in subjects with definite or likely acetaminophen use in the preceding week [James, 2009]) and liver imaging (ultrasound, magnetic resonance, or computerised tomography) and /or liver biopsy to evaluate liver disease; complete Liver Imaging and/or Liver Biopsy CRF forms.<br>• Repeat liver chemistries (include ALT, AST, alkaline phosphatase, bilirubin) and perform liver event follow up assessments within 24 hrs if bilirubin ≥2xULN and within 72 hrs if bilirubin <2xULN<br>• Subject must return at least weekly for repeat liver chemistries (ALT, AST, alkaline phosphatase, bilirubin) until they resolve, stabilise or return to within baseline

1. Serum bilirubin fractionation should be performed if testing is available. If serum bilirubin fractionation testing is unavailable, **record presence of detectable urinary bilirubin on dipstick**, indicating direct bilirubin elevations and suggesting liver injury.
2. All events of ALT ≥ 3xULN **and** bilirubin ≥ 2xULN (>35% direct bilirubin) or ALT ≥ 3xULN **and** INR>1.5, if INR measured which may indicate severe liver injury (possible 'Hy's Law'), **must be reported as an SAE (excluding studies of hepatic impairment or cirrhosis)**; INR measurement is not required and the threshold value stated will not apply to subjects receiving anticoagulants
3. New or worsening symptoms believed to be related to liver injury (such as fatigue, nausea, vomiting, right upper quadrant pain or tenderness, or jaundice) or believed to be related to hypersensitivity (such as fever, rash or eosinophilia)
4. Includes: Hepatitis A IgM antibody; Hepatitis B surface antigen and Hepatitis B Core Antibody (IgM); Hepatitis C RNA; Cytomegalovirus IgM antibody; Epstein-Barr viral capsid antigen IgM antibody (or if unavailable, obtain heterophile antibody or monospot testing); Hepatitis E IgM antibody
5. If hepatitis delta antibody assay cannot be performed,, it can be replaced with a PCR of hepatitis D RNA virus (where needed) [Le Gal, 2005].

References

James LP, Letzig L, Simpson PM, Capparelli E, Roberts DW, Hinson JA, Davern TJ, Lee WM. Pharmacokinetics of Acetaminophen-Adduct in Adults with Acetaminophen Overdose and Acute Liver Failure. Drug Metab Dispos 2009; 37:1779-1784.

Le Gal F, Gordien E, Affolabi D, Hanslik T, Alloui C, Dény P, Gault E. Quantification of Hepatitis Delta Virus RNA in Serum by Consensus Real-Time PCR Indicates Different Patterns of Virological Response to Interferon Therapy in Chronically Infected Patients. J Clin Microbiol. 2005;43(5):2363–2369.

12.3. Appendix 3: Definition of and Procedures for Recording, Evaluating, Follow-Up and Reporting of Adverse Events

12.3.1. Definition of Adverse Events

**Adverse Event Definition:**
• An AE is any untoward medical occurrence in a patient or clinical investigation subject, temporally associated with the use of a medicinal product, whether or not considered related to the medicinal product.<br>• NOTE: An AE can therefore be any unfavorable and unintended sign (including an abnormal laboratory finding), symptom, or disease (new or exacerbated) temporally associated with the use of a medicinal product.

**Events <u>meeting</u> AE definition include:**
• Any abnormal laboratory test results (hematology, clinical chemistry, or urinalysis) or other safety assessments (e.g., ECGs, radiological scans, vital signs measurements), including those that worsen from baseline, and felt to be clinically significant in the medical and scientific judgement of the investigator (subinvestigator).<br>• Exacerbation of a chronic or intermittent pre-existing condition including either an increase in frequency and/or intensity of the condition.<br>• New conditions detected or diagnosed after study treatment administration even though it may have been present prior to the start of the study.<br>• Signs, symptoms, or the clinical sequelae of a suspected interaction.<br>• Signs, symptoms, or the clinical sequelae of a suspected overdose of either study treatment or a concomitant medication (overdose per se will not be reported as an AE/SAE unless this is an intentional overdose taken with possible suicidal/self-harming intent. This should be reported regardless of sequelae).<br>• "Lack of efficacy" or "failure of expected pharmacological action" per se will not be reported as an AE or SAE. However, the signs and symptoms and/or clinical sequelae resulting from lack of efficacy will be reported if they fulfil the definition of an AE or SAE.

**Events <u>NOT</u> meeting definition of an AE include:**
• Any clinically significant abnormal laboratory findings or other abnormal safety assessments which are associated with the underlying disease, unless judged by the investigator (subinvestigator) to be more severe than expected for the subject's condition.<br><br>• The disease/disorder being studied or expected progression, signs, or symptoms of the disease/disorder being studied, unless more severe than expected for the subject's condition.<br><br>• Medical or surgical procedure (e.g., endoscopy, appendectomy): the condition that leads to the procedure is an AE.<br><br>• Situations where an untoward medical occurrence did not occur (social and/or convenience admission to a hospital).<br><br>• Anticipated day-to-day fluctuations of pre-existing disease(s) or condition(s) present or detected at the start of the study that do not worsen.

12.3.2. Definition of Serious Adverse Events

If an event is not an AE per definition above, then it cannot be an SAE even if serious conditions are met (e.g., hospitalization for signs/symptoms of the disease under study, death due to progression of disease, etc).

**Serious Adverse Event (SAE) is defined as any untoward medical occurrence that, at any dose:**
**a. Results in death**
**b. Is life-threatening**<br><br>NOTE:<br><br>The term 'life-threatening' in the definition of 'serious' refers to an event in which the subject was at risk of death at the time of the event. It does not refer to an event, which hypothetically might have caused death, if it were more severe.
**c. Requires hospitalization or prolongation of existing hospitalization**<br><br>NOTE:<br><br>• · In general, hospitalization signifies that the subject has been detained (usually involving at least an overnight stay) at the hospital or emergency ward for observation and/or treatment that would not have been appropriate in the physician's office or out patient setting. Complications that occur during hospitalization are

AEs. If a complication prolongs hospitalization or fulfills any other serious criteria, the event is serious. When in doubt as to whether "hospitalization" occurred or was necessary, the AE should be considered serious.

- · Hospitalization for elective treatment of a pre-existing condition that did not worsen from baseline is not considered an AE.

d. Results in disability/incapacity

NOTE:

- The term disability means a substantial disruption of a person's ability to conduct normal life functions.
- This definition is not intended to include experiences of relatively minor medical significance such as uncomplicated headache, nausea, vomiting, diarrhea, influenza, and accidental trauma (e.g. sprained ankle) which may interfere or prevent everyday life functions but do not constitute a substantial disruption.

e. Is a congenital anomaly/birth defect

f. Other situations:

- Medical or scientific judgment should be exercised in deciding whether reporting is appropriate in other situations, such as important medical events that may not be immediately life-threatening or result in death or hospitalization but may jeopardize the subject or may require medical or surgical intervention to prevent one of the other outcomes listed in the above definition. These should also be considered serious.
- Examples of such events are invasive or malignant cancers, intensive treatment in an emergency room or at home for allergic bronchospasm, blood dyscrasias or convulsions that do not result in hospitalization, or development of drug dependency or drug abuse.

g. Is associated with liver injury and impaired liver function defined as:

- ALT ≥ 3xULN and total bilirubin* ≥ 2xULN (>35% direct), **or**
- ALT ≥ 3xULN and INR** > 1.5.

* Serum bilirubin fractionation should be performed if testing is available; if unavailable, measure urinary bilirubin via dipstick. If fractionation is unavailable and ALT ≥ 3xULN and total bilirubin ≥ 2xULN, then the event is still to be reported as an SAE.

** INR testing not required per protocol and the threshold value does not apply to subjects receiving anticoagulants. If INR measurement is obtained, the value is to be recorded on the SAE form.

12.3.3. Definition of Cardiovascular Events

**Cardiovascular Events (CV) Definition:**
Investigators will be required to fill out the specific CV event page of the CRF for the following AEs and SAEs:<br>• Myocardial infarction/unstable angina<br>• Congestive heart failure<br>• Arrhythmias<br>• Valvulopathy<br>• Pulmonary hypertension<br>• Cerebrovascular events/stroke and transient ischemic attack<br>• Peripheral arterial thromboembolism<br>• Deep venous thrombosis/pulmonary embolism<br>• Revascularization

12.3.4. Recording of AEs and SAEs

**AEs and SAE Recording:**
• When an AE/SAE occurs, it is the responsibility of the investigator (subinvestigator) to review all documentation (e.g., hospital progress notes, laboratory, and diagnostics reports) relative to the event.<br><br>• The investigator (subinvestigator) will then record all relevant information regarding an AE/SAE in the CRF.<br><br>• It is **not** acceptable for the investigator (subinvestigator) to send photocopies of the subject's medical records to GSK in lieu of completion of the GSK, AE/SAE CRF page.<br><br>• There may be instances when copies of medical records for certain cases are requested by GSK. In this instance, all subject identifiers, with the exception of the subject number, will be blinded on the copies of the medical records prior to submission of to GSK.<br><br>• The investigator (or subinvestigator) will attempt to establish a diagnosis of the event based on signs, symptoms, and/or other clinical information. In such cases, the diagnosis will be documented as the AE/SAE and not the individual signs/symptoms.

12.3.5. Evaluating AEs and SAEs

**Assessment of Intensity**
The investigator (subinvestigator) will make an assessment of intensity for each AE and SAE reported during the study and will assign it to one of the following categories:<br><br>• Mild: An event that is easily tolerated by the subject, causing minimal discomfort and not interfering with everyday activities.<br><br>• Moderate: An event that is sufficiently discomforting to interfere with normal everyday activities.<br><br>• Severe: An event that prevents normal everyday activities. - an AE that is assessed as severe will not be confused with an SAE. Severity is a category utilized for rating the intensity of an event; and both AEs and SAEs can be assessed as severe.<br><br>• An event is defined as 'serious' when it meets at least one of the pre-defined outcomes as described in the definition of an SAE.

**Assessment of Causality**
• The investigator (subinvestigator) is obligated to assess the relationship between study treatment and the occurrence of each AE/SAE.<br><br>• A "reasonable possibility" is meant to convey that there are facts/evidence or arguments to suggest a causal relationship, rather than a relationship cannot be ruled out.<br><br>• The investigator (subinvestigator) will use clinical judgment to determine the relationship.<br><br>• Alternative causes, such as natural history of the underlying diseases, concomitant therapy, other risk factors, and the temporal relationship of the event to the study treatment will be considered and investigated.<br><br>• The investigator (subinvestigator) will also consult the Investigator Brochure (IB) and/or Product Information, for marketed products, in the determination of his/her assessment.<br><br>• For each AE/SAE the investigator (subinvestigator) **must** document in the medical notes that he/she has reviewed the AE/SAE and has provided an assessment of causality.<br><br>• There may be situations when an SAE has occurred and the investigator (subinvestigator) has minimal information to include in the initial report to GSK. However, **it is very important that the investigator (or subinvestigator) always make an assessment of causality for every event prior to the initial transmission of the SAE data to GSK.**<br><br>• The investigator (subinvestigator) may change his/her opinion of causality in light of follow-up information, amending the SAE data collection tool accordingly.<br><br>• The causality assessment is one of the criteria used when determining regulatory reporting requirements.

Follow-up of AEs and SAEs
• The investigator (subinvestigator) is obligated to perform or arrange for the conduct of supplemental measurements and/or evaluations as may be indicated or as requested by GSK to elucidate as fully as possible the nature and/or causality of the AE or SAE.<br>• The investigator (subinvestigator) is obligated to assist. This may include additional laboratory tests or investigations, histopathological examinations or consultation with other health care professionals.<br>• If a subject dies during participation in the study or during a recognized follow-up period, the investigator (subinvestigator) will provide GSK with a copy of any post-mortem findings, including histopathology.<br>• New or updated information will be recorded in the originally completed CRF.<br>• The investigator (subinvestigator) will submit any updated data on SAEs and on non-serious AEs assessed as related to the investigational product (reformulated diluent for FLOLAN) to GSK within the designated reporting time frames.

12.3.6. Reporting of SAEs and non-serious AEs assessed as related to the investigational product (reformulated diluent for FLOLAN) to GSK

SAE and non-serious AE assessed as related to the investigational product (reformulated diluent for FLOLAN) reporting to GSK via electronic data collection tool
• Primary mechanism for reporting SAEs and non-serious AEs assessed as related to the investigational product (reformulated diluent for FLOLAN) to GSK will be the electronic data collection tool.<br>• If the electronic system is unavailable for greater than 24 hours, the site will use the paper SAE and non-serious AE assessed as related to the investigational product (reformulated diluent for FLOLAN) data collection tool and fax it to the Medical Monitor or the contact information.<br>• Site will enter the serious adverse event and non-serious AE assessed as related to the investigational product (reformulated diluent for FLOLAN) data into the electronic system as soon as it becomes available.<br>• After the study is completed at a given site, the electronic data collection tool (e.g., InForm system) will be taken off-line to prevent the entry of new data or changes to existing data.

- If a site receives a report of a new SAE and non-serious AE assessed as related to the investigational product (reformulated diluent for FLOLAN) from a study subject or receives updated data on a previously reported SAE and non-serious AE assessed as related to the investigational product (reformulated diluent for FLOLAN) after the electronic data collection tool has been taken off-line, the site can report this information on a paper SAE and non-serious AE assessed as related to the investigational product (reformulated diluent for FLOLAN) form or to the Medical Monitor or the contact information by telephone.
- Contacts for SAE and non-serious AE assessed as related to the investigational product (reformulated diluent for FLOLAN) receipt can be found at the beginning of this protocol on the Sponsor/Medical Monitor Contact Information page.

12.4. Appendix 4: Collection of Pregnancy Information

- Investigator (or subinvestigator) will collect pregnancy information on any female subject, who becomes pregnant while participating in this study.

- Information will be recorded on the appropriate form and submitted to GSK within 2 weeks of learning of a subject's pregnancy.

- Subject will be followed to determine the outcome of the pregnancy. The investigator (or subinvestigator) will collect follow up information on mother and infant, which will be forwarded to GSK. Generally, follow-up will not be required for longer than 6 to 8 weeks beyond the estimated delivery date.

- Any termination of pregnancy will be reported, regardless of fetal status (presence or absence of anomalies) or indication for procedure.

- While pregnancy itself is not considered to be an AE or SAE, any pregnancy complication or elective termination of a pregnancy will be reported as an AE or SAE.

- A spontaneous abortion is always considered to be an SAE and will be reported as such.

- Any SAE occurring as a result of a post-study pregnancy which is considered reasonably related to the study treatment by the investigator (or subinvestigator), will be reported to GSK as described in Appendix 4. While the investigator (or subinvestigator) is not obligated to actively seek this information in former study participants, he or she may learn of an SAE through spontaneous reporting.

Any female subject who becomes pregnant while participating

- will discontinue study medication or be withdrawn from the study.

12.5. Appendix 5 - Japan Specific Requirements

12.5.1. Regulatory and Ethical Considerations

The study will be conducted in accordance with the Ministerial Ordinance on the Standards for the Conduct of Clinical Trials of Medicinal Products (MHW Notification No.28 dated March 27, 1997) and the Pharmaceutical and Medical Device Act (PMD Act).

12.5.2. Informed Consent

Prior to participation in the study, the investigator (or subinvestigator) should fully inform the potential subject of the study including the written information. The investigator (or subinvestigator) should provide the subject and his/her proxy consenter ample time and opportunity to inquire about details of the study. The subject and/or his/her proxy consenter should sign and personally date the consent form. The subject may wish to consider the content of the written information at home. The person who conducted the informed consent discussion and the study collaborator giving supplementary explanation, where applicable, should sign and personally date the consent form. If an impartial witness is required, the witness should sign and personally date the consent form. The investigator (or subinvestigator) should retain this signed and dated form (and other written information) together with the source medical records, such as clinical charts (in accordance with the rules for records retention, if any, at each medical institution) and give a copy to the subject and/or his/her proxy consenter.

12.5.3. Study Period

April to August 2016 (planned)

12.6. Appendix 6 - List of Annexes

Annex 1: Study Administrative Structure

Annex 2: List of Medical Institutions and Investigators

Annex 3: Pregnancy Notification Form

Annex 4: Pregnancy Follow-up Form

12.7. Appendix 7 - Protocol Amendment Changes

Protocol Amendment Number 01

Specific Changes in the Text

Medical monitor/sponsor information page, p.3

Original text

Medical Monitor/SAE Contact Information:

Role	Name	Day Time Phone Number and email address	After-hours Phone/Cell/ Pager Number	Fax Number	Site Address
Primary Medical Monitor and SAE contact information	PPD	PPD<br>PPD	Not applicable	PPD<br>PPD	6-15, Sendagaya 4-chome, Shibuya-ku, Tokyo 151-8566
Secondary Medical Monitor	PPD	PPD<br>PPD	Not applicable	PPD<br>PPD	6-15, Sendagaya 4-chome, Shibuya-ku, Tokyo 151-8566

Amended text

Medical Monitor/SAE <u>and non-serious AE assessed as related to the investigational product</u> Contact Information:

Role	Name	Day Time Phone Number and email address	After-hours Phone/Cell/ Pager Number	Fax Number	Site Address
Primary Medical Monitor	PPD	PPD<br>PPD	Not applicable	PPD<br>PPD	6-15, Sendagaya 4-chome, Shibuya-ku, Tokyo 151-8566

Secondary Medical Monitor	PPD	PPD<br>PPD	Not applicable	PPD<br>PPD	6-15, Sendagaya 4-chome, Shibuya-ku, Tokyo 151-8566
<u>Contact information</u>	<u>Refer to Annex 1: Study Administrative Structure</u>				

Original text

Sponsor Legal Registered Address:

GlaxoSmithKline K.K. (GSK Japan)

6-15, Sendagaya 4-chome

Shibuya-ku, Tokyo 151-8566, Japan

Study Director: PPD Head, MPC TA Office, Medicines Development

Amended text

<u>None</u>

Investigator protocol agreement page, p.4

Original text

None

Amended text

<u>For protocol number FLR201614</u>

Objectives/Endpoints, p.8

3.OBJECTIVES AND ENDPOINTS, p.12

Original text

Objectives	Endpoints
**Secondary**	
• To evaluate the continued efficacy after switching to the thermostable formulation of FLOLAN.	• Change from baseline to Week4 in NT pro BNP and WHO Functional Class<br><br>• Changes from baseline up to 24 hours and at Week 4 in Haemodynamic parameters: mean Pulmonary Artery Pressure (mPAP), Pulmonary Vascular resistance (PVR), Cardiac Output (CO), Right Atrial Pressure (RAP) in a subset of subjects*

Amended text

Objectives	Endpoints
**Secondary**	
• To evaluate the continued efficacy after switching to the thermostable formulation of FLOLAN.	• Change from baseline to Week4 in NT pro BNP<br><br>• World Health Organization (WHO) Functional Class frequency by visits and change from previous visit<br><br>• Changes from baseline up to 24 hours and at Week 4 in Haemodynamic parameters: mean Pulmonary Artery Pressure (mPAP), Pulmonary Vascular resistance (PVR), Cardiac Output (CO), Right Atrial Pressure (RAP) in a subset of subjects*

Analysis, p.10

Original text

Primary analyses will be frequency tabulation or summary statistics calculation for safety (AEs, clinical laboratory tests [haematology, clinical chemistry and urinalysis], vital signs [systolic and diastolic blood pressure, CO], 12-lead ECG, oxygen saturation) and tolerability on the ITT population.

Secondary analyses will be tabulation of the number of FLOLAN dose adjustment events as well as calculation of summary statistics for the change from baseline for other secondary endpoints.

Amended text

Primary analyses will be frequency tabulation or summary statistics calculation for safety (AEs, clinical laboratory tests [haematology, clinical chemistry and urinalysis], vital signs [systolic and diastolic blood pressure, heart rate], 12-lead ECG, oxygen saturation) and tolerability on the ITT population.

Secondary analyses will be tabulation of the number of FLOLAN dose adjustment events and WHO Functional Class frequency by visits and change from previous visit, as well as calculation of summary statistics for the change from baseline for other secondary endpoints.

5.3 Screening Failures, p.22

Original text

5.3. Screening/Baseline/Run-in Failures

Amended text

5.3 Screening Failures

6.1 Investigational Product, p.24

Original text

The term 'investigational product (IP)' is used through the protocol to describe the reformulated diluent for FLOLAN.

<table>
<tr><td>Product name:</td><td>FLOLAN for injection 0.5 mg</td><td>FLOLAN for injection 1.5 mg</td><td>Reformulated diluent for FLOLAN:
(Investigational Product: IP)</td></tr>
<tr><td>Ingredient*/content (as epoprostenol)</td><td>0.531mg (0.5 mg)</td><td>1.593 mg (1.5 mg)</td><td>One vial contains glycine (94 mg), sodium chloride, and sodium hydroxide (pH adjusters).</td></tr>
<tr><td>Excipients (quantity per vial)</td><td colspan="2">D-mannitol (50 mg), glycine (3.76 g), sodium chloride, and sodium hydroxide (pH adjusters)</td><td>Glycine (94 mg), sodium chloride, and sodium hydroxide (pH adjusters)</td></tr>
<tr><td rowspan="2">Physical description:</td><td colspan="2">White lyophylised powder or lump powder for injection</td><td></td></tr>
<tr><td colspan="3">The pH, osmotic and pressure ratio of the resultant solution when one vial of FLOLAN (0.5 mg, 1.5 mg) is dissolved in 50 mL of the diluent for FLOLAN and the description of the resultant solution when one vial of FLOLAN (0.5 mg, 1.5 mg) is dissolved in 10 mL of the diluent for FLOLAN are as follows:

pH, 11.7 to 12.3; osmotic pressure ratio, 0.3 to 0.5; description, colorless and clear</td></tr>
</table>

* Epoprostenol sodium

Amended text

<table>
<tr><td>Product name:</td><td>FLOLAN for injection 0.5 mg</td><td>FLOLAN for injection 1.5 mg</td><td>Reformulated diluent for FLOLAN</td></tr>
<tr><td>Ingredient*/content (as epoprostenol)</td><td>0.531mg (0.5 mg)</td><td>1.593 mg (1.5 mg)</td><td>One vial contains glycine (94 mg), sodium chloride, and sodium hydroxide (pH adjusters).</td></tr>
<tr><td>Excipients (quantity per vial)</td><td colspan="2">D-mannitol (50 mg), glycine (3.76 g), sodium chloride, and sodium hydroxide (pH adjusters)</td><td>Glycine (94 mg), sodium chloride, and sodium hydroxide (pH adjusters)</td></tr>
</table>

Physical description:	White lyophylised powder or lump powder for injection	
	The pH, osmotic and pressure ratio of the resultant solution when one vial of FLOLAN (0.5 mg, 1.5 mg) is dissolved in 50 mL of the diluent for FLOLAN and the description of the resultant solution when one vial of FLOLAN (0.5 mg, 1.5 mg) is dissolved in 10 mL of the diluent for FLOLAN are as follows:<br><br>pH, 11.7 to 12.3; osmotic pressure ratio, 0.3 to 0.5; description, colorless and clear	

* Epoprostenol sodium

7.1 Time and Events Table, p.21

Original text

Haemodynamics (RHC) Early withdrawal: X

SAE review Visit1: X[6]

Pregnancy test[7]

Amended text

Haemodynamics (RHC) Early withdrawal: X[6]

SAE review Visit1: X[7]

Pregnancy test[8]

Original text

6. Any SAEs assessed as related to study participation or related to a GSK product will be collected from the time a subject consents to participate in the study.

7. Urinary or serum test for women of childbearing potential

Amended text

6. Undergo if possible

7. Any SAEs assessed as related to study participation or related to a GSK product will be collected from the time a subject consents to participate in the study.

8. Urinary or serum test for women of childbearing potential

7.4.1.1. Time Period and Frequency for Collecting AE and SAE Information, p.33

Original text

- All SAEs will be recorded and reported to GSK within 24 hours, as indicated in Appendix 3.

Amended text

- All SAEs, and all non-serious AE assessed as related to the investigational product (reformulated diluent for FLOLAN) will be recorded and reported to GSK within 24 hours, as indicated in Appendix 3.

7.4.1.3. Follow-up of AEs and SAEs, p.34

Original text

All SAEs, and non-serious AEs of special interest (as defined in Section 4.6.1) will be followed until resolution, until the condition stabilizes, until the event is otherwise explained, or until the subject is lost to follow-up (as defined in Section 5.4).

Amended text

All SAEs, all non-serious AEs of special interest (as defined in Section 4.6.1), and all non-serious AE assessed as related to the investigational product (reformulated diluent for FLOLAN) will be followed until resolution, until the condition stabilizes, until the event is otherwise explained, or until the subject is lost to follow-up (as defined in Section 5.4).

7.4.1.5. Regulatory Reporting Requirements for AEs and SAEs, p.35

Original text

7.4.1.5. Regulatory Reporting Requirements for SAEs

Amended text

7.4.1.5. Regulatory Reporting Requirements for AEs and SAEs

Original text

Prompt notification by the investigator (or subinvestigator) to GSK of SAEs related to investigational product (even for non-interventional post-marketing studies) is essential so that legal obligations and ethical responsibilities towards the safety of subjects and the safety of a product under clinical investigation are met.

Amended text

Prompt notification by the investigator (or subinvestigator) to GSK of SAEs, and non-serious AEs assessed as related to the investigational product (reformulated diluent for FLOLAN) is essential so that legal obligations and ethical responsibilities towards the safety of subjects and the safety of a product under clinical investigation are met.

9.4.2 Secondary Analyses, p.39

Original text

In order to assess continued efficacy after switching to the thermostable formulation of FLOLAN, summary statistics will be provided for the change from baseline to Week 4 in NT-proBNP, WHO Functional Class distribution at Week 4, and the changes from baseline up to 24 hours after dosing and at Week 4 for the following haemodynamic parameters: mPAP, PVR, CO and RAP.

Amended text

In order to assess continued efficacy after switching to the thermostable formulation of FLOLAN, summary statistics will be provided for the change from baseline to Week 4 in NT-proBNP and the changes from baseline up to 24 hours after dosing and at Week 4 for the following haemodynamic parameters: mPAP, PVR, CO and RAP. Also frequency of WHO Functional Class by visits and change from previous visit will be provided.

10.2. Regulatory and Ethical Considerations, Including the Informed Consent Process, p.40

Original text

- If the subject wishes to consider the content of the written information at home, he/she may sign the consent form at home.

Amended text

- The subject may consider the content of the written information at home.

12.1 Appendix 1 – Abbreviations and Trademarks, p.45

Original text

ACCP: American College of Chest Physician

SaO_2: Arterial Oxygen Saturation

CI: Cardiac Index

Amended text

None

SaO2: Arterial Oxygen Saturation

CO: Cardiac Output

12.3.5. Evaluating AEs and SAEs, Follow-up of AEs and SAEs, p.54

Original text

- The investigator (subinvestigator) will submit any updated SAE data to GSK within the designated reporting time frames.

Amended text

- The investigator (subinvestigator) will submit any updated data on SAEs and on non-serious AEs assessed as related to the investigational product (reformulated diluent for FLOLAN) to GSK within the designated reporting time frames.

12.3.6. Reporting of SAEs and non-serious AEs assessed as related to the investigational product (reformulated diluent for FLOLAN) to GSK, p.55

Original text

12.3.6. Reporting of SAEs to GSK

Amended text

12.3.6. Reporting of SAEs and non-serious AEs assessed as related to the investigational product (reformulated diluent for FLOLAN) to GSK

Original text

**SAE reporting to GSK via electronic data collection tool**
• Primary mechanism for reporting SAEs to GSK will be the electronic data collection tool.<br><br>• If the electronic system is unavailable for greater than 24 hours, the site will use the paper SAE data collection tool and fax it to the [Medical Monitor or the SAE coordinator].<br><br>• Site will enter the serious adverse event data into the electronic system as soon as it becomes available.<br><br>• After the study is completed at a given site, the electronic data collection tool (e.g., InForm system) will be taken off-line to prevent the entry of new data or changes to existing data.<br><br>• If a site receives a report of a new SAE from a study subject or receives updated data on a previously reported SAE after the electronic data collection tool has been taken off-line, the site can report this information on a paper SAE form or to the [Medical Monitor or the SAE coordinator] by telephone.<br><br>• Contacts for SAE receipt can be found at the beginning of this protocol on the Sponsor/Medical Monitor Contact Information page.

Amended text

**SAE and non-serious AE assessed as related to the investigational product (reformulated diluent for FLOLAN) reporting to GSK via electronic data collection tool**
• Primary mechanism for reporting SAEs and non-serious AEs assessed as related to the investigational product (reformulated diluent for FLOLAN) to GSK will be the electronic data collection tool.<br><br>• If the electronic system is unavailable for greater than 24 hours, the site will use the paper SAE and non-serious AE assessed as related to the investigational product (reformulated diluent for FLOLAN) data collection tool and fax it to the Medical Monitor or the contact information.<br><br>• Site will enter the serious adverse event and non-serious AE assessed as related to the investigational product (reformulated diluent for FLOLAN) data into the electronic system as soon as it becomes available.<br><br>• After the study is completed at a given site, the electronic data collection tool (e.g., InForm system) will be taken off-line to prevent the entry of new data or changes to

existing data.

- If a site receives a report of a new SAE and non-serious AE assessed as related to the investigational product (reformulated diluent for FLOLAN) from a study subject or receives updated data on a previously reported SAE and non-serious AE assessed as related to the investigational product (reformulated diluent for FLOLAN) after the electronic data collection tool has been taken off-line, the site can report this information on a paper SAE and non-serious AE assessed as related to the investigational product (reformulated diluent for FLOLAN) form or to the Medical Monitor or the contact information by telephone.

- Contacts for SAE and non-serious AE assessed as related to the investigational product (reformulated diluent for FLOLAN) receipt can be found at the beginning of this protocol on the Sponsor/Medical Monitor Contact Information page.

12.4 Appendix 4: Collection of Pregnancy Information, p.57

Original text

For any female partner of a male study subject who becomes pregnant while participating:

- Investigator (or subinvestigator) will attempt to collect pregnancy information on any female partner of a male study subject who becomes pregnant while participating in this study. This applies only to subjects who are randomized to receive study medication.

- After obtaining the necessary signed informed consent from the female partner directly, the investigator (or subinvestigator) will record pregnancy information on the appropriate form and submit it to GSK within 2 weeks of learning of the partner’s pregnancy

- Partner will also be followed to determine the outcome of the pregnancy. Information on the status of the mother and child will be forwarded to GSK.

- Generally, follow-up will be no longer than 6 to 8 weeks following the estimated delivery date. Any termination of the pregnancy will be reported regardless of fetal status (presence or absence of anomalies) or indication for procedure.

Amended text

None

TITLE PAGE

Division: Worldwide Development

Information Type: Protocol Amendment

Title: An Open Label, Single-arm Study Evaluating a New Thermostable Formulation of FLOLAN™ in Japanese Subjects with Pulmonary Arterial Hypertension (PAH)

Compound Number: AH21461

Development Phase: IV

Effective Date: 18-MAY-2016

Protocol Amendment Number: 02

Author(s): PPD [redacted]

Revision Chronology

GlaxoSmithKline Document Number	Date	Version
2015N240404_00	2015-DEC-03	Original
2015N240404_01	2016-FEB-17	Amendment Number 01
The primary changes reflected in Amendment Number 01 were made to modify description of secondary endpoint (WHO Fnctional Class), to add non-serious AEs assessed as related to the investigational product to AE-related sections, and to make description adjustments.		
2015N240404_02	2016-MAY-18	Amendment Number 02
The primary changes reflected in Amendment Number 02 were made to modify thyroid function test parameters and to make description adjustments.		

Copyright 2016 the GlaxoSmithKline group of companies. All rights reserved. Unauthorised copying or use of this information is prohibited.

SPONSOR SIGNATORY:

PPD

2016/5/18

Hiromu Nakajima PPD
Head, Medicines Development

Date

PPD

MEDICAL MONITOR/SPONSOR INFORMATION PAGE

Medical Monitor/SAE and non-serious AE assessed as related to the investigational product Contact Information:

Role	Name	Day Time Phone Number and email address	After-hours Phone/Cell/ Pager Number	Fax Number	Site Address
Primary Medical Monitor	PPD	PPD<br>PPD	Not applicable	PPD<br>PPD	6-15, Sendagaya 4-chome, Shibuya-ku, Tokyo 151-8566
Secondary Medical Monitor	PPD	PPD<br>PPD	Not applicable	PPD<br>PPD	6-15, Sendagaya 4-chome, Shibuya-ku, Tokyo 151-8566
Contact information	Refer to Annex 1: Study Administrative Structure				

Regulatory Agency Identifying Number(s): Not applicable

INVESTIGATOR PROTOCOL AGREEMENT PAGE

For protocol number FLR201614

- I confirm agreement to conduct the study in compliance with the protocol.
- I acknowledge that I am responsible for overall study conduct. I agree to personally conduct or supervise the described study.
- I agree to ensure that all associates, colleagues and employees assisting in the conduct of the study are informed about their obligations. Mechanisms are in place to ensure that site staff receives the appropriate information throughout the study.

Investigator Name:

Study Centre Address:

Study Centre Phone Number:

Investigator Signature: Date:

APPENDICES

Appendix 1 - Abbreviations and Trademarks

Abbreviations

AE	Adverse Event
AL-P	Alkaline Phosphatase
ALT	Alanine Aminotransferase
AST	Aspartate Aminotransferase
BUN	Blood Urea Nitrogen
CO	Cardiac Output
CPK	Creatine Phosphokinase
CRF	Case Report Form
ECG	Electrocardiogram
FDA	Food and Drug Administration
FT3	Free Triiodothyronine
FT4	Free Thyroxine
GCP	Good Clinical Practice
GSK	GlaxoSmithKline
hCG	Human Chorionic Gonadotropin
ICF	Informed Consent Form
IEC	Independent Ethics Committee
IRB	Institutional Review Board
LDH	Lactate Dehydrogenase
mPAP	Mean Pulmonary Artery Pressure
NT-pro-BNP	N-Terminal pro-B-type Natriuretic Peptide
PAH	Pulmonary Arterial Hypertension
PAP	Pulmonary Artery Pressure
PDE	Phosphodiesterase
PDE-5	Phosphodiesterase type 5
PGI_2	Prostacyclin
PPH	Primary Pulmonary Hypertension
PVR	Pulmonary Vascular Resistance
RAP	Right Atrial Pressure
RHC	Right Heart Catheterization
SAE	Serious Adverse Event
SaO2	Arterial Oxygen Saturation
TSH	Thyroid Stimulating Hormone
WHO	World Health Organization
γ-GTP	γ-Glutamyltranspeptidase

Trademark Information

Trademarks of the GlaxoSmithKline group of companies
FLOLAN

Trademarks not owned by the GlaxoSmithKline group of companies
None

Appendix 7 - Protocol Amendment Changes

Protocol Amendment Number 01

Specific Changes in the Text

Medical monitor/sponsor information page, p.3

Original text

Medical Monitor/SAE Contact Information:

Role	Name	Day Time Phone Number and email address	After-hours Phone/Cell/ Pager Number	Fax Number	Site Address
Primary Medical Monitor and SAE contact information	PPD	PPD PPD	Not applicable	PPD PPD	6-15, Sendagaya 4-chome, Shibuya-ku, Tokyo 151-8566
Secondary Medical Monitor	PPD	PPD PPD	Not applicable	PPD PPD	6-15, Sendagaya 4-chome, Shibuya-ku, Tokyo 151-8566

Amended text

Medical Monitor/SAE <u>and non-serious AE assessed as related to the investigational product</u> Contact Information:

Role	Name	Day Time Phone Number and email address	After-hours Phone/Cell/ Pager Number	Fax Number	Site Address
Primary Medical Monitor	PPD	PPD PPD	Not applicable	PPD PPD	6-15, Sendagaya 4-chome, Shibuya-ku, Tokyo 151-8566

Role	Name	Day Time Phone Number and email address	After-hours Phone/Cell/ Pager Number	Fax Number	Site Address
Secondary Medical Monitor	PPD	PPD<br>PPD	Not applicable	PPD<br>PPD	6-15, Sendagaya 4-chome, Shibuya-ku, Tokyo 151-8566
<u>Contact information</u>	<u>Refer to Annex 1: Study Administrative Structure</u>				

Original text

Sponsor Legal Registered Address:

GlaxoSmithKline K.K. (GSK Japan)

6-15, Sendagaya 4-chome

Shibuya-ku, Tokyo 151-8566, Japan

Study Director: PPD Head, MPC TA Office, Medicines Development

Amended text

<u>None</u>

Investigator protocol agreement page, p.4

Original text

None

Amended text

<u>For protocol number FLR201614</u>

Objectives/Endpoints, p.8

3. OBJECTIVES AND ENDPOINTS, p.12

Original text

Objectives	Endpoints
**Secondary**	
• To evaluate the continued efficacy after switching to the thermostable formulation of FLOLAN.	• Change from baseline to Week4 in NT pro BNP and WHO Functional Class<br>• Changes from baseline up to 24 hours and at Week 4 in Haemodynamic parameters: mean Pulmonary Artery Pressure (mPAP), Pulmonary Vascular resistance (PVR), Cardiac Output (CO), Right Atrial Pressure (RAP) in a subset of subjects*

Amended text

Objectives	Endpoints
**Secondary**	
• To evaluate the continued efficacy after switching to the thermostable formulation of FLOLAN.	• Change from baseline to Week4 in NT pro BNP<br>• World Health Organization (WHO) Functional Class frequency by visits and change from previous visit<br>• Changes from baseline up to 24 hours and at Week 4 in Haemodynamic parameters: mean Pulmonary Artery Pressure (mPAP), Pulmonary Vascular resistance (PVR), Cardiac Output (CO), Right Atrial Pressure (RAP) in a subset of subjects*

Analysis, p.10

Original text

Primary analyses will be frequency tabulation or summary statistics calculation for safety (AEs, clinical laboratory tests [haematology, clinical chemistry and urinalysis], vital signs [systolic and diastolic blood pressure, CO], 12-lead ECG, oxygen saturation) and tolerability on the ITT population.

Secondary analyses will be tabulation of the number of FLOLAN dose adjustment events as well as calculation of summary statistics for the change from baseline for other secondary endpoints.

Amended text

Primary analyses will be frequency tabulation or summary statistics calculation for safety (AEs, clinical laboratory tests [haematology, clinical chemistry and urinalysis], vital signs [systolic and diastolic blood pressure, heart rate], 12-lead ECG, oxygen saturation) and tolerability on the ITT population.

Secondary analyses will be tabulation of the number of FLOLAN dose adjustment events and WHO Functional Class frequency by visits and change from previous visit, as well as calculation of summary statistics for the change from baseline for other secondary endpoints.

5.3 Screening Failures, p.22

Original text

5.3. Screening/Baseline/Run-in Failures

Amended text

5.3 Screening Failures

6.1 Investigational Product, p.24

Original text

The term 'investigational product (IP)' is used through the protocol to describe the reformulated diluent for FLOLAN.

<table>
<tr><td>Product name:</td><td>FLOLAN for injection 0.5 mg</td><td>FLOLAN for injection 1.5 mg</td><td>Reformulated diluent for FLOLAN:
<u>(Investigational Product: IP)</u></td></tr>
<tr><td>Ingredient*/content (as epoprostenol)</td><td>0.531mg (0.5 mg)</td><td>1.593 mg (1.5 mg)</td><td>One vial contains glycine (94 mg), sodium chloride, and sodium hydroxide (pH adjusters).</td></tr>
<tr><td>Excipients (quantity per vial)</td><td colspan="2">D-mannitol (50 mg), glycine (3.76 g), sodium chloride, and sodium hydroxide (pH adjusters)</td><td>Glycine (94 mg), sodium chloride, and sodium hydroxide (pH adjusters)</td></tr>
<tr><td rowspan="2">Physical description:</td><td colspan="2">White lyophylised powder or lump powder for injection</td><td></td></tr>
<tr><td colspan="3">The pH, osmotic and pressure ratio of the resultant solution when one vial of FLOLAN (0.5 mg, 1.5 mg) is dissolved in 50 mL of the diluent for FLOLAN and the description of the resultant solution when one vial of FLOLAN (0.5 mg, 1.5 mg) is dissolved in 10 mL of the diluent for FLOLAN are as follows:

pH, 11.7 to 12.3; osmotic pressure ratio, 0.3 to 0.5; description, colorless and clear</td></tr>
</table>

* Epoprostenol sodium

Amended text

<table>
<tr><td>Product name:</td><td>FLOLAN for injection 0.5 mg</td><td>FLOLAN for injection 1.5 mg</td><td>Reformulated diluent for FLOLAN</td></tr>
<tr><td>Ingredient*/content (as epoprostenol)</td><td>0.531mg (0.5 mg)</td><td>1.593 mg (1.5 mg)</td><td>One vial contains glycine (94 mg), sodium chloride, and sodium hydroxide (pH adjusters).</td></tr>
<tr><td>Excipients (quantity per vial)</td><td colspan="2">D-mannitol (50 mg), glycine (3.76 g), sodium chloride, and sodium hydroxide (pH adjusters)</td><td>Glycine (94 mg), sodium chloride, and sodium hydroxide (pH adjusters)</td></tr>
<tr><td>Physical description:</td><td colspan="2">White lyophylised powder or lump powder for injection</td><td></td></tr>
</table>

<table>
<tr><td>Product name:</td><td>FLOLAN for injection 0.5 mg</td><td>FLOLAN for injection 1.5 mg</td><td>Reformulated diluent for FLOLAN</td></tr>
<tr><td></td><td colspan="3">The pH, osmotic and pressure ratio of the resultant solution when one vial of FLOLAN (0.5 mg, 1.5 mg) is dissolved in 50 mL of the diluent for FLOLAN and the description of the resultant solution when one vial of FLOLAN (0.5 mg, 1.5 mg) is dissolved in 10 mL of the diluent for FLOLAN are as follows:

pH, 11.7 to 12.3; osmotic pressure ratio, 0.3 to 0.5; description, colorless and clear</td></tr>
</table>

* Epoprostenol sodium

7.1 Time and Events Table, p.21

Original text

Haemodynamics (RHC) Early withdrawal: X

SAE review Visit1: X[6]

Pregnancy test[7]

Amended text

Haemodynamics (RHC) Early withdrawal: X[6]

SAE review Visit1: X[7]

Pregnancy test[8]

Original text

6. Any SAEs assessed as related to study participation or related to a GSK product will be collected from the time a subject consents to participate in the study.

7. Urinary or serum test for women of childbearing potential

Amended text

6. Undergo if possible

7. Any SAEs assessed as related to study participation or related to a GSK product will be collected from the time a subject consents to participate in the study.

8. Urinary or serum test for women of childbearing potential

7.4.1.1. Time Period and Frequency for Collecting AE and SAE Information, p.33

Original text

- All SAEs will be recorded and reported to GSK within 24 hours, as indicated in Appendix 3.

Amended text

- All SAEs, and all non-serious AE assessed as related to the investigational product (reformulated diluent for FLOLAN) will be recorded and reported to GSK within 24 hours, as indicated in Appendix 3.

7.4.1.3. Follow-up of AEs and SAEs, p.34

Original text

All SAEs, and non-serious AEs of special interest (as defined in Section 4.6.1) will be followed until resolution, until the condition stabilizes, until the event is otherwise explained, or until the subject is lost to follow-up (as defined in Section 5.4).

Amended text

All SAEs, all non-serious AEs of special interest (as defined in Section 4.6.1), and all non-serious AE assessed as related to the investigational product (reformulated diluent for FLOLAN) will be followed until resolution, until the condition stabilizes, until the event is otherwise explained, or until the subject is lost to follow-up (as defined in Section 5.4).

7.4.1.5. Regulatory Reporting Requirements for AEs and SAEs, p.35

Original text

7.4.1.5. Regulatory Reporting Requirements for SAEs

Amended text

7.4.1.5. Regulatory Reporting Requirements for AEs and SAEs

Original text

Prompt notification by the investigator (or subinvestigator) to GSK of SAEs related to investigational product (even for non-interventional post-marketing studies) is essential so that legal obligations and ethical responsibilities towards the safety of subjects and the safety of a product under clinical investigation are met.

Amended text

Prompt notification by the investigator (or subinvestigator) to GSK of SAEs, and non-serious AEs assessed as related to the investigational product (reformulated diluent for

FLOLAN) is essential so that legal obligations and ethical responsibilities towards the safety of subjects and the safety of a product under clinical investigation are met.

9.4.2 Secondary Analyses, p.39

Original text

In order to assess continued efficacy after switching to the thermostable formulation of FLOLAN, summary statistics will be provided for the change from baseline to Week 4 in NT-proBNP, WHO Functional Class distribution at Week 4, and the changes from baseline up to 24 hours after dosing and at Week 4 for the following haemodynamic parameters: mPAP, PVR, CO and RAP.

Amended text

In order to assess continued efficacy after switching to the thermostable formulation of FLOLAN, summary statistics will be provided for the change from baseline to Week 4 in NT-proBNP and the changes from baseline up to 24 hours after dosing and at Week 4 for the following haemodynamic parameters: mPAP, PVR, CO and RAP. Also frequency of WHO Functional Class by visits and change from previous visit will be provided.

10.2. Regulatory and Ethical Considerations, Including the Informed Consent Process, p.40

Original text

- If the subject wishes to consider the content of the written information at home, he/she may sign the consent form at home.

Amended text

- The subject may consider the content of the written information at home.

12.1 Appendix 1 – Abbreviations and Trademarks, p.45

Original text

ACCP: American College of Chest Physician

SaO_2: Arterial Oxygen Saturation

CI: Cardiac Index

Amended text

None

SaO2: Arterial Oxygen Saturation

CO: Cardiac Output

12.3.5. Evaluating AEs and SAEs, Follow-up of AEs and SAEs, p.54

Original text

- The investigator (subinvestigator) will submit any updated SAE data to GSK within the designated reporting time frames.

Amended text

- The investigator (subinvestigator) will submit any updated data on SAEs and on non-serious AEs assessed as related to the investigational product (reformulated diluent for FLOLAN) to GSK within the designated reporting time frames.

12.3.6. Reporting of SAEs and non-serious AEs assessed as related to the investigational product (reformulated diluent for FLOLAN) to GSK, p.55

Original text

12.3.6. Reporting of SAEs to GSK

Amended text

12.3.6. Reporting of SAEs and non-serious AEs assessed as related to the investigational product (reformulated diluent for FLOLAN) to GSK

Original text

SAE reporting to GSK via electronic data collection tool
• Primary mechanism for reporting SAEs to GSK will be the electronic data collection tool.<br>• If the electronic system is unavailable for greater than 24 hours, the site will use the paper SAE data collection tool and fax it to the [Medical Monitor or the SAE coordinator].<br>• Site will enter the serious adverse event data into the electronic system as soon as it becomes available.<br>• After the study is completed at a given site, the electronic data collection tool (e.g., InForm system) will be taken off-line to prevent the entry of new data or changes to existing data.<br>• If a site receives a report of a new SAE from a study subject or receives updated data on a previously reported SAE after the electronic data collection tool has been taken off-line, the site can report this information on a paper SAE form or to the [Medical Monitor or the SAE coordinator] by telephone.<br>• Contacts for SAE receipt can be found at the beginning of this protocol on the

Sponsor/Medical Monitor Contact Information page.

Amended text

**SAE and non-serious AE assessed as related to the investigational product (reformulated diluent for FLOLAN) reporting to GSK via electronic data collection tool**
• Primary mechanism for reporting SAEs and non-serious AEs assessed as related to the investigational product (reformulated diluent for FLOLAN) to GSK will be the electronic data collection tool.<br><br>• If the electronic system is unavailable for greater than 24 hours, the site will use the paper SAE and non-serious AE assessed as related to the investigational product (reformulated diluent for FLOLAN) data collection tool and fax it to the Medical Monitor or the contact information.<br><br>• Site will enter the serious adverse event and non-serious AE assessed as related to the investigational product (reformulated diluent for FLOLAN) data into the electronic system as soon as it becomes available.<br><br>• After the study is completed at a given site, the electronic data collection tool (e.g., InForm system) will be taken off-line to prevent the entry of new data or changes to existing data.<br><br>• If a site receives a report of a new SAE and non-serious AE assessed as related to the investigational product (reformulated diluent for FLOLAN) from a study subject or receives updated data on a previously reported SAE and non-serious AE assessed as related to the investigational product (reformulated diluent for FLOLAN) after the electronic data collection tool has been taken off-line, the site can report this information on a paper SAE and non-serious AE assessed as related to the investigational product (reformulated diluent for FLOLAN) form or to the Medical Monitor or the contact information by telephone.<br><br>• Contacts for SAE and non-serious AE assessed as related to the investigational product (reformulated diluent for FLOLAN) receipt can be found at the beginning of this protocol on the Sponsor/Medical Monitor Contact Information page.

12.4 Appendix 4: Collection of Pregnancy Information, p.57

Original text

For any female partner of a male study subject who becomes pregnant while participating:

- Investigator (or subinvestigator) will attempt to collect pregnancy information on any female partner of a male study subject who becomes pregnant while participating in this study. This applies only to subjects who are randomized to receive study medication.
- After obtaining the necessary signed informed consent from the female partner directly, the investigator (or subinvestigator) will record pregnancy information on the appropriate form and submit it to GSK within 2 weeks of learning of the partner's pregnancy
- Partner will also be followed to determine the outcome of the pregnancy. Information on the status of the mother and child will be forwarded to GSK.
- Generally, follow-up will be no longer than 6 to 8 weeks following the estimated delivery date. Any termination of the pregnancy will be reported regardless of fetal status (presence or absence of anomalies) or indication for procedure.

Amended text

None

Protocol Amendment Number 02

Specific Changes in the Text

Cover page

Original text

Author(s): PPD [redacted]

Amended text

Author(s): PPD [redacted]

Treatment Arms and Duration, p.9

4.2 Treatment Arms and Duration, p.13

Original text

The study will include a screening visit, a run-in period of a maximum of 4 weeks with the existing FLOLAN treatment (i.e., FLOLAN prepared with the currently marketed diluent), a 4-week treatment period with the thermostable formulation of FLOLAN (i.e., FLOLAN prepared with the reformulated diluent) and a one-week follow-up visit.

Amended text

The study will include a screening visit, a run-in period of a maximum of 30 days with the existing FLOLAN treatment (i.e., FLOLAN prepared with the currently marketed diluent), a 4-week treatment period with the thermostable formulation of FLOLAN (i.e., FLOLAN prepared with the reformulated diluent) and a one-week follow-up visit.

7.4.7. Clinical Safety Laboratory Assessments, p.35

Original text

Laboratory Assessments	Parameters
Clinical Chemistry [1]	ALT(GPT), AST(GOT), Al-P, γ-GTP, LDH, CPK, total bilirubin, direct bilirubin, creatinine, Na, Mg, K, Cl, inorganic phosphorus, calcium, BUN, uric acid, glucose, total protein, albumin, thyroid function tests (T3, T4, TSH)

Amended text

Laboratory Assessments	Parameters
Clinical Chemistry [1]	ALT(GPT), AST(GOT), Al-P, γ-GTP, LDH, CPK, total bilirubin, direct bilirubin, creatinine, Na, Mg, K, Cl, inorganic phosphorus, calcium, BUN, uric acid, glucose, total protein, albumin, thyroid function tests (FT3, FT4, TSH)

12.1 Appendix 1 – Abbreviations and Trademarks, p.43

Abbreviations

Original text

T3	Triiodothyronine
T4	Thyroxine

Amended text

FT3	Free Triiodothyronine
FT4	Free Thyroxine
